# Trends in Food and Beverage Portion Sizes in Australian Children; a Time-Series Analysis Comparing 2007 and 2011–2012 National Data

**DOI:** 10.3390/children4080069

**Published:** 2017-08-04

**Authors:** Daphne van der Bend, Tamara Bucher, Tracy L. Schumacher, Kate Collins, Nienke de Vlieger, Megan Rollo, Tracy L. Burrows, Jane F. Watson, Clare E. Collins

**Affiliations:** 1 School of Health Sciences, Faculty of Health and Medicine, Priority Research Centre in Physical Activity and Nutrition, The University of Newcastle, University Drive, Callaghan, NSW 2308, Australia; daphnevdbend@hotmail.com (D.v.d.B.); tamara.bucher@newcastle.edu.au (T.B.); tracy.schumacher@newcastle.edu.au (T.L.S.); nienke.devlieger@newcastle.edu.au (N.d.V.); Megan.rollo@newcastle.edu.au (M.R.); Tracy.Burrows@newcastle.edu.au (T.L.B.); 2 The New South Wales Department of Health, Northern NSW Health Districs, Maclean Community Health Centre, MacLean, NSW 2463, Australia; kate.collins@ncahs.health.nsw.gov.au (K.C.); 3 School of Education, Faculty of Education and Arts, Priority Research Centre in Physical Activity and Nutrition, The University of Newcastle, University Drive, Callaghan, NSW 2300, Australia; 4 Ethos Health, Newcastle West, NSW 2302, Australia; Jane.Watson@newcastle.edu.au (J.F.W.)

**Keywords:** 24-h recall, dietary intake, paediatric, portion size, time-series

## Abstract

In 2011–2012 approximately 26% of Australian children aged between 5–17 years were reported to be overweight or obese. Furthermore, the increase in prevalence of overweight and obesity among US children parallels reported increases in energy intake and portion sizes of common foods, leading to the recognition that availability of larger portion sizes contributes to the rise in overweight and obesity prevalence. Thus, the aim of this time-series analysis was to investigate whether selected food portion sizes in Australian children aged 2–16 years changed between 2007 and 2011–2012. Portion size data from 24-h recalls collected in Australian nutrition surveys were compared between 2007 and 2011–2012. Portion sizes changed significantly in 23% of items with increases in 15% and decreases in 8%. Changes in portion sizes varied by age, sex, and food group. Changes occurred for many meat-based items, energy-dense, nutrient-poor food items, breads, cereals, and some fruits and vegetables. Vegetable and fruit portion sizes were below the respective serving sizes of 75 g and 150 g in the Australian Guide to Healthy Eating, while portion sizes of some energy-dense, nutrient-poor foods have increased. These findings suggest approaches to increasing consumption of nutrient-dense core foods and reducing energy-dense, nutrient-poor food items in children are warranted.

## 1. Introduction

In 2011–2012, approximately 26% of Australian children aged between 5–17 years were reported to be overweight or obese [1]. Longitudinal studies in children have shown that higher intakes of energy-dense, nutrient-poor foods [2] and sugar sweetened beverages [3] contribute to weight gain. Further, the increase in prevalence of overweight and obesity among US adults and children parallels reported increases in energy intake and portion size of common foods, leading to the recognition that availability of larger portion sizes contributes to the rise in overweight and obesity prevalence [4,5]. The World Health Organisation (WHO) has recommended that providing smaller portion sizes of nutritious foods of lower energy density from an early age is an important strategy in addressing childhood obesity [6]. 

While causal links between portion size and weight status have not been confirmed [7,8], associations have been documented. In a study among pre-school children, a relationship between bigger portion sizes, energy intake, and weight status was observed, with heavier children consuming larger portions [9,10]. Portion size is associated with higher energy intakes regardless of the actual energy density of food [11]. Studies conducted in both children and adolescents have reported that larger portion sizes of particularly energy-dense foods are associated with higher total daily energy intakes [5,12,13]. Evidence for an association between portion size and energy intake was consistently found in children older than four years [14], with portion size found to explain 17–19% of the variability in child energy intake [15]. 

European and US studies in children examined portion size trends over time using routinely collected national nutrition data and concluded that portion sizes of many energy-dense, nutrient-poor items, including processed snacks, sugar sweetened beverages, and take away foods increased substantially between 1977 and 2005 [10,16,17,18,19]. In Australia, temporal trends in child and adolescent portion sizes of commonly consumed foods were not evaluated until recently. Portion size data from the 1995 National Nutrition Survey (NNS) were compared with data collected in the 2007 Australian National Children’s Nutrition and Physical Activity Survey (ANCNPAS) [20]. Between 1995 and 2007, the portion size of meat-based dishes and fruits increased, whilst portion size of foods such as soft drinks, ice cream, potato chips and chocolate, dairy foods, and vegetables decreased. Reductions in portion size were thought to be explained in part by availability of smaller sizes of numerous commercially available packaged foods in Australia [20]. 

More recent national dietary intake data were published as part of the Australian Health Survey in 2011–2012 [1]. Therefore, the current study objective is to evaluate whether portion sizes of common food items consumed by Australian children aged 2–16 have changed between 2007 and 2011‒12.

## 2. Materials and Methods

### 2.1. Study Population

The current study compared dietary data from two nationally representative cross-sectional surveys of Australian children and adolescents between 2–16 years; the 2007 ANCNPAS [21] and the 2011–2012 National Nutrition and Physical Activity Health Survey (NNPAS) [1]. 

### 2.2. Data Collection

Data collection has been described in detail elsewhere and methods used in both surveys were similar [20,22,23]. Data for both the ANCNPAS and NNPAS were collected by trained interviewers using 24-h recalls. The ANCNPAS and the NNPAS were both conducted using the computer-assisted personal interview (CAPI) and the computer assisted telephone interview (CATI) techniques [22,23]. The CATI was excluded from the current analysis in order to ensure results were comparable to results from the 1995–2007 analysis [20]. In the 2011–2012 NNPAS, dietary intake information was collected using an adapted version of the Automated Multiple-Pass Method (AMPM) developed by the Agricultural Research Service of the United States Department of Agriculture [24]. This interview process was designed with the purpose to increase respondents’ memory of the foods eaten in the last 24 hours. Food Model Booklets containing two-dimensional aids, including images of serving vessels, amorphous mounds, generic shapes, and selected foods were used to help the respondents estimate the amount they consumed [22].

### 2.3. Data Handling

**Figure 1:**
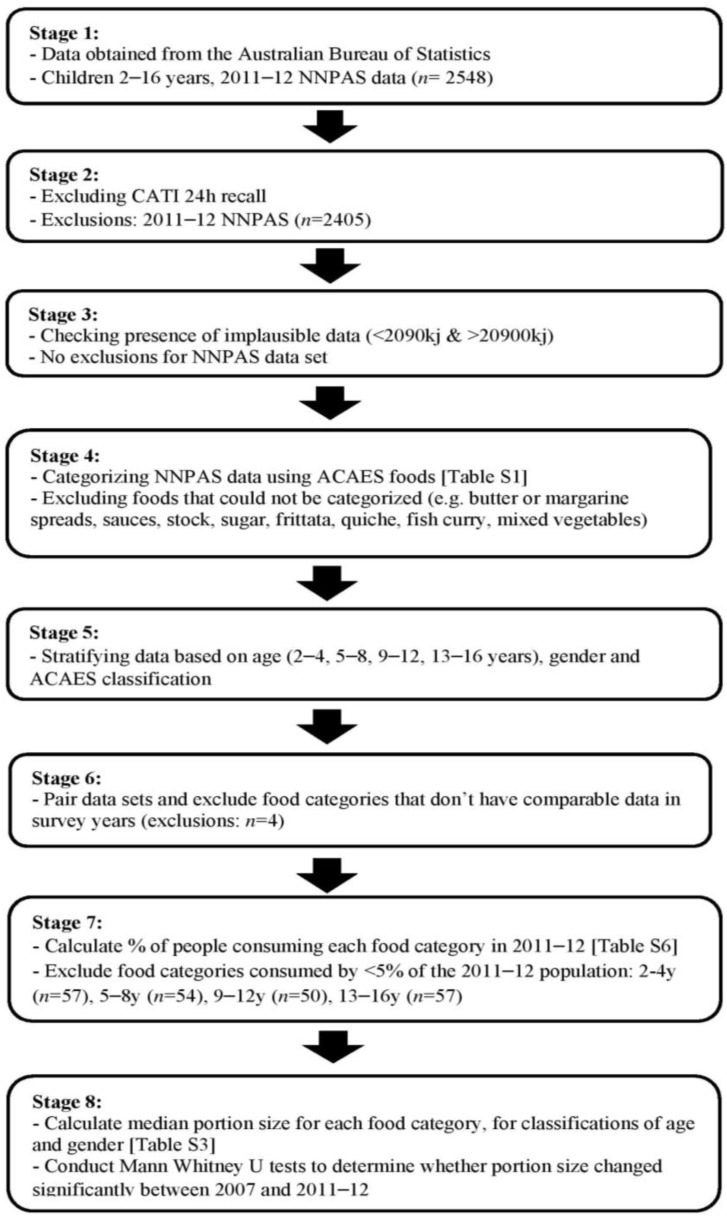
Flow chart describing data manipulation process of the 2011–2012 National Nutrition and Physical Activity Health Survey (NNPAS) data. ACAES: Australian Child and Adolescent Eating Survey.

The data handling process for ANCNPAS has been summarised previously [20]. Figure 1 illustrates data flow used to handle NNPAS data for the current analysis. Foods commonly consumed from the Australian Child and Adolescent Eating Survey (ACAES) food frequency questionnaire were used to categorize 2011–2012 data for analysis [20,25]. Similar food categories were used in the 1995–2007 analysis (see Supplementary Table S1). 

After excluding food items consumed by <5% of the 2011–2012 sample, 71 food items remained for the current comparison. The sweetened drink concentrate called ‘cordial’ was excluded as consumption was measured differently between the two surveys.

Portion size is defined as the population mean amount of a food or beverage (weight in grams) that is consumed during one eating occasion. When an individual consumes the same food or beverage on two different eating occasions on one day, such as a glass of milk, the mean of these two amounts on that day are used for that individual.

### 2.4. Data Analysis

Data analysis was conducted by sex and age category (2–4, 5–8, 9–12, 13–16 years) using STATA 11.2 (StataCorp, College Station, TX, USA). Differences in demographic characteristics were evaluated using chi-squared or independent *t*-tests. Median portion sizes for 2007 and 2011–2012 were compared using Mann–Whitney U tests (*p* < 0.05). To correct for multiple testing, a Bonferroni correction was applied, with *p*-values multiplied by the maximal number of comparisons (*n* = 71). 

## 3. Results

The sample included 7349 children aged 2–16 years from ANCNPAS (*n* = 4801) and NNPAS (*n* = 2548). Supplementary Table S2 summarises their demographic characteristics. The mean age of children between 2–4 years was significantly higher in 2011–2012 compared to 2007, while children aged 5–8 years were significantly younger in 2011–2012. The proportion of children in the different age groups did not differ significantly. In all age groups, the body mass index (BMI) status differed significantly between 2007 and 2011–2012, with a lower proportion being of normal weight in 2011–2012. However, the proportion of children with an unknown weight status reported increased substantially in 2011–2012. This is explained by a change in methodology between the two surveys: while both surveys took anthropometric measurements on a voluntary basis, the 2011–2012 survey did not collect self-reported height and weight. In the two lowest age groups, a higher proportion of 2011–2012 intakes were reported as being unusual compared to 2007. In all age groups, mean daily energy intake decreased significantly (*p* < 0.05) between 2007 and 2011–2012.

### 3.1. Portion Size Outcomes

For each age category, a total number of 57 foods (2–4-year-olds), 60 foods (5–8-year-olds), 64 foods (9–12-year-olds), and 57 foods (13–16-year-olds) were analysed (see Supplementary Table S3). Irrespective of gender, of all food portion size comparisons analysed, portion size for 77% (*n* = 185) had not changed significantly, while for 23% (*n* = 54) there was a significant change. Portion size of 15% (*n* = 36) of the foods had significantly increased, with significant reductions observed for 8% (*n* = 18).

Supplementary Table S4 shows all foods for which portion size was found to change significantly between 2007 and 2011–2012. Irrespective of gender, in the 9–12 age category, a larger number of significant changes was observed compared to other age categories. The portion size of meat-based items increased across most age groups, with plain meat and sausages significantly greater in all age groups. For all children, the portion sizes of mince dishes and mixed chicken dishes increased by 106% and 96%, respectively. Water intake increased by 50–150 mL. Foods that significantly increased across the entire child population, and in at least half of the subgroups were fruit juice, pasta, and carrots. The portion size of bacon or ham, hot chips and tomatoes significantly decreased across the whole group and in more than half of the subgroups. 

The portion size of 37 food items did not change significantly across the whole group or in any of the population subgroups. Packaged foods and several vegetables and fruits which were consumed in small portion sizes, i.e., an amount less than national intake recommendations in 2007 were in this category. In addition to the observed changes in portion size, the percentage of children consuming specific vegetables and fruits decreased between 2007 and 2011–2012. In 2011–2012 a much smaller number of vegetables was consumed by at least five percent of the sample, which resulted in a relatively large number of vegetables being excluded from the current analysis (Supplementary Tables S4 and S5).

Supplementary Table S3 shows the portion size (grams) changes between ANCNPAS 2007 and the NNPAS 2011–2012 and Supplementary Table S6 depicts the Percentage of Australian children consuming selected ACAES food categories, in the 2007 ANCNPAS and the 2011–2012 NNPAS.

## 4. Discussion

The current study investigated changes in portion size between 2007 and 2011–2012 of foods commonly consumed by Australian children aged 2–16 years. Although no definite conclusions can be drawn when comparing the changes found in the 1995–2007 analysis with those of the 2007–2011/12 analysis, given each covers a different timeframe, the current analysis provides a useful overview of recent trends in portion size relative to past analyses.

The change in median portion size of items varied by age, sex, and food groups. While the portion sizes of many meat-based dishes increased, the change in portion size for many vegetables and fruits was relatively small.

Between 2007 and 2011–2012, the portion size of a larger percentage of the foods evaluated had not significantly changed, compared to the 1995–2007 analysis (77% versus 61%). Additionally, in the 1995–2007 analysis, the portion size of a larger proportion of the foods evaluated had significantly decreased (i.e., 24%) compared to the foods evaluated in the current analysis (i.e., 8%).

The disparity between portion size of many meat-based main meal items and recommended daily intake warrants further evaluation, especially for young children (4–7 years) where the AGHE recommendation is 0.5–1 serving from the meat group per day [26]. This is important as dietary patterns are reported to track from childhood to adulthood [27]. While meat is an important source of iron, zinc, vitamin B12, and protein for adults, excessive intakes have been linked to higher risk of conditions such as colorectal cancer [28] and breast cancer [29,30,31]. 

While portion sizes of main meals prepared outside home have increased greatly [32], the current study did not make a distinction between meals prepared at home versus those consumed outside the home. However, considering this increase in meals consumed away from home [4,33], this may have contributed to an increase in reported portion sizes of both meat-based meals and pizza. Increases in portion sizes of pizza have also been observed by others [10]. 

The Australian Dietary Guidelines recommend consumption of a wide variety of vegetables and fruit [34]. Compared to 2007, a smaller variety of vegetables was consumed in 2011–2012. The total number of different vegetables consumed by at least 5% of the children in any age groups was 16 in 2007 and 10 in 2011–2012, see Supplementary Tables S3 and S4. The variety in (seasonal) fruits did not differ between 2007 and 2011–2012. Additionally, the absolute portion size of many vegetables remains small, with the portion size commonly much smaller than the recommended serving size of 75 g (vegetable) and 150 g (fruit) specified in the AGHE [26]. This has important implications for promotion not only of healthy core foods, but for the proportion and variety of core foods served within meals.

In the 1995–2007 comparison, it is suggested that the portion size of several energy-dense, nutrient-poor, and packaged foods had decreased as the result of downsizing within packaged foods sold through Australian supermarkets [20]. Further reductions were not evident based on the results of the current comparison. Since 2007, the portion size of many energy-dense packaged foods has remained stable in all population subgroups (e.g., muesli bars, potato crisps, chocolate), or increased either in the whole sample or in at least one population subgroup (e.g., flavoured milk, cakes, full-fat salad dressing). One exception was for bacon, with smaller portion sizes reported in most subgroups.

One explanation for stability of portion sizes could be that packages of many processed foods have not changed substantially since 2007, or only very recently, i.e., for several soft drinks [35]. Although some chocolate products were markedly downsized in 2009 [35], reported portion sizes of chocolate remained relatively stable. 

Despite the portion sizes remaining stable since 2007, a reduction in energy intake across all age groups was observed between 2007 and 2011–2012. Issues thought to contribute to this include differences in response rates observed in 2007 versus 2011–2012 (40% and 77%, respectively) which may have confounded misreporting bias, particularly impacting on patterns of under-reporting over time. Low Energy Reporters (LER) can be identified by comparing a person’s Basal Metabolic Rate and their reported energy intake, applying Goldberg cut-off values to determine whether the reported energy intake is plausible. It was found that between 1995 and 2011–2012, the prevalence of LER increased, as reported in the Australian Health Survey: User’s Guide, 2011–13 [22]. This was thought to explain the significant 500 kJ/day decrease in reported mean daily energy intake reported in relation to portion size changes in Australian adults between 1995 and 2011–2012 [36]. While these changes in patterns of under-reporting could partly explain the observed decrease in energy intake in the current analysis, the Goldberg cut-off is not applicable to children aged <10 years.

Young and Nestle (2002) [4] examined foods that were representative of food categories that contribute the most to daily energy intake (e.g., cakes, sodas, steak) and found that the portion sizes of many products had increased over time. However, this increase is too complex to interpret at the population level, as the magnitude of changes may not only depend on age, sex, and type of food consumed. Other factors, including socio-economic status, country-specific marketing and serving size labelling regulations, and decisions of food producers or restaurant chains are likely to also play a role [37,38]. Many foods and beverages are heavily marketed to children [11]. Proportional downsizing of palatable energy-dense foods may stimulate preferences for smaller portion sizes. Additionally, encouraging the consumption of foods with a lower energy-density, such as fruits and vegetables may increase intakes of these foods while maintaining satiety [39]. Such initiatives may eventually influence total food consumption, energy intake, or overall diet quality [7,8,39]. Combining such strategies with other interventions targeting overweight and obesity in children is important, as recent analyses have predicted a continued rise in population BMI resulting in one-third of children and adolescents aged between 5–19 years being overweight or obese by 2025 [40]. 

The current study has some limitations that should be noted. This includes limitations within the national health survey dataset and the 24-h recalls, where the portion sizes were estimated rather than weighed which may have impacted on accuracy. This has previously been described elsewhere [41,42]. Misreporting can occur and results should be interpreted with caution, particularly where interquartile ranges were large, for example in the reported portion size of water. A larger proportion of the 2011–2012 NNPAS children had a weight listed as ‘unknown’, hence the actual number of children categorized as underweight, normal, overweight, or obese may be misrepresented and therefore the results should be interpreted with caution before generalising to the Australian child population. Further, the food categorisation between years may have differed slightly due to updates within the national food database, new food product codes and changes in product variety. However, decisions in the current analysis related to specific food categories were aligned with the 1995 and 2007 foods included in Supplementary Table S1 with no major differences between food categories over that time.

Strengths of the current study include use of data from a large nationally representative sample. The methods used to measure dietary intake in 2007 and 2011–2012 were very similar, with 24-h recalls providing reliable estimates of group level intake [43]. In addition, the use of the AMPM to standardize the data collection procedure and the use of portion size aids to assist with the recall of consumption were used [24]. 

In summary, changes in portion size of commonly selected foods between 2007 and 2011–2012 varied by age, sex, and food group. Changes were observed for many meat-based items, energy-dense nutrient-poor foods, breads and cereals and some vegetables and fruits. However, the variety and portion size of many vegetables and fruits remained low. The current findings support the need for strategies to increase intakes of nutrient-dense core foods and reduce energy-dense, nutrient-poor food items in children. Potential relationships between portion size and weight status in children in future longitudinal or intervention studies is also warranted.

## Supplementary Materials

The following are available online at www.mdpi.com/2227-9067/4/8/69/S1. Table S1: Categorisation of NNS 1995, ANCNPAS 2007 and NNPAS 2011–2012 foods, Table S2: Basic demographic data of participants of the 2007 ANCNPAS and the 2011–2012 NNPAS, Table S3: portion size (grams) changes between ANCNPAS 2007 and the NNPAS 2011–2012, Table S4: Significant changes in the portion size of ACAES items between 2007 and 2011–2012, in the whole sample and for several population groups, Table S5: ACAES items that were consumed by less than 5 percent of the children in all NNPAS 2011–2012 age groups, Table S6: Percentage of Australian children consuming selected ACAES food categories, in the 2007 ANCNPAS and the 2011–2012 NNPAS.
