# Supplementary material for: Trends in Food and Beverage Portion Sizes in Australian Children; a Time-Series Analysis Comparing 2007 and 2011–2012 National Data
Source: Children (Basel). 2017 Aug 4;4(8):69. doi: 10.3390/children4080069 (PMC5575591; doi:10.3390/children4080069)
Supplement: Supplementary file 1 [file children-04-00069-s001.zip › Table S3_adj.pdf]

Table S3a: portion size (grams) changes between ANCNPAS 2007 and the NNPAS 2011–12, children 2–4 years old

	Females						Males						Persons					
	2007		2011–12				2007		2011–12				2007		2011–12			
Food	*n*	Median [IQR]	*n*	Median [IQR]	Δ Median	*p*-value[a]	*n*	Median [IQR]	*n*	Median [IQR]	Δ Median	*p*-value[a]	*n*	Median [IQR]	*n*	Median [IQR]	Δ Median	*p*-value[a]
**Drinks**																		
Soft Drink	89	152 [105]	24	151 [114]	-1	0.25	97	155 [105]	21	260 [223]	105	0.019	186	153 [105]	45	182 [135]	29	0.44
Water	1686	180 [150]	476	250 [360]	70	0.0000	1803	200 [140]	481	250 [460]	50	0.0000	3489	188 [150]	957	250 [460]	62	0.0000
Fruit Juice	492	131 [105]	165	158 [130]	27	0.0003	520	131 [150]	188	200 [131]	69	0.0000	1012	131 [114]	353	158 [130]	27	0.0000
**Dairy Foods**																		
Whole Milk	1034	155 [119]	421	155 [160]	0	0.024	1183	165 [133]	397	193 [155]	28	0.16	2217	155 [129]	818	161 [155]	6	0.0085
Reduced Fat Milk	219	130 [102]	80	146 [125]	16	0.93	221	134 [103]	59	195 [177]	61	0.12	440	130 [103]	139	156 [125]	26	0.38
Flavoured Milk	68	183 [130]	26	265 [149]	82	0.0000	80	208 [103]	29	196 [111]	-12	0.96	148	206 [129]	55	258 [153]	52	0.0031
Ice cream	126	42 [38]	44	61 [46]	19	0.093	109	48 [38]	46	56 [67]	8	0.011	235	47 [38]	90	61 [47]	14	0.0029
Yoghurt	299	100 [50]	124	100 [55]	0	0.19	315	100 [60]	118	100 [55]	0	0.11	614	100 [60]	242	100 [60]	0	0.040
Cheese	440	20 [12]	138	21 [6]	1	0.027	484	21 [13]	136	21 [8]	0	0.0021	924	20 [12]	274	21 [8]	1	0.0002
**Breads and Cereals**																		
Hot Porridge	56	133 [212]	37	104 [140]	-29	0.51	57	195 [194]	24	131 [199]	-64	0.57	113	190 [204]	61	104 [146]	-86	0.31
Breakfast Cereal	462	23 [17]	181	18 [18]	-5	0.35	521	30 [18]	184	30 [17]	0	0.088	983	26 [18]	365	25 [18]	-1	0.65
White Bread	494	37 [33]	189	48 [35]	11	0.0033	589	42 [34]	185	54 [31]	12	0.0000	1083	40 [34]	374	54 [32]	14	0.0000
Mixed Grain Bread	109	42 [34]	49	48 [42]	6	0.73	153	48 [34]	69	60 [42]	12	0.049	262	48 [34]	118	55 [42]	7	0.081
Wholemeal Bread	255	40 [32]	102	45 [37]	5	0.0061	246	48 [32]	85	50 [34]	2	0.58	501	42 [33]	187	46 [37]	4	0.019
Rice	128	95 [94]	68	67 [74]	-28	0.018	122	95 [141]	79	101 [106]	6	0.97	250	95 [97]	147	84 [95]	-11	0.15
Pasta	229	113 [111]	92	131 [139]	18	0.041	247	120 [120]	73	156 [276]	36	0.017	476	113 [114]	165	133 [187]	20	0.0018
**Sweets and Snacks**																		
Cakes	190	45 [39]	73	48 [75]	3	0.41	218	49 [47]	75	59 [105]	10	0.084	408	47 [42]	148	50 [82]	3	0.088

Table S3a: portion size (grams) changes between ANCNPAS 2007 and the NNPAS 2011–12, children 2–4 years old

	Females						Males						Persons					
	2007		2011–12				2007		2011–12				2007		2011–12			
**Food**	*n*	Median [IQR]	*n*	Median [IQR]	Δ Median	*p*-value[a]	*n*	Median [IQR]	*n*	Median [IQR]	Δ Median	*p*-value[a]	*n*	Median [IQR]	*n*	Median [IQR]	Δ Median	*p*-value[a]
Other desserts	66	86 [64]	23	100 [93]	14	0.39	87	140 [51]	21	100 [78]	-40	0.12	153	140 [58]	44	100 [85]	-40	0.082
Plain sweet biscuits	171	16 [12]	101	18 [17]	2	0.0032	187	16 [14]	93	18 [21]	2	0.072	358	16 [14]	194	18 [19]	2	0.0008
Cream/chocolate biscuits	80	19 [11]	34	22 [14]	3	0.89	102	18 [10]	30	21 [18]	3	0.17	182	19 [10]	64	21 [17]	2	0.28
Savoury biscuits	330	13 [11]	132	12 [10]	-1	0.035	332	13 [12]	114	13 [12]	0	0.69	662	13 [12]	246	12 [10]	-1	0.077
Muesli bars	57	31 [12]	34	31 [7]	0	0.62	62	31 [11]	35	31 [4]	0	0.17	119	31 [11]	69	31 [3]	0	0.24
**Main Meals**																		
Mince Dish	131	70 [70]	39	156 [156]	86	0.0000	132	68 [92]	45	93 [131]	25	0.016	263	70 [85]	84	134 [145]	64	0.0000
Plain Meat	122	46 [41]	32	93 [108]	47	0.0005	125	47 [49]	30	92 [58]	45	0.0005	247	46 [50]	62	92 [104]	46	0.0000
Crumbed Chicken	73	56 [51]	42	72 [64]	16	0.077	50	62 [56]	40	65 [69]	3	0.22	123	56 [54]	82	72 [66]	16	0.031
Plain Chicken	121	49 [52]	54	96 [78]	47	0.027	134	60 [53]	46	69 [80]	9	0.19	255	57 [51]	100	74 [78]	17	0.0092
Fish	63	69 [61]	26	67 [40]	-2	0.40	60	71 [54]	28	95 [80]	24	0.45	123	71 [59]	54	67 [85]	-4	0.94
Soup	50	193 [172]	39	159 [153]	-34	0.23	57	229 [183]	20	278 [190]	49	0.25	107	195 [175]	59	200 [227]	5	0.76
Sausages	96	41 [30]	41	89 [51]	48	0.0000	110	44 [39]	35	89 [39]	45	0.0000	206	41 [28]	76	89 [44]	48	0.0000
Pizza	29	70 [74]	22	100 [113]	30	0.0099	35	95 [82]	22	138 [106]	43	0.074	64	79 [96]	44	117 [106]	38	0.0026
Pie	32	16 [65]	20	84 [110]	68	0.46	58	98 [84]	19	95 [89]	-3	0.95	90	97 [76]	39	88 [115]	-9	0.63
**Other Foods**																		
Chips (not potato)	72	20 [15]	36	16 [24]	-4	0.75	80	18 [12]	33	19 [37]	1	0.72	152	18 [13]	69	19 [25]	1	0.99
Potato crisps	77	20 [7]	36	21 [15]	1	0.88	71	21 [5]	35	20 [14]	-1	0.047	148	21 [6]	71	20 [14]	-1	0.14
Ice block - water	30	70 [41]	26	75 [0]	5	0.19	38	75 [6]	20	75 [14]	0	0.59	68	73 [27]	46	75 [0]	2	0.60
Chocolate	152	15 [10]	68	15 [17]	0	0.46	176	14 [12]	45	15 [13]	1	0.27	328	14 [10]	113	15 [16]	1	0.22
Lollies	135	12 [12]	47	15 [19]	3	0.69	122	12 [12]	53	12 [11]	0	0.20	257	12 [12]	100	12 [14]	0	0.51
Nuts	38	13 [18]	17	10 [17]	-3	0.30	31	13 [9]	21	10 [16]	-3	0.26	69	13 [11]	38	10 [20]	-3	0.40
Jam/Honey	211	7 [7]	91	7 [4]	0	0.0067	260	7 [7]	87	8 [7]	1	0.072	471	7 [7]	178	7 [6]	0	0.0030

Table S3a: portion size (grams) changes between ANCNPAS 2007 and the NNPAS 2011–12, children 2-4 years old

	Females						Males						Persons					
	2007		2011–12				2007		2011–12				2007		2011–12			
**Food**	*n*	Median [IQR]	*n*	Median [IQR]	Δ Median	*p*-value[a]	*n*	Median [IQR]	*n*	Median [IQR]	Δ Median	*p*-value[a]	*n*	Median [IQR]	*n*	Median [IQR]	Δ Median	*p*-value[a]
Peanut butter	110	7 [7]	46	8 [5]	1	0.15	127	8 [8]	60	10 [8.6]	2	0.022	237	8 [8]	106	10 [4]	2	0.41
Vegemite	270	4 [2]	88	6 [3]	2	0.30	331	4 [3]	106	6 [3]	2	0.90	601	4 [3]	194	6 [3]	2	0.45
Tomato Sauce	147	6 [16]	41	14 [12]	8	0.017	186	7 [16]	53	14 [9]	7	0.33	333	6 [16]	94	14 [16]	8	0.022
Bacon/Ham	145	21 [25]	54	17 [9]	-4	0.0002	161	25 [20]	50	17 [8]	-8	0.0011	306	24 [23]	104	17 [5]	-7	0.0000
Eggs	108	43 [38]	50	43 [33]	0	0.18	106	42 [41]	36	47 [30]	5	0.14	214	43 [40]	86	44 [31]	1	0.043
**Fruit and Vegetables**																		
Hot Chips	118	75 [60]	64	47 [44]	-28	0.0004	120	75 [55]	48	29 [47]	-46	0.0000	238	75 [60]	112	36 [49]	-39	0.0001
Potato	187	56 [58]	59	59 [78]	3	0.99	209	61 [70]	49	70 [73]	9	0.63	396	61 [57]	108	60 [78]	-1	0.79
Peas	113	12 [28]	27	30 [28]	18	0.027	140	16 [15]	19	26 [45]	10	0.19	253	14 [24]	46	26 [35]	12	0.01
Broccoli	116	22 [22]	16	19 [10]	-3	0.48	107	20 [23]	23	19 [17]	-1	0.36	223	20 [22]	39	19 [16]	-1	0.87
Carrots	255	18 [22]	58	32 [28]	14	0.0002	298	17 [23]	55	30 [50]	13	0.0001	553	17 [22]	113	32 [44]	5	0.0003
Corn	106	23 [49]	27	70 [116]	47	0.0086	119	29 [30]	23	28 [68]	-1	0.48	225	27 [30]	50	49 [74]	22	0.013
Tomato	135	34 [44]	46	23 [55]	-31	0.049	131	45 [37]	26	29 [18]	-16	0.012	266	40 [41]	72	29 [38]	-11	0.0028
Celery/Cucumber	86	20 [34]	22	17 [29]	-3	0.42	81	20 [23]	23	40 [81]	20	0.0051	167	20 [23]	45	17 [58]	-3	0.14
Dried Fruit	132	20 [30]	35	21 [25]	1	0.50	167	19 [30]	48	16 [32]	-3	0.75	299	20 [30]	83	20 [29]	0	0.44
Apple	453	83 [100]	177	123 [73]	40	0.0000	509	87 [92]	166	139 [82]	52	0.0001	962	83 [95]	343	139 [78]	56	0.0000
Orange	186	66 [52]	56	75 [56]	9	0.0000	208	86 [49]	50	75 [56]	-11	0.0018	394	72 [53]	106	75 [56]	3	0.0000
Banana	319	94 [74]	98	98 [24]	4	0.43	302	94 [61]	119	98 [24]	4	0.58	621	94 [6]0	217	98 [24]	4	0.31
**Seasonal Fruit**																		
Mixed Berries/Grapes	187	50 [55]	105	50 [52]	0	0.44	177	56 [52]	106	60 [57]	4	0.99	364	50 [50]	211	53 [52]	3	0.58
Melon	84	75 [124]	35	75 [110]	0	0.92	106	111 [140]	36	123 [91]	12	0.88	190	87 [117]	71	85 [109]	-2	0.72

[a]*p*-values without Bonferroni correction

Table S3b: portion size changes (grams) between ANCNPAS 2007 and the NNPAS 2011–12, children 5–8 years old

	Females						Males						Persons					
	2007		2011–12				2007		2011–12				2007		2011–12			
Food	*n*	Median [IQR]	*n*	Median [IQR]	Δ Median	*p*-value[a]	*n*	Median [IQR]	*n*	Median [IQR]	Δ Median	*p*-value[a]	*n*	Median [IQR]	*n*	Median [IQR]	Δ Median	*p*-value[a]
**Drinks**																		
Soft Drink	117	234 [148]	75	208 [156]	-26	0.35	129	293 [190]	48	255 [174]	-38	0.10	246	261 [171]	123	250 [209]	-11	0.023
Water	1087	230 [195]	393	350 [550]	120	0.0000	1076	250 [250]	413	360 [800]	110	0.0000	2163	250 [245]	806	350 [800]	100	0.0000
Fruit Juice	282	210 [131]	142	208 [113]	-2	0.24	345	241 [131]	149	210 [107]	-31	0.49	627	210 [131]	291	210 [116]	0	0.24
**Dairy Foods**																		
Whole Milk	376	194 [129]	227	193 [165]	-1	0.027	418	206 [129]	240	206 [155]	0	0.0000	794	206 [129]	467	206 [155]	0	0.0000
Reduced Fat Milk	125	156 [108]	53	146 [114]	-10	0.72	147	194 [129]	65	208 [130]	14	0.069	272	170 [129]	118	156 [156]	-14	0.28
Flavoured Milk	81	226 [72]	29	212 [193]	-14	0.86	63	256 [124]	34	265 [99]	9	0.041	144	250 [93]	63	265 [160]	15	0.092
Ice cream	123	56 [81]	39	78 [56]	22	0.062	114	62 [73]	54	80 [83]	18	0.021	237	60 [75]	93	78 [77]	18	0.0019
Yoghurt	107	100 [80]	61	100 [92]	0	0.41	121	100 [80]	65	100 [80]	0	0.16	228	100 [80]	126	100 [88]	0	0.12
Cheese	223	21 [20]	104	25 [7]	4	0.28	259	21 [11]	111	21 [11]	0	0.70	482	21 [16]	215	21 [11]	0	0.57
**Breads and Cereals**																		
Hot Porridge	29	130 [174]	17	202 [208]	72	0.38	17	130 [220]	22	202 [260]	72	0.12	46	130 [200]	39	202 [260]	72	0.086
Breakfast Cereal	257	30 [21]	139	30 [15]	0	0.31	299	33 [18]	169	34 [21]	1	0.22	556	30 [15]	308	34 [17]	4	0.13
White Bread	399	56 [27]	219	56 [30]	0	0.26	394	62 [18]	228	64 [29]	2	0.18	793	60 [27]	447	64 [29]	4	0.066
Mixed Grain Bread	67	62 [34]	33	70 [12]	8	0.050	52	64 [31]	32	72 [44]	8	0.39	119	64 [28]	65	72 [27]	8	0.041
Wholemeal Bread	115	57 [24]	63	58 [34]	1	0.96	151	60 [26]	76	66 [10]	6	0.0034	266	60 [25]	139	66 [24]	6	0.026
Rice	73	111 [101]	56	119 [144]	8	0.31	64	162 [158]	53	114 [84]	-48	0.011	137	144 [114]	109	114 [108]	-30	0.016
Pasta	102	150 [115]	59	207 [223]	57	0.0050	128	150 [150]	49	230 [240]	80	0.0000	230	150 [133]	108	215 [245]	65	0.0000
**Sweets and Snacks**																		
Cakes	177	53 [51]	84	63 [88]	10	0.10	145	53 [50]	83	76 [92]	23	0.011	322	53 [50]	167	69 [94]	16	0.0034

Table S3b: portion size changes (grams) between ANCNPAS 2007 and the NNPAS 2011–12, children 5–8 years old

	Females						Males						Persons					
	2007		2011–12				2007		2011–12				2007		2011–12			
Food	*n*	Median [IQR]	*n*	Median [IQR]	Δ Median	*p*-value[a]	*n*	Median [IQR]	*n*	Median [IQR]	Δ Median	*p*-value[a]	*n*	Median [IQR]	*n*	Median [IQR]	Δ Median	*p*-value[a]
Sweet pie/pastry	27	50 [43]	19	67 [30]	17	0.10	30	60 [29]	28	67 [24]	7	0.41	57	60 [36]	47	67 [24]	7	0.11
Other desserts	42	145 [92]	20	100 [91]	-45	0.18	41	150 [50]	15	100 [70]	-50	0.091	83	150 [50]	35	100 [88]	-50	0.028
Plain sweet biscuits	102	22 [12]	80	18 [18]	-4	0.34	103	24 [19]	77	25 [18]	1	0.14	205	22 [15]	157	20 [17]	-2	0.093
Cream/chocolate biscuits	76	20 [13]	31	18 [15]	-2	0.090	87	21 [19]	40	23 [13]	2	0.75	163	20 [15]	71	20 [13]	0	0.19
Savoury biscuits	183	18 [15]	101	17 [14]	-1	0.60	174	20 [13]	101	18 [18]	-2	0.23	357	18 [13]	202	18 [16]	0	0.60
Savoury combi snack	27	25 [0]	16	24 [13]	-1	0.014	27	25 [0]	15	24 [1]	-1	0.0025	54	25 [0]	31	24 [1]	-1	0.0001
Muesli bars	59	31 [13]	38	31 [9]	0	0.52	66	31 [11]	56	31 [9]	0	0.73	125	31 [11]	94	31 [9]	0	0.55
**Main Meals**																		
Mince Dish	67	86 [100]	26	150 [272]	64	0.0073	96	94 [127]	27	156 [246]	62	0.043	163	89 [117]	53	150 [246]	61	0.0011
Plain Meat	69	56 [50]	24	105 [68]	50	0.0083	81	67 [59]	25	104 [81]	37	0.0002	150	65 [53]	49	104 [82]	39	0.0000
Crumbed Chicken	59	66 [36]	40	66 [74]	0	0.93	56	82 [62]	27	75 [68]	-7	0.66	115	72 [54]	67	72 [77]	0	0.65
Plain Chicken	86	67 [71]	34	81 [80]	14	0.16	87	76 [85]	27	105 [83]	29	0.42	173	72 [80]	61	100 [78]	28	0.13
Fish	42	73 [65]	25	71 [64]	-2	0.70	29	71 [61]	19	67 [55]	-4	0.26	71	71 [65]	44	69 [59]	-2	0.30
Soup	36	300 [259]	16	289 [394]	-11	0.47	30	253 [237]	18	306 [179]	53	0.16	66	259 [256]	34	303 [309]	44	0.18
Sausages	65	57 [30]	30	89 [43]	32	0.0002	78	58 [38]	39	89 [102]	31	0.0000	143	58 [39]	69	89 [101]	31	0.0000
Pizza	37	116 [87]	20	155 [94]	39	0.054	31	130 [134]	23	188 [147]	58	0.029	68	123 [101]	43	185 [122]	62	0.0027
Pie	56	124 [96]	19	130 [72]	6	0.39	44	140 [58]	22	131 [61]	-9	0.50	100	130 [64]	41	130 [61]	0	0.73
**Other Foods**																		
Chips (not potato)	52	20 [23]	41	25 [21]	5	0.94	66	25 [25]	47	22 [26]	-3	0.77	118	25 [24]	88	25 [21]	0	0.84
Potato crisps	96	21 [1]	52	21 [17]	0	0.79	106	21 [30]	47	21 [7]	0	0.053	202	21 [7]	99	21 [9]	0	0.12
Ice block - cream	23	59 [23]	19	80 [9]	21	0.004	23	64[20]	25	80 [9]	16	0.0010	46	64 [20]	44	80 [9]	16	0.0000
Ice block - water	42	74 [7]	37	75 [0]	1	0.56	36	75 [8]	33	75 [0]	0	0.13	78	75 [6]	70	75 [0]	0	0.14

Table S3b: portion size changes (grams) between ANCNPAS 2007 and the NNPAS 2011–12, children 5–8 years old

	Females						Males						Persons					
	2007		2011–12				2007		2011–12				2007		2011–12			
Food	*n*	Median [IQR]	*n*	Median [IQR]	Δ Median	*p*-value[a]	*n*	Median [IQR]	*n*	Median [IQR]	Δ Median	*p*-value[a]	*n*	Median [IQR]	*n*	Median [IQR]	Δ Median	*p*-value[a]
Chocolate	166	20 [23]	66	19 [25]	-1	0.75	159	20 [28]	62	19 [15]	-1	0.71	325	20 [25]	128	19 [22]	-1	0.65
Lollies	131	14 [16]	57	15 [13]	1	0.83	127	16 [14]	60	15 [20]	-1	0.70	258	15 [16]	117	15 [17]	0	0.92
Salad dressing full fat	22	5 [13]	11	21 [7]	16	0.0019	26	9 [15]	23	14 [14]	5	0.080	48	6 [14]	34	14 [7]	8	0.0009
Jam/Honey	124	12 [7]	71	8 [7]	-4	0.79	122	8 [7]	81	8 [7]	0	0.46	246	11 [7]	152	8 [7]	-3	0.45
Peanut butter	84	10 [13]	36	9 [4]	-1	0.055	96	10 [13]	41	9 [4]	-1	0.14	180	10 [13]	77	9 [4]	-1	0.015
Vegemite	157	5 [4]	70	6 [3]	1	0.17	140	5 [4]	67	6 [1]	1	0.32	297	5 [4]	137	6 [4]	1	0.76
Tomato Sauce	148	11 [16]	38	14 [7]	3	0.0049	145	13 [16]	52	14 [16]	1	0.046	293	11 [16]	90	14 [14]	3	0.0005
Bacon/Ham	107	34 [26]	59	17 [8]	-17	0.0000	128	29 [30]	62	17 [17]	-12	0.0000	235	30 [26]	121	17 [9]	-13	0.0000
Eggs	64	44 [36]	47	55 [36]	11	0.0056	76	43 [52]	35	47 [27]	4	0.27	140	43 [45]	82	53 [36]	10	0.0091
**Fruit and Vegetables**																		
Hot Chips	108	75 [40]	51	48 [67]	-27	0.0001	97	77 [38]	51	57 [57]	-20	0.0000	205	75 [40]	102	55 [67]	-20	0.0000
Potato	111	75 [73]	47	83 [97]	8	0.80	106	92 [78]	44	83 [103]	-9	0.84	217	75 [76]	91	83 [97]	8	0.94
Peas	61	20 [29]	15	26 [27]	6	0.63	56	22 [29]	15	35 [40]	13	0.26	117	20 [29]	0	30 [27]	10	0.25
Broccoli	63	26 [21]	15	47 [24]	21	0.0087	67	26 [33]	23	19 [53]	-7	0.41	130	26 [21]	38	35 [39]	9	0.34
Carrots	166	29 [28]	57	37 [49]	8	0.0004	160	30 [37]	52	44 [100]	14	0.0077	326	29 [33]	109	38 [54]	9	0.0000
Corn	57	24 [48]	12	61 [85]	37	0.022	48	35 [37]	24	75 [98]	40	0.0003	105	32 [44]	36	71 [98]	39	0.0000
Tomato	77	60 [46]	24	29 [19]	-31	0.0000	82	47 [50]	30	29 [22]	-18	0.010	159	51 [49]	54	29 [22]	-22	0.0000
Lettuce	59	8 [9]	14	12 [0]	4	0.21	72	11 [20]	20	16 [16]	5	0.068	131	9 [13]	34	13 [13]	4	0.022
Celery/Cucumber	71	25 [18]	23	31 [89]	6	0.20	73	20 [30]	16	30 [33]	10	0.15	144	24 [24]	39	31 [64]	7	0.048
Apple	238	156 [72]	148	151 [54]	-5	0.12	224	166 [58]	157	164 [25]	-2	0.21	462	161 [58]	305	158 [29]	-3	0.053
Orange	116	86 [45]	76	75 [56]	-11	0.27	98	86 [59]	49	75 [56]	-11	0.22	214	86 [45]	125	75 [56]	-11	0.10
Banana	125	121 [27]	58	98 [24]	-23	0.0000	129	121 [27]	73	98 [13]	-23	0.064	254	121 [27]	131	98 [0]	-23	0.0000
**Seasonal Fruit**																		

Table S3b: portion size changes (grams) between ANCNPAS 2007 and the NNPAS 2011-12, children 5-8 years old

	Females						Males						Persons					
	2007		2011–12				2007		2011–12				2007		2011–12			
**Food**	*n*	Median [IQR]	*n*	Median [IQR]	Δ Median	*p*-value[a]	*n*	Median [IQR]	*n*	Median [IQR]	Δ Median	*p*-value[a]	*n*	Median [IQR]	*n*	Median [IQR]	Δ Median	*p*-value[a]
Mixed Berries/Grapes	73	60 [71]	75	50 [57]	-10	0.030	63	56 [49]	73	60 [49]	4	0.82	136	60 [78]	148	53 [49]	-7	0.12
Melon	55	133 [113]	29	128 [85]	-5	0.90	48	150 [217]	30	138 [116]	-12	0.28	103	143 [151]	59	128 [95]	-15	0.41

[a]*p*-values without Bonferroni correction

Table S3c: portion size changes (grams) between ANCNPAS 2007 and the NNPAS 2011–12, children 9–12 years old

	Females						Males						Persons					
	2007		2011–12				2007		2011–12				2007		2011–12			
Food	*n*	Median [IQR]	*n*	Median [IQR]	Δ Median	*p*-value[a]	*n*	Median [IQR]	*n*	Median [IQR]	Δ Median	*p*-value[a]	*n*	Median [IQR]	*n*	Median [IQR]	Δ Median	*p*-value[a]
**Drinks**																		
Diet soft drink	32	278 [260]	17	290 [175]	12	0.71	31	300 [145]	24	290 [125]	-10	0.57	63	280 [175]	41	290 [175]	10	0.87
Soft Drink	194	263 [182]	89	302 [130]	39	0.74	209	245 [195]	100	364 [96]	119	0.16	403	304 [182]	189	312 [130]	8	0.52
Water	1259	250 [228]	444	350 [800]	100	0.0000	1110	250 [250]	477	350 [790]	100	0.0000	2369	250 [235]	921	350 [800]	100	0.0000
Fruit Juice	322	263 [105]	155	260 [74]	-3	0.0063	266	261 [52]	145	263 [154]	2	0.0025	588	241 [62]	300	260 [97]	19	0.0000
Tea or coffee	57	240 [34]	22	200 [20]	-40	0.0081	32	240 [62]	24	220 [50]	-20	0.86	89	240 [40]	46	220 [50]	-20	0.070
**Dairy Foods**																		
Whole Milk	322	232 [129]	151	206 [129]	-26	0.10	346	257 [257]	218	206 [129]	-51	0.0005	668	237 [129]	369	206 [129]	-31	0.0003
Reduced Fat Milk	174	194 [172]	87	208 [156]	14	0.023	177	237 [131]	80	208 [211]	-29	0.88	351	206 [129]	167	208 [156]	2	0.13
Flavoured Milk	88	258 [137]	32	352 [186]	94	0.0011	79	258 [124]	40	340 [203]	82	0.035	167	258 [115]	72	351 [199]	93	0.0001
Ice cream	124	100 [74]	58	89 [66]	-11	0.41	107	90 [174]	54	102 [96]	12	0.018	231	93 [74]	112	96 [81]	3	0.028
Yoghurt	78	100 [107]	49	123 [100]	23	0.39	77	100 [100]	42	136 [73]	36	0.081	155	100 [100]	91	123 [75]	23	0.063
Cheese	252	21 [15]	73	25 [10]	4	0.50	203	21 [21]	89	25 [18]	4	0.073	455	21 [20]	162	25 [12]	4	0.066
**Breads and Cereals**																		
Breakfast Cereal	199	41 [30]	111	36 [24]	-5	0.73	271	45 [36]	147	43 [36]	-2	0.31	470	45 [30]	258	39 [27]	-6	0.53
White Bread	400	62 [24]	226	64 [31]	2	0.49	402	64 [26]	268	64 [16]	0	0.16	802	64 [32]	494	64 [15]	0	0.10
Mixed Grain Bread	66	64 [25]	25	72 [12]	8	0.31	43	66 [29]	32	72 [31]	6	0.29	109	64 [27]	57	72 [27]	8	0.14
Wholemeal Bread	97	64 [28]	42	66 [10]	2	0.14	99	64 [16]	58	66 [20]	2	0.17	196	64 [24]	100	66 [10]	2	0.047
Rice	86	158 [121]	72	168 [123]	10	0.84	73	190 [165]	66	134 [134]	-56	0.0023	159	190 [125]	138	141 [142]	-49	0.021
Pasta	112	150 [113]	58	240 [391]	90	0.0030	106	197 [170]	75	312 [276]	115	0.0000	218	184 [170]	133	312 [312]	128	0.0000
**Sweets and Snacks**																		
Cakes	172	61 [65]	82	102 [113]	41	0.0006	140	71[82]	81	94 [115]	23	0.081	312	62 [71]	163	95 [115]	33	0.0002

Table S3c: portion size changes (grams) between ANCNPAS 2007 and the NNPAS 2011–12, children 9–12 years old

	Females						Males						Persons					
	2007		2011–12				2007		2011–12				2007		2011–12			
Food	*n*	Median [IQR]	*n*	Median [IQR]	Δ Median	*p*-value[a]	*n*	Median [IQR]	*n*	Median [IQR]	Δ Median	*p*-value[a]	*n*	Median [IQR]	*n*	Median [IQR]	Δ Median	*p*-value[a]
Sweet pie/pastry	52	63 [57]	21	85 [68]	22	0.014	42	80 [74]	28	67 [40]	-13	0.54	94	75 [64]	49	67 [60]	-8	0.20
Other desserts	49	137 [60]	25	95 [110]	-42	0.043	35	113 [50]	16	100 [89]	-13	0.12	84	128 [54]	41	100 [90]	-28	0.0078
Plain sweet biscuits	104	20 [17]	74	23 [18]	3	0.032	75	25 [22]	71	30 [16]	5	0.40	179	22 [18]	145	25 [17]	3	0.012
Cream/chocolate biscuits	77	25 [16]	39	24 [23]	-1	0.53	86	25 [18]	42	25 [15]	0	0.93	163	25 [17]	81	25 [17]	0	0.65
Savoury biscuits	169	18 [13]	83	25 [22]	7	0.0025	127	24 [12]	81	25 [23]	1	0.23	296	20 [13]	164	25 [23]	5	0.0017
Snack noodles	35	131 [94]	17	145 [240]	14	0.0024	38	136 [125]	18	263 [240]	127	0.0046	73	136 [84]	35	204 [240]	68	0.0000
Muesli bars	71	31 [11]	55	31 [0]	0	0.50	72	31 [11]	59	31 [5]	0	0.18	143	31 [11]	114	31 [1]	0	0.15
**Main Meals**																		
Mince Dish	80	140 [148]	41	192 [349]	52	0.13	83	128 [135]	43	312 [372]	184	0.0000	163	138 [140]	84	275 [374]	137	0.0000
Mixed meat dish	14	126 [316]	20	152 [93]	26	0.94	8	210 [232]	24	272 [127]	62	0.60	22	154 [284]	44	204 [208]	50	0.85
Plain Meat	88	86 [86]	32	118 [74]	32	0.0058	95	84 [88]	33	127 [99]	43	0.0007	183	84 [85]	65	123 [70]	39	0.0000
Mixed chicken dish	30	100 [157]	27	215 [214]	115	0.59	24	127 [120]	25	258 [137]	131	0.0011	54	107 [157]	52	226 [187]	119	0.0054
Crumbed Chicken	61	72 [65]	35	96 [74]	24	0.11	43	109 [72]	32	134 [96]	25	0.038	104	90 [81]	67	114 [92]	24	0.0081
Plain Chicken	99	78 [78]	34	75 [45]	-3	0.30	95	95 [85]	20	168 [112]	73	0.0069	194	84 [90]	54	105 [115]	21	0.035
Fish	29	95 [76]	21	83 [53]	-12	0.89	27	115 [131]	17	124 [126]	9	0.28	56	101 [74]	38	110 [78]	9	0.64
Soup	45	300 [166]	25	340 [303]	40	0.56	31	260 [338]	18	326 [206]	66	0.36	76	287 [273]	43	333 [308]	46	0.33
Sausages	68	58 [73]	24	108 [89]	50	0.0000	71	72 [55]	33	94 [89]	22	0.0005	139	58 [67]	57	101 [89]	43	0.0000
Hamburger	22	158 [91]	23	185 [125]	27	0.25	32	193 [92]	21	185 [53]	-8	0.57	54	186 [97]	44	185 [112]	-1	0.29
Pizza	48	110 [97]	19	204 [193]	94	0.0029	37	164 [155]	24	204 [108]	40	0.19	85	132 [134]	43	204 [168]	72	0.0006
Pie	54	140 [65]	20	84 [132]	-56	0.14	64	170 [49]	35	130 [95]	-40	0.21	118	165 [57]	55	130 [99]	-35	0.069
**Other Foods**																		
Chips (not potato)	61	22 [30]	46	23 [10]	1	0.07	60	20 [30]	45	22 [23]	2	0.82	121	21 [32]	91	22 [13]	1	0.16

Table S3c: portion size changes (grams) between ANCNPAS 2007 and the NNPAS 2011–12, children 9–12 years old

	Females						Males						Persons					
	2007		2011–12				2007		2011–12				2007		2011–12			
**Food**	*n*	Median [IQR]	*n*	Median [IQR]	Δ Median	*p*-value[a]	*n*	Median [IQR]	*n*	Median [IQR]	Δ Median	*p*-value[a]	*n*	Median [IQR]	*n*	Median [IQR]	Δ Median	*p*-value[a]
Potato crisps	107	21 [9]	60	21 [25]	0	0.31	119	21 [21]	70	21 [5]	0	0.019	226	21 [14]	130	21 [20]	0	0.31
Ice block - cream	29	65 [8]	21	80 [5]	15	0.0003	30	68 [36]	23	80 [9]	12	0.18	59	68 [50]	44	80 [8]	12	0.0007
Ice block - water	35	75 [8]	35	75 [0]	0	0.78	33	76 [10]	30	75 [0]	-1	0.52	68	76 [6]	65	75 [0]	-1	0.79
Chocolate	182	18 [24]	79	24 [37]	6	0.24	171	20 [31]	74	25 [32]	5	0.59	353	20 [27]	153	25 [35]	5	0.20
Lollies	139	16 [23]	60	15 [16]	-1	0.30	131	16 [25]	58	15 [18]	-1	0.78	270	16 [23]	118	15 [15]	9	0.35
Salad dressing full fat	43	9 [15]	28	17 [8]	8	0.0003	40	11 [15]	28	14 [7]	3	0.0088	83	10 [14]	56	14 [7]	4	0.0000
Jam/Honey	104	12 [14]	37	14 [19]	2	0.25	89	14 [17]	45	11 [19]	-3	0.68	193	13 [16]	82	12 [19]	-1	0.28
Peanut butter	76	10 [13]	32	9 [2]	-1	0.15	86	12 [11]	43	13 [16]	1	0.57	12	12 [13]	75	10 [9]	-2	0.63
Vegemite	140	5 [4]	59	6 [4]	1	0.021	93	5 [3]	84	6 [4]	1	0.89	233	5 [4]	143	6 [4]	1	0.17
Tomato Sauce	160	13 [16]	52	14 [7]	1	0.10	166	11 [16]	63	17 [14]	6	0.0062	326	13 [16]	115	14 [9]	1	0.0012
Devon, salami	31	25 [32]	15	45 [22]	20	0.32	38	25 [12]	19	28 [33]	3	0.044	69	25 [21]	34	28 [23]	3	0.025
Bacon/Ham	107	30 [30]	69	17 [15]	-13	0.0001	99	30 [25]	63	17 [17]	-13	0.0043	206	30 [26]	132	17 [17]	-13	0.0000
Eggs	60	43 [39]	39	49 [28]	6	0.17	67	50 [41]	30	51 [44]	1	0.11	127	49 [41]	69	49 [42]	0	0.049
**Fruit and Vegetables**																		
Hot Chips	114	100 [75]	61	70 [50]	-30	0.0000	103	110 [75]	56	93 [98]	-17	0.032	217	104 [75]	117	72 [64]	-32	0.0000
Potato	131	107 [73]	45	111 [116]	4	0.72	118	110 [118]	57	123 [103]	13	0.57	249	110 [84]	102	122 [116]	12	0.39
Broccoli	73	30 [24]	14	62 [88]	32	0.0027	57	30 [31]	17	29 [28]	-1	0.48	130	30 [30]	31	38 [58]	8	0.16
Carrots	183	29 [26]	46	35 [92]	6	0.042	145	29 [29]	44	35 [63]	6	0.0083	328	29 [27]	90	35 [82]	6	0.0011
Tomato	129	60 [40]	44	29 [9]	-31	0.0000	97	60 [40]	30	30 [39]	-30	0.039	226	60 [40]	74	29 [22]	-31	0.0000
Lettuce	106	15 [21]	37	12 [0]	-3	0.11	74	16 [21]	30	13 [12]	-3	0.65	180	15 [21]	67	12 [12]	-3	0.13
Celery/Cucumber	82	25 [22]	31	30 [89]	5	0.039	57	25 [20]	17	30 [89]	5	0.054	139	25 [21]	48	30 [89]	5	0.0042
Apple	244	166 [58]	115	164 [20]	-2	0.69	178	166 [57]	161	164 [20]	-2	0.40	422	166 [58]	276	164 [20]	-2	0.89
Orange	104	86 [59]	68	75 [63]	-11	0.77	77	86 [52]	53	101 [56]	15	0.46	181	86 [59]	121	75 [56]	-11	0.48

Table S3c: portion size changes (grams) between ANCNPAS 2007 and the NNPAS 2011–12, children 9–12 years old

	Females						Males						Persons					
	2007		2011–12				2007		2011–12				2007		2011–12			
Food	*n*	Median [IQR]	*n*	Median [IQR]	Δ Median	*p*-value[a]	*n*	Median [IQR]	*n*	Median [IQR]	Δ Median	*p*-value[a]	*n*	Median [IQR]	*n*	Median [IQR]	Δ Median	*p*-value[a]
Banana	105	121 [27]	50	98 [13]	-23	0.0046	91	121 [27]	43	98 [13]	-23	0.0044	196	121 [27]	93	98 [13]	-23	0.0001
**Seasonal Fruit**																		
Peach, nectarin, plum or apricot	16	87 [85]	32	151 [6]	64	0.014	12	132 [82]	19	145 [76]	13	0.57	28	116 [85]	51	151 [19]	35	0.032
Mixed Berries/Grapes	58	83 [91]	51	60 [100]	-23	0.46	44	84 [94]	40	43 [83]	-41	0.0091	102	84 [85]	91	50 [74]	-34	0.020
Melon	33	161 [174]	26	150 [144]	-11	0.82	28	177 [241]	13	300 [170]	123	0.49	61	161 [211]	39	160 [219]	-1	0.89

[a]*p*-values without Bonferroni correction

Table S3d: portion size changes (grams) between ANCNPAS 2007 and the NNPAS 2011–12, children 13–16 years old

	Females						Males						Persons					
	2007		2011–12				2007		2011–12				2007		2011–12			
Food	*n*	Median [IQR]	*n*	Median [IQR]	Δ Median	*p*-value[a]	*n*	Median [IQR]	*n*	Median [IQR]	Δ Median	*p*-value[a]	*n*	Median [IQR]	*n*	Median [IQR]	Δ Median	*p*-value[a]
**Drinks**																		
Diet soft drink	61	375 [138]	24	375 [105]	0	0.85	84	375 [75]	28	375 [0]	0	0.21	145	375 [125]	52	375 [23]	0	0.53
Soft Drink	301	340 [178]	133	364 [130]	24	0.78	397	391 [187]	194	390 [234]	-1	0.92	698	391 [156]	327	390 [208]	-1	0.94
Water	1594	300 [335]	458	450 [710]	150	0.0000	1564	300 [370]	444	500 [1020]	200	0.0000	3158	300 [380]	902	500 [710]	200	0.0000
Fruit Juice	425	262 [52]	143	263 [158]	1	0.0000	401	262 [81]	150	272 [118]	10	0.0039	826	262 [105]	293	270 [108]	8	0.0000
Tea or coffee	193	240 [28]	91	220 [50]	-20	0.14	159	260 [54]	56	220 [66]	-40	0.0000	352	240 [37]	147	220 [50]	-20	0.0000
**Dairy Foods**																		
Whole Milk	357	206 [176]	142	206 [227]	0	0.86	553	258 [214]	215	258 [211]	0	0.012	910	258 [194]	357	258 [237]	0	0.043
Reduced Fat Milk	196	175 [196]	84	195 [177]	20	0.10	216	258 [231]	73	208 [208]	-50	0.068	412	237 [216]	157	208 [177]	-29	0.77
Flavoured Milk	138	278 [142]	49	432 [265]	154	0.0002	130	353 [267]	56	346 [274]	-7	0.68	268	307 [242]	105	386 [265]	79	0.0027
Ice cream	107	112 [112]	49	97 [95]	-15	0.60	136	112 [106]	54	132 [120]	20	0.030	243	112 [108]	103	132 [111]	20	0.25
Yoghurt	108	150 [114]	43	130 [138]	-20	0.23	79	200 [159]	32	113 [86]	-87	0.055	187	156 [100]	75	123 [105]	-33	0.034
Cheese	330	22 [25]	52	25 [6]	3	0.54	297	33 [25]	86	25 [21]	-8	0.24	627	23 [25]	138	25 [12]	2	0.48
**Breads and Cereals**																		
Breakfast Cereal	214	45 [38]	91	45 [23]	0	0.35	385	60 [45]	151	51 [50]	-9	0.0044	599	60 [40]	242	51 [38]	-9	0.0015
White Bread	468	64 [34]	173	64 [16]	0	0.86	582	66 [20]	255	64 [22]	-2	0.018	1050	64 [21]	428	64 [18]	0	0.094
Mixed Grain Bread	110	68 [29]	33	60 [12]	-8	0.41	84	68 [28]	24	72 [12]	4	0.72	194	68 [22]	57	72 [12]	4	0.66
Wholemeal Bread	132	64 [31]	59	64 [13]	0	0.85	114	68 [16]	33	66 [10]	-2	0.99	246	64 [26]	92	66 [13]	2	0.62
Rice	117	190 [186]	60	168 [121]	-22	0.04	125	247 [237]	61	201 [256]	-46	0.17	242	216 [224]	121	184 [111]	-32	0.013
Pasta	151	210 [168]	74	298 [266]	88	0.0004	162	253 [182]	68	312 [208]	59	0.0000	313	225 [152]	142	312 [239]	87	0.0000

Table S3d: portion size changes (grams) between ANCNPAS 2007 and the NNPAS 2011–12, children 13–16 years old

	Females						Males						Persons					
	2007		2011–12				2007		2011–12				2007		2011–12			
Food	*n*	Median [IQR]	*n*	Median [IQR]	Δ Median	*p*-value[a]	*n*	Median [IQR]	*n*	Median [IQR]	Δ Median	*p*-value[a]	*n*	Median [IQR]	*n*	Median [IQR]	Δ Median	*p*-value[a]
**Sweets and Snacks**																		
Cakes	210	72 [80]	92	85 [82]	13	0.25	193	80 [81]	64	132 [90]	52	0.0001	403	76 [80]	156	110 [103]	34	0.0006
Sweet pie/pastry	50	67 [61]	27	80 [39]	13	0.64	53	80 [70]	34	67 [46]	13	0.21	103	75 [72]	61	76 [47]	1	0.58
Other desserts	48	115 [105]	18	129 [80]	14	0.97	37	150 [130]	18	121 [125]	-29	0.30	85	140 [105]	36	121 [5]	-19	0.51
Plain sweet biscuits	104	22 [22]	49	26 [19]	4	0.25	106	28 [24]	50	34 [34]	6	0.95	210	24 [24]	99	30 [29]	6	0.45
Cream/chocolate biscuits	89	23 [22]	36	22 [32]	-1	0.85	116	28 [21]	37	28 [32]	0	0.80	205	25 [21]	73	25 [32]	0	0.95
Savoury biscuits	154	24 [20]	77	25 [35]	1	0.50	127	25 [23]	65	28 [55]	3	0.19	281	25 [20]	142	25 [48]	0	0.18
Snack noodles	54	169 [125]	17	145 [240]	-24	0.27	49	170 [125]	24	166 [145]	-4	0.49	103	169 [125]	41	145 [240]	-24	0.20
Muesli bars	112	31 [10]	53	31 [3]	0	0.11	123	31 [11]	65	31 [2]	0	0.68	235	31 [11]	118	31 [2]	0	0.18
**Main Meals**																		
Mince Dish	97	92 [142]	51	218 [270]	126	0.0000	121	170 [175]	46	280 [320]	110	0.034	218	140 [180]	97	260 [286]	120	0.0000
Mixed meat dish	24	190 [163]	20	237 [171]	47	0.16	20	253 [316]	19	243 [358]	-10	0.93	44	243 [167]	39	243 [231]	0	0.31
Plain Meat	141	80 [78]	54	142 [87]	62	0.0000	159	104 [98]	55	183 [84]	79	0.0000	300	88 [84]	109	150 [94]	62	0.0000
Crumbed Chicken	61	99 [89]	32	109 [104]	10	0.86	77	143 [135]	37	130 [56]	-13	0.84	138	117 [130]	69	114 [87]	-3	0.91
Plain Chicken	130	101 [86]	45	114 [93]	13	0.060	163	120 [108]	39	114 [128]	-6	0.76	293	107 [100]	84	114 [105]	7	0.17
Sausages	59	58 [48]	25	89 [28]	31	0.077	98	82 [57]	31	156 [89]	74	0.0000	157	64 [57]	56	101 [89]	37	0.0000
Hamburger	46	191 [113]	37	185 [63]	-6	0.78	42	203 [124]	60	206 [113]	3	0.087	88	201 [123]	97	196 [118]	-5	0.072
Pizza	56	142 [166]	29	175 [175]	33	0.13	87	225 [184]	41	188 [264]	-37	0.40	143	174 [193]	70	186 [256]	12	0.98
Pie	75	140 [64]	27	175 [45]	35	0.019	106	174 [60]	48	175 [45]	1	0.83	181	170 [60]	75	175 [45]	5	0.15
**Other Foods**																		
Chips (not potato)	75	28 [30]	35	20 [28]	-8	0.012	58	50 [40]	34	25 [49]	-25	0.16	133	30 [36]	69	25 [29]	-5	0.008

Table S3d: portion size changes (grams) between ANCNPAS 2007 and the NNPAS 2011–12, children 13–16 years old

	Females						Males						Persons					
	2007		2011–12				2007		2011–12				2007		2011–12			
Food	*n*	Median [IQR]	*n*	Median [IQR]	Δ Median	*p*-value[a]	*n*	Median [IQR]	*n*	Median [IQR]	Δ Median	*p*-value[a]	*n*	Median [IQR]	*n*	Median [IQR]	Δ Median	*p*-value[a]
Potato crisps	145	21 [29]	17	78 [4]	57	0.47	147	21 [29]	61	42 [26]	21	0.99	292	21 [29]	106	21 [26]	0	0.70
Chocolate	237	24 [29]	77	25 [40]	1	0.55	177	30 [37]	68	31 [38]	1	0.98	414	26 [34]	145	25 [40]	-1	0.55
Lollies	176	15 [22]	62	15 [19]	0	0.71	149	28 {29]	39	21 [26]	-7	0.05	325	18 [30]	101	17 [22]	-1	0.19
Salad dressing full fat	74	10 [15]	27	21 [8]	11	0.0018	84	10 [15]	42	14 [7]	4	0.0014	158	10 [15]	69	20 [7]	10	0.0000
Jam/Honey	151	13 [19]	42	11 [21]	-2	0.69	130	14 [18]	52	17 [31]	3	0.071	281	13 [19]	94	14 [21]	1	0.21
Peanut butter	90	12 [13]	36	10 [11]	-2	0.95	115	19 [13]	44	10 [10]	-9	0.0096	205	13 [15]	80	10 [10]	-3	0.057
Vegemite	162	6 [4]	49	6 [4]	0	0.012	112	8 [5]	48	6 [6]	-2	0.38	274	6 [6]	97	6 [4]	0	0.035
Tomato Sauce	191	22 [17]	56	14 [28]	-8	0.086	253	22 [20]	86	14 [14]	-8	0.57	444	22 [18]	142	14 [19]	-8	0.12
Bacon/Ham	160	27 [22]	69	26 [31]	-1	0.90	175	40 [35]	82	25 [31]	-15	0.0001	335	36 [33]	151	25 [31]	-11	0.0061
Eggs	108	43 [54]	42	49 [47]	6	0.074	91	43 [49]	45	51 [34]	8	0.0060	199	43 [55]	87	51 [34]	8	0.0013
**Fruit and Vegetables**																		
Hot Chips	187	112 [75]	66	75 [70]	-37	0.0005	168	144 [41]	61	104 [71]	-40	0.0003	355	133 [51]	127	100 [71]	-33	0.0000
Potato	165	111 [100]	64	119 [99]	8	0.75	166	146 [124]	47	123 [155]	-23	0.50	331	122 [121]	111	122 [118]	0	0.49
Carrots	230	29 [30]	40	37 [61]	8	0.30	200	34 [25]	47	37 [77]	3	0.63	430	31 [30]	87	37 [63]	8	0.23
Tomato	147	60 [53]	43	29 [51]	-31	0.0000	154	63 [55]	36	37 [29]	-26	0.0001	301	60 [55]	79	30 [29]	-30	0.0000
Lettuce	138	16 [16]	31	13 [16]	-3	0.088	161	16 [20]	56	13 [13]	-3	0.10	299	16 [20]	87	13 [13]	-3	0.011
Celery/Cucumber	108	26 [28]	20	26 [22]	0	0.43	87	30 [20]	24	17 [11]	-13	0.024	195	29 [24]	44	23 [20]	-6	0.32
Onion	156	13 [15]	27	18 [25]	5	0.56	182	19 [23]	13	9 [5]	-10	0.0014	338	17 [18]	40	11 [16]	-6	0.072
Apple	245	166 [10]	84	164 [11]	-2	0.0021	205	166 [5]	106	164 [21]	-2	0.012	450	166 [5]	190	164 [15]	-2	0.0003
Orange	97	121 [27]	48	75 [56]	-46	0.39	116	121 [27]	42	75 [75]	-46	0.66	280	99 [45]	90	75 [56]	-24	0.35
Banana	158	86 [45]	36	98 [0]	12	0.0001	122	101 [45]	33	98 [13]	-3	0.0040	213	121 [27]	69	98 [0]	-23	0.0000
**Seasonal Fruit**																		

Table S3d: portion size changes (grams) between ANCNPAS 2007 and the NNPAS 2011–12, children 13–16 years old

	Females						Males						Persons					
	2007		2011–12				2007		2011–12				2007		2011–12			
**Food**	*n*	Median [IQR]	*n*	Median [IQR]	Δ Median	*p*-value[a]	*n*	Median [IQR]	*n*	Median [IQR]	Δ Median	*p*-value[a]	*n*	Median [IQR]	*n*	Median [IQR]	Δ Median	*p*-value[a]
Peach, nectarin, plum or apricot	24	66 [108]	26	151 [6]	85	0.0044	10	148 [129]	17	151 [76]	3	0.82	34	94 [85]	43	151 [13]	57	0.020
Mixed Berries/Grapes	69	82 [139]	36	72 [46]	-10	0.63	46	60 [100]	25	100 [134]	40	0.033	115	76 [139]	61	75 [72]	-1	0.27

[a]*p*-values without Bonferroni correction

Table S3e: portion size changes (grams) between ANCNPAS 2007 and the NNPAS 2011–12, children 2–16 years old

	Females						Males						Persons					
	2007		2011–12				2007		2011–12				2007		2011–12			
Food	*n*	Median [IQR]	*n*	Median [IQR]	Δ Median	*p*-value[a]	*n*	Median [IQR]	*n*	Median [IQR]	Δ Median	*p*-value[a]	*n*	Median [IQR]	*n*	Median [IQR]	Δ Median	*p*-value[a]
**Drinks**																		
Diet soft drink	137	250 [205]	49	350 [175]	100	0.17	155	320 [175]	62	368 [125]	48	0.35	292	291 [175]	111	360 [125]	69	0.11
Soft Drink	701	261 [196]	321	302 [182]	41	0.76	832	313 [161]	363	364 [134]	51	0.47	1533	304 [183]	684	348 [182]	44	0.82
Water	5626	250 [275]	1771	350 [550]	100	0.0000	5553	250 [275]	1815	350 [800]	100	0.0000	11179	250 [270]	3586	350 [800]	100	0.0000
Fruit Juice	1521	210 [131]	605	210 [117]	0	0.0000	1532	241 [131]	632	260 [147]	19	0.0000	3053	210 [131]	1237	260 [146]	50	0.0000
Tea or coffee	307	240 [74]	148	200 [50]	-40	0.088	260	240 [65]	99	220 [100]	-20	0.0012	567	240 [70]	247	220 [50]	-20	0.0004
**Dairy Foods**																		
Whole Milk	2089	194 [129]	941	206 [160]	12	0.0030	2500	206 [129]	1070	206 [155]	0	0.0000	4589	206 [129]	2011	206 [155]	0	0.0000
Reduced Fat Milk	714	155 [172]	304	165 [177]	10	0.037	761	206 [129]	277	208 [156]	2	0.32	1475	194 [145]	581	208 [161]	14	0.058
Flavoured Milk	375	258 [106]	136	340 [229]	82	0.0000	352	258 [163]	159	283 [192]	25	0.025	727	258 [147]	295	318 [216]	60	0.0000
Ice cream	480	62 [75]	190	78 [81]	16	0.025	466	70 [83]	208	88 [90]	18	0.0000	946	68 [78]	398	81 [77]	13	0.0000
Yoghurt	592	100 [80]	277	100 [80]	0	0.22	592	100 [80]	257	100 [75]	0	0.041	1184	100 [80]	534	100 [80]	0	0.022
Cheese	1245	21 [19]	367	21 [9]	0	0.12	1243	21 [20]	422	25 [9]	4	0.058	2488	21 [18]	789	24 [8]	3	0.011
**Breads and Cereals**																		
Hot porridge	138	130 [213]	89	180 [213]	50	0.11	120	211 [177]	63	202 [208]	-9	0.68	258	195 [200]	152	193 [213]	-2	0.20
Breakfast Cereal	1132	30 [23]	522	33 [25]	3	0.69	1476	38 [30]	651	34 [27]	-4	0.30	2608	33 [37]	1173	34 [30]	1	0.25
White Bread	1761	54 [32]	807	60 [31]	6	0.011	1967	62 [37]	936	64 [16]	2	0.0017	3728	60 [31]	1743	64 [32]	4	0.0000
Mixed Grain Bread	352	64 [34]	140	60 [36]	-4	0.43	332	64 [31]	157	70 [36]	6	0.042	684	64 [32]	297	60 [36]	-4	0.035
Wholemeal Bread	599	52[32]	266	62 [31]	10	0.0097	610	60 [31]	252	66 [24]	6	0.0084	1209	56 [30]	518	64 [30]	8	0.0002
Rice	404	143[134]	256	127 [139]	-16	0.023	384	175 [190]	259	134 [125]	-41	0.0002	788	144 [123]	515	134 [134]	-10	0.0000
Pasta	594	150 [150]	283	208 [223]	58	0.0000	643	150 [169]	265	310 [247]	160	0.0000	1237	150 [142]	548	220 [251]	70	0.0000

Table S3e: portion size changes (grams) between ANCNPAS 2007 and the NNPAS 2011–12, children 2–16 years old

	Females						Males						Persons					
	2007		2011–12				2007		2011–12				2007		2011–12			
Food	*n*	Median [IQR]	*n*	Median [IQR]	Δ Median	*p*-value[a]	*n*	Median [IQR]	*n*	Median [IQR]	Δ Median	*p*-value[a]	*n*	Median [IQR]	*n*	Median [IQR]	Δ Median	*p*-value[a]
**Sweets and Snacks**																		
Cakes	749	55 [63]	331	75 [92]	20	0.0002	696	56 [60]	303	85 [109]	29	0.0000	1445	55 [61]	634	77 [95]	22	0.0001
Sweet pie/pastry	162	61 [50]	76	67 [36]	6	0.046	167	71 [53]	105	67 [29]	-4	0.65	329	67 [53]	181	67 [34]	0	0.31
Other desserts	205	119 [60]	86	100 [88]	-19	0.049	200	140 [50]	70	100 [83]	-40	0.0049	405	140 [59]	156	100 [85]	-40	0.0007
Plain sweet biscuits	481	18 [15]	304	20 [18]	2	0.0004	471	22 [20]	291	22 [18]	0	0.011	952	20 [18]	595	20 [17]	0	0.0000
Cream/chocolate biscuits	322	21 [16]	140	21 [18]	0	0.23	391	23 [17]	149	25 [19]	2	0.53	713	22 [17]	289	22 [18]	0	0.68
Savoury biscuits	836	17 [15]	393	17 [18]	0	0.37	760	18 [13]	361	18 [22]	0	0.29	1596	18 [14]	754	17 [18]	-1	0.16
Savoury combi snacks	74	25 [0]	35	24 [42]	-1	0.72	76	25 [0]	40	24 [1]	-1	0.0001	150	25 [0]	75	24 [26]	-1	0.0081
Snack noodles	153	136 [111]	63	145 [288]	9	0.0087	144	136 [176]	68	164 [203]	28	0.0029	297	136 [160]	131	145 [235]	9	0.0001
Muesli bars	299	31 [11]	180	31 [1]	0	0.15	323	31 [11]	215	31 [3]	0	0.16	622	31 [11]	395	31 [1]	0	0.041
**Main Meals**																		
Mince Dish	375	85 [97]	157	192 [270]	107	0.0000	432	107 [127]	161	187 [298]	80	0.0000	807	93 [114]	318	192 [291]	99	0.0000
Mixed meat dish	56	171 [171]	62	165 [148]	-6	0.77	54	167 [200]	79	215 [193]	48	0.19	110	167 [153]	141	204 [220]	37	0.16
Plain Meat	420	68 [68]	142	106 [85]	38	0.0000	460	72 [78]	143	146 [124]	74	0.0000	880	71 [71]	285	120 [94]	49	0.0000
Mixed chicken dish	119	94 [80]	62	173 [209]	79	0.010	123	127 [190]	77	215 [220]	88	0.0008	242	101 [137]	139	198 [228]	97	0.0000
Crumbed Chicken	254	72 [59]	149	88 [81]	16	0.10	226	93 [90]	136	114 [100]	21	0.13	480	81 [75]	285	95 [82]	14	0.021
Plain Chicken	436	71 [79]	167	100 [75]	29	0.0019	479	86 [87]	132	105 [106]	19	0.079	915	75 [87]	299	100 [92]	25	0.0015
Fish	166	77 [85]	81	73 [59]	-4	0.32	150	92 [74]	82	109 [191]	17	0.79	316	92 [74]	163	87 [73]	-5	0.60
Soup	205	260 [238]	95	253 [356]	-7	0.55	179	260 [262]	69	318 [182]	58	0.057	384	260 [260]	164	303 [291]	43	0.44
Sausages	288	57 [34]	120	89 [46]	32	0.0000	357	58 [45]	138	89 [102]	31	0.0000	645	57 [41]	258	89 [98]	32	0.0000
Hamburger	91	137 [95]	87	184 [115]	47	0.044	108	186 [121]	105	185 [136]	-1	0.045	199	167 [110]	192	185 [115]	18	0.0047

Table S3e: portion size changes (grams) between ANCNPAS 2007 and the NNPAS 2011–12, children 2–16 years old

	Females						Males						Persons					
	2007		2011–12				2007		2011–12				2007		2011–12			
Food	*n*	Median [IQR]	*n*	Median [IQR]	Δ Median	*p*-value[a]	*n*	Median [IQR]	*n*	Median [IQR]	Δ Median	*p*-value[a]	*n*	Median [IQR]	*n*	Median [IQR]	Δ Median	*p*-value[a]
Pizza	170	116 [101]	90	163 [128]	47	0.0002	190	153 [189]	110	186 [188]	33	0.33	360	139 [156]	200	185 [176]	46	0.0013
Pie	217	130 [79]	86	130 [99]	0	0.38	272	161 [59]	124	130 [94]	-31	0.48	489	135 [64]	210	130 [95]	-5	0.78
**Other Foods**																		
Chips (not potato)	260	21 [26]	158	22 [17]	1	0.041	264	20 [35]	159	22 [26]	2	0.91	524	21 [29]	317	22 [25]	1	0.16
Potato crisps	425	21 [7]	193	21 [22]	0	0.69	443	21 [29]	213	21 [23]	0	0.0014	868	21 [20]	406	21 [23]	0	0.010
Ice block - cream	111	65 [26]	66	80 [6]	15	0.0000	111	68 [15]	70	80 [9]	12	0.0000	222	68 [19]	136	80 [9]	12	0.0000
Ice block - water	137	76 [6]	113	75 [0]	-1	0.78	134	76 [8]	94	75 [0]	-1	0.94	271	76 [9]	207	75 [0]	-1	0.84
Chocolate	737	19 [25]	290	20 [28]	1	0.50	683	20 [27]	249	20 [31]	0	0.31	1420	20 [27]	539	20 [28]	0	0.23
Lollies	581	14 [17]	226	15 [17]	1	0.86	529	16 [21]	210	15 [18]	-1	0.040	1110	16 [22]	436	15 [17]	-1	0.12
Salad dressing full fat	175	9 [14]	78	20 [7]	11	0.0000	178	10 [14]	108	14 [7]	4	0.0000	353	9 [14]	186	14 [7]	5	0.0000
Nuts	120	18 [29]	45	27 [26]	9	0.20	95	14 [29]	50	24 [27]	10	0.21	215	16 [29]	95	27 [26]	11	0.078
Jam/Honey	690	10 [8]	241	8 [7]	-2	0.17	601	11 [13]	265	10 [16]	-1	0.018	1191	10 [8]	506	10 [14]	0	0.0074
Peanut butter	360	10 [12]	150	9 [4]	-1	0.017	424	12 [13]	188	10 [9]	-2	0.63	784	12 [13]	338	10 [6]	-2	0.059
Vegemite	729	5 [3]	266	6 [4]	1	0.024	676	5 [4]	305	6 [4]	1	0.39	1405	5 [3]	571	6 [4]	1	0.40
Tomato Sauce	646	11 [17]	187	14 [11]	3	0.0000	750	13 [17]	254	14 [14]	1	0.0007	1396	11 [17]	441	14 [11]	3	0.0000
Devon, salami	177	28 [31]	43	28 [24]	0	0.80	203	28 [36]	56	28 [23]	0	0.49	380	28 [32]	99	28 [22]	0	0.71
Bacon/Ham	519	27 [22]	251	17 [15]	-10	0.0000	563	30 [30]	257	17 [17]	-13	0.0000	1082	27 [26]	508	17 [17]	-10	0.0000
Eggs	340	43 [37]	178	51 [37]	8	0.0003	340	43 [49]	146	50 [37]	7	0.0004	680	43 [47]	324	51 [37]	8	0.0000
**Fruit and Vegetables**																		
Hot Chips	527	90 [72]	242	57 [72]	-33	0.0000	488	100 [75]	216	66 [87]	-34	0.0000	1015	100 [78]	458	57 [76]	-43	0.0000
Potato	594	83 [69]	215	83 [97]	0	0.38	599	98 [98]	197	96 [107]	-2	0.56	1193	92 [82]	412	91 [124]	-1	0.32
Peas	324	20 [30]	71	30 [28]	10	0.25	337	23 [30]	66	26 [40]	3	0.28	661	23 [30]	137	26 [38]	3	0.14

Table S3e: portion size changes (grams) between ANCNPAS 2007 and the NNPAS 2011–12, children 2–16 years old

	Females						Males						Persons					
	2007		2011–12				2007		2011–12				2007		2011–12			
**Food**	*n*	Median [IQR]	*n*	Median [IQR]	Δ Median	*p*-value[a]	*n*	Median [IQR]	*n*	Median [IQR]	Δ Median	*p*-value[a]	*n*	Median [IQR]	*n*	Median [IQR]	Δ Median	*p*-value[a]
Broccoli	340	30 [21]	62	46 [58]	16	0.0006	314	30 [30]	77	23 [28]	-7	0.86	654	30 [24]	139	35 [38]	5	0.041
Carrots	834	27 [28]	201	37 [58]	10	0.0000	803	29 [29]	198	37 [63]	8	0.0000	1637	28 [29]	399	37 [63]	9	0.0000
Corn	276	35 [49]	64	70 [112]	35	0.0001	274	39 [46]	77	65 [118]	26	0.0011	550	39 [49]	141	70 [112]	31	0.0000
Tomato	488	51 [45]	157	29 [24]	-22	0.0000	464	56 [49]	122	30 [29]	-26	0.0000	952	51 [48]	279	29 [31]	-22	0.0000
Lettuce	352	15 [21]	91	12 [13]	-3	0.26	341	15 [21]	117	13 [13]	-2	0.44	693	15 [21]	208	13 [13]	-2	0.86
Celery/Cucumber	347	25 [25]	96	26 [46]	1	0.093	298	24 [25]	80	26 [62]	2	0.028	645	25 [25]	176	26 [56]	1	0.0062
Onion	413	11 [14]	54	14 [17]	3	0.56	461	14 [17]	43	9 [13]	-5	0.037	874	12 [15]	97	11 [13]	-1	0.33
Dried fruit	228	25 [12]	67	21 [29]	-4	0.70	255	20 [30]	76	20 [27]	0	0.39	483	21 [28]	143	20 [28]	-1	0.72
Apple	1180	146 [83]	524	153 [49]	7	0.19	1116	146 [83]	590	164 [25]	18	0.0004	2296	146 [83]	1114	164 [28]	18	0.0004
Orange	564	86 [39]	248	75 [56]	-11	0.068	505	86 [57]	194	75 [56]	-11	0.037	1069	86 [53]	442	75 [56]	-11	0.0070
Banana	646	121 [61]	242	98 [24]	-23	0.0010	638	121 [44]	268	98 [0]	-23	0.012	1284	121 [59]	510	98 [0]	-23	0.0000
**Seasonal Fruit**																		
Peach, nectarin, plum or apricot	68	66 [92]	89	151 [76]	85	0.0000	47	73 [85]	67	145 [79]	72	0.012	115	72 [94]	156	151 [76]	79	0.0000
Mixed Berries/Grapes	387	60 [84]	267	54 [60]	-6	0.51	330	60 [71]	244	60 [60]	0	0.67	717	60 [84]	511	60 [60]	0	0.44
Melon	203	107 [116]	111	135 [85]	28	0.37	206	130 [164]	88	150 [132]	20	0.70	409	121 [145]	199	150 [95]	29	0.79

[a]*p*-values without Bonferroni correction
