# Supplementary material for: Trends in Food and Beverage Portion Sizes in Australian Children; a Time-Series Analysis Comparing 2007 and 2011–2012 National Data
Source: Children (Basel). 2017 Aug 4;4(8):69. doi: 10.3390/children4080069 (PMC5575591; doi:10.3390/children4080069)
Supplement: Supplementary file 1 [file children-04-00069-s001.zip › Table S4_adj.pdf]

Table S4. Significant changes in the portion size of ACAES items between 2007 and 2011–12, in the whole sample and for several population groups[a]

Food	2–4 years			5–8 years			9–12 years			13–16 years			2–16 years		
	Females	Males	All	Females	Males	All	Females	Males	All	Females	Males	All	Females	Males	All
**Drinks**															
Water	↑	↑	↑	↑	↑	↑	↑	↑	↑	↑	↑	↑	↑	↑	↑
Fruit juice	↑	↑	↑						↑	↑		↑	0↑	↑	↑
Tea or coffee											↓	↓			↓
**Dairy Foods**															
Whole milk					0↓	0↓		↓	↓					0↓	0↓
Flavoured milk	↑								↑	↑			↑		↑
Ice cream														↑	↑
Cheese			↑												
**Breads and Cereals**															
White bread		↑	↑												↑
Wholemeal bread															↑
Rice														↓	↓
Pasta					↑	↑		↑	↑	↑	↑	↑	↑	↑	↑
**Sweets and Snacks**															
Cakes							↑		↑		↑	↑	↑	↑	↑
Plain sweet biscuits													↑		0↑
Savoury combination snacks						↓								↓	
Snack noodles									↑						↑
**Main meals**															
Mince dish								↑	↑	↑		↑	↑	↑	↑
Plain meat	↑	↑	↑		↑	↑			↑	↑	↑	↑	↑	↑	↑
Mixed chicken dish															↑
Sausages	↑	↑	↑	↑	↑	↑	↑	↑	↑		↑	↑	↑	↑	↑
Pizza									↑				↑		
**Other Foods**															

Ice block – cream						↑	↑						↑	↑	↑
Salad dressing full fat							↑		↑			↑	↑	↑	↑
Tomato sauce						↑							↑		↑
Bacon, ham	↓			↓	↓	↓	↓		↓		↓		↓	↓	↓
Eggs													↑	↑	↑
**Fruit and vegetables**															
Hot chips	↓	↓	↓	↓	↓	↓	↓		↓	↓	↓	↓	↓	↓	↓
Broccoli													↑		
Carrots	↑	↑	↑	↑		↑							↑	↑	↑
Corn					↑	↑							↑		↑
Tomato				↓		↓	↓		↓	↓	↓	↓	↓	↓	↓
Apple	↑	↑	↑									↓		↑	↑
Orange	↑		↑												
Banana				↓		↓			↓	↑		↓			↓
**Seasonal fruits**															
Peach, nectarine, plum, apricot													↑		↑

[a] Only foods of which the portion size changed significantly ($p<0.05$ (Bonferroni corrected) in the whole sample or in at least one of the population subgroups are presented.

↑ indicates an increase in portion size in 2011–12, when compared to 2007

↓ indicated a decrease in portion size in 2011–12, when compared to 2007

- indicates that the change in portion size was either not significant ($p>0.05$) or the food was eaten by <5% of the 2011–12 population

$0^{\uparrow}$ Change in median portion size was 0, however the Mann Whitney U test found a significant increase in portion size distributions in 2011–12, when compared to 2007

$0^{\downarrow}$ Change in median portion size was 0, however the Mann Whitney U test found a significant decrease in portion size distributions in 2011–12, when compared to 2007
