# Supplementary material for: Trends in Food and Beverage Portion Sizes in Australian Children; a Time-Series Analysis Comparing 2007 and 2011–2012 National Data
Source: Children (Basel). 2017 Aug 4;4(8):69. doi: 10.3390/children4080069 (PMC5575591; doi:10.3390/children4080069)
Supplement: Supplementary file 1 [file children-04-00069-s001.zip › Table S5_adj.pdf]

Table S5: ACAES items that were consumed by less than 5 percent of the children in all NNPAS 2011–12 age groups

ACAES FFQ food group	ACAES FFQ food item	Percentage (%)*
Drinks (D)	Beer	<0.1
	Wine or wine coolers	0.2
	Spirits	<0.1
Dairy foods (DF)	Soy milk	1.5
	Skim milk	4.0
	Cream or sour cream	2.1
	Frozen yoghurt	0.7
	Cottage cheese or ricotta	0.5
	Cheese spread, cream cheese	2.4
Breads and cereal (B)	Muesli	1.0
	English muffin, bagel or crumpet	2.6
	Other grains (couscous, burghul)	1.2
	Noodles	2.3
Sweets and snacks (S)	Sweet combination snacks	0
	Fruit bars	0.9
	Snack bars	0.6
Main meals (M)	Plain pork	2.9
	Liver – beef, calf, chicken	<0.1
	Canned tuna, salmon, sardines incl. patties	2.4
	Other seafood	1.1
	Tacos, burritos, enchiladas	2.0
	Hot dog	2.1
	Savoury pastries	1.7
	Hash browns, potato scallops	1.8
Other foods (O)	Salad dressing, low fat	0.8
	Jelly	2.2
Fruit and vegetables (F)	Pumpkin	2.6
	Sweet potato	2.5
	Cauliflower	2.1
	Green beans	3.2
	Spinach	1.6
	Cabbage or Brussel sprouts	0.7
	Zucchini, eggplant or squash	1.3
	Capsicum	1.5
	Mushrooms	1.1
	Avocado	1.9
	Soybeans, tofu	0.4
	Baked beans	1.6
	Other beans or lentils	0.8
	Canned fruit	2.8
	Fruit salad	2.6
Seasonal fruits (FS)	Mango or pawpaw	1.9
	Pineapple	0.8

*Percentage of children in the entire 2011–12 population consuming the ACAES item
