# Supplementary material for: Trends in Food and Beverage Portion Sizes in Australian Children; a Time-Series Analysis Comparing 2007 and 2011–2012 National Data
Source: Children (Basel). 2017 Aug 4;4(8):69. doi: 10.3390/children4080069 (PMC5575591; doi:10.3390/children4080069)
Supplement: Supplementary file 1 [file children-04-00069-s001.zip › Table S6_adj.pdf]

Table S6a: Percentage of Australian children aged 2–4 years and 5–8 years consuming selected ACAES food categories, in the 2007 ANCNPAS and the 2011–12 NNPAS

		2 – 4-year-old participants						5 – 8-year-old participants					
ACAES FFQ Item		ANCNPAS (2007)			NNPAS (2011–12)			ANCNPAS (2007)			NNPAS (2011–12)		
		Female *n*=715	Male *n*=776	Total *n*=1491	Female *n*=345	Male *n*=330	Total *n*=675	Female *n*=474	Male *n*=481	Total *n*=955	Female *n*=283	Male *n*=295	Total *n*=578
**Drinks**													
D1	DIET soft drink eg. Diet coke	18 (2.5%)	19 (2.4%)	37 (2.5%)	2 (0.6%)	4 (1.2%)	6 (0.9%)	21 (4.4%)	19 (4.0%)	40 (4.2%)	6 (2.1%)	6 (2.0%)	12 (2.1%)
D2	Soft drink (NOT DIET) including flavoured mineral water	76 (10.6%)	88 (11.3%)	164 (11.0%)	23 (6.7%)	21 (6.4%)	44 (6.5%)	98 (20.4%)	107 (22.2%)	205 (21.5%)	66 (23.3%)	46 (15.6%)	112 (19.4%)
D3	Water - including bottled water. unflavoured mineral water, tap water	681 (95.2%)	723 (93.2%)	1404 (94.2%)	304 (88.1%)	300 (90.9%)	604 (89.5%)	458 (95.2%)	455 (94.6%)	913 (95.6%)	270 (95.4%)	280 (94.9%)	550 (95.2%)
D4	Fruit juice-based drinks	337 (47.1%)	353 (45.5%)	690 (46.3%)	132 (38.3%)	145 (43.9%)	277 (41.0%)	211 (43.9%)	241 (50.1%)	452 (47.3%)	110 (38.9%)	122 (41.4%)	232 (40.1%)
D5	Cordial or 'make up'	126 (17.6%)	178 (22.9%)	304 (20.4%)	40 (11.6%)	40 (12.1%)	80 (11.9%)	102 (21.2%)	99 (20.6%)	201 (21.0%)	22 (7.8%)	40 (13.6%)	62 (10.7%)
D6	Tea or Coffee	21 (2.9%)	28 (3.6%)	49 (3.3%)	11 (3.2%)	8 (2.4%)	19 (2.8%)	29 (6.0%)	31 (6.4%)	60 (6.3%)	16 (5.7%)	8 (2.7%)	24 (4.2%)
D7	Beer	0 (0%)	0 (0%)	0 (0%)	0 (0%)	0 (0%)	0 (0%)	0 (0%)	0 (0%)	0 (0%)	0 (0%)	0 (0%)	0 (0%)
D8	Wine or wine coolers	7 (1.0%)	8 (1.0%)	15 (1.0%)	2 (0.6%)	1 (0.3%)	3 (0.4%)	1 (0.2%)	2 (0.4%)	3 (0.3%)	1 (0.4%)	0 (0%)	1 (0.2%)
D9	Spirits	0 (0%)	0 (0%)	0 (0%)	0 (0%)	0 (0%)	0 (0%)	0 (0%)	0 (0%)	0 (0%)	0 (0%)	0 (0%)	0 (0%)

Table S6a: Percentage of Australian children aged 2–4 years and 5–8 years consuming selected ACAES food categories, in the 2007 ANCNPAS and the 2011–12 NNPAS

		2 – 4-year-old participants						5 – 8-year-old participants					
ACAES FFQ Item		ANCNPAS (2007)			NNPAS (2011–12)			ANCNPAS (2007)			NNPAS (2011–12)		
		Female *n*=715	Male *n*=776	Total *n*=1491	Female *n*=345	Male *n*=330	Total *n*=675	Female *n*=474	Male *n*=481	Total *n*=955	Female *n*=283	Male *n*=295	Total *n*=578
**Dairy foods:**													
DF1 + DF3	Soy Milk	26 (3.6%)	29 (3.7%)	55 (3.7%)	8 (2.3%)	11 (3.3%)	19 (2.4%)	9 (1.9%)	5 (1.0%)	14 (1.5%)	7 (2.5%)	3 (1.0%)	10 (1.7%)
DF1 + DF3	Whole Milk	480 (67.1%)	549 (70.7%)	1029 (69.0%)	224 (64.9%)	214 (64.8%)	438 (64.9%)	236 (49.8%)	270 (56.1%)	506 (53.0%)	147 (51.9%)	152 (51.5%)	299 (51.7%)
DF1 + DF3	Reduced Fat Milk	123 (17.2%)	128 (16.5%)	251 (16.8%)	49 (14.2%)	35 (10.6%)	84 (12.4%)	95 (20.0%)	102 (21.2%)	197 (20.6%)	45 (15.9%)	45 (15.3%)	90 (15.6%)
DF1 + DF3	Skim Milk	23 (3.2%)	19 (2.4%)	42 (2.8%)	16 (4.6%)	8 (2.4%)	24 (3.6%)	23 (4.9%)	23 (4.8%)	46 (4.8%)	9 (3.2%)	12 (4.1%)	21 (3.6%)
DF2	Flavoured milk	55 (7.7%)	75 (9.7%)	130 (8.7%)	25 (7.2%)	25 (7.6%)	50 (7.4%)	69 (14.6%)	55 (11.4%)	124 (13.0%)	26 (9.2%)	32 (10.8%)	58 (10.0%)
DF4	Cream or sour cream	25 (3.5%)	24 (3.1%)	51 (3.4%)	6 (1.7%)	4 (1.2%)	10 (1.5%)	21 (4.4%)	25 (5.2%)	46 (4.8%)	8 (2.8%)	5 (1.7%)	13 (2.2%)
DF5	Ice cream	120 (16.8%)	107 (13.8%)	227 (15.2%)	43 (12.5%)	46 (13.9%)	89 (13.2%)	117 (24.7%)	101 (20.1%)	218 (22.8%)	38 (13.4%)	53 (18.0%)	91 (15.7%)
DF6	Frozen yoghurt	8 (1.1%)	3 (0.4%)	11 (0.7%)	1 (0.3%)	3 (0.9%)	4 (0.6%)	6 (1.3%)	5 (1.0%)	11 (1.2%)	1 (0.4%)	1 (0.3%)	2 (0.3%)
DF7	Yoghurt (not frozen) plain or flavoured	256 (35.8%)	272 (35.1%)	528 (35.4%)	114 (33.0%)	101 (30.6%)	215 (31.9%)	96 (20.3%)	105 (21.8%)	201 (21.0%)	57 (20.1%)	56 (19.0%)	113 (19.6%)
DF8	Cottage cheese or ricotta	4 (0.6%)	4 (0.5%)	8 (0.5%)	4 (1.2%)	0 (0%)	4 (0.6%)	0 (0%)	3 (0.6%)	3 (0.3%)	2 (0.7%)	0 (0%)	2 (0.3%)
DF9	Cheese including cheese on sandwiches, biscuits or on toast	343 (48%)	371 (47.8%)	714 (47.9%)	123 (35.7%)	116 (35.2%)	239 (35.4%)	177 (37.3%)	198 (41.2%)	375 (39.3%)	83 (29.3%)	93 (31.5%)	176 (30.4%)

Table S6a: Percentage of Australian children aged 2–4 years and 5–8 years consuming selected ACAES food categories, in the 2007 ANCNPAS and the 2011–12 NNPAS

ACAES FFQ Item		2 – 4-year-old participants						5 – 8-year-old participants					
		ANCNPAS (2007)			NNPAS (2011–12)			ANCNPAS (2007)			NNPAS (2011–12)		
		Female *n*=715	Male *n*=776	Total *n*=1491	Female *n*=345	Male *n*=330	Total *n*=675	Female *n*=474	Male *n*=481	Total *n*=955	Female *n*=283	Male *n*=295	Total *n*=578
DF10	Cheese spread, cream cheese	43 (6%)	42 (5.4%)	85 (5.7%)	8 (2.3%)	8 (2.4)	16 (2.4%)	24 (5.1%)	15 (3.1%)	39 (4.1%)	9 (3.2%)	9 (3.1%)	18 (3.1%)
**Breads and Cereal:**													
B1	Muesli	12 (1.7%)	13 (1.7%)	25 (1.7%)	1 (0.3%)	4 (1.2%)	5 (0.7%)	4 (0.8%)	3 (0.6%)	7 (0.7%)	2 (0.7%)	3 (1.0%)	5 (0.9%)
B2	Cooked porridge	55 (7.7%)	55 (7.1%)	110 (7.4%)	37 (10.7%)	24 (7.3%)	61 (9.0%)	29 (6.1%)	16 (3.3%)	45 (4.7%)	15 (5.3%)	22 (7.5%)	37 (6.4%)
B3	Breakfast cereal	418 (58.5%)	462 (59.5%)	880 (59%)	174 (50.4%)	168 (50.9%)	342 (50.7%)	240 (50.6%)	312 (64.9%)	552 (57.8%)	136 (48.1%)	156 (52.9%)	292 (50.5%)
B5	Bread, pita bread, roll – White	336 (47.0%)	395 (50.9%)	731 (49.0%)	141 (40.9%)	140 (42.4%)	281 (41.6%)	271 (57.2%)	273 (56.7%)	544 (57%)	154 (54.4%)	162 (54.9%)	316 (54.7%)
B5	Bread, pita bread, roll – Mixed Grain	84 (11.7%)	122 (15.7%)	206 (13.8%)	37 (10.7%)	52 (15.8%)	89 (13.2%)	51 (10.8%)	45 (9.4%)	96 (10.1%)	26 (9.2%)	26 (8.8%)	52 (9.0%)
B5	Bread, pita bread, roll - Wholemeal	198 (27.7%)	179 (23.1%)	377 (25.3%)	81 (23.5%)	69 (20.9%)	150 (22.2%)	85 (17.9%)	107 (22.2%)	192 (20.1%)	51 (18.0%)	56 (19.0%)	107 (18.5%)
B6	English muffin, bagel or crumpet	34 (4.8%)	32 (4.1%)	66 (4.4%)	11 (3.2%)	4 (1.2%)	15 (2.2%)	18 (3.8%)	17 (3.5%)	35 (3.7%)	6 (2.1%)	6 (2.0%)	12 (2.1%)
B7	Rice	112 (15.7%)	115 (14.8%)	227 (15.2%)	60 (17.4%)	75 (22.7%)	135 (20.0%)	68 (14.3%)	59 (12.3%)	127 (13.3%)	49 (17.3%)	47 (15.9%)	96 (16.6%)
B8	Other grains eg. cous cous, burghul	7 (1.0%)	5 (0.6%)	12 (0.8%)	4 (1.2%)	10 (3.0%)	14 (2.1%)	2 (0.4%)	1 (0.2%)	3 (0.3%)	1 (0.4%)	2 (0.7%)	3 (0.5%)
B9	Noodles	9 (1.3%)	19 (2.4%)	28 (1.9%)	6 (1.7%)	7 (2.1%)	13 (1.9%)	7 (1.5%)	9 (1.9%)	16 (1.7%)	5 (1.8%)	8 (2.7%)	13 (2.2%)

Table S6a: Percentage of Australian children aged 2–4 years and 5–8 years consuming selected ACAES food categories, in the 2007 ANCNPAS and the 2011–12 NNPAS

		2 – 4-year-old participants						5 – 8-year-old participants					
ACAES FFQ Item		ANCNPAS (2007)			NNPAS (2011–12)			ANCNPAS (2007)			NNPAS (2011–12)		
		Female *n*=715	Male *n*=776	Total *n*=1491	Female *n*=345	Male *n*=330	Total *n*=675	Female *n*=474	Male *n*=481	Total *n*=955	Female *n*=283	Male *n*=295	Total *n*=578
B10	Pasta	208 (29.1%)	232 (29.9%)	440 (29.5%)	88 (25.5%)	67 (20.3%)	155 (23.0%)	94 (19.8%)	122 (25.4%)	218 (22.8%)	40 (14.1%)	33 (11.2%)	73 (12.6%)
**Sweets and Snacks:**													
S1	Cakes, sweet muffins, scones, pikelets, pancakes, hot cakes	170 (23.8%)	193 (24.9%)	363 (24.3%)	62 (18.0%)	64 (19.4%)	126 (18.7%)	145 (30.6%)	123 (25.6%)	268 (28.1%)	75 (26.5%)	74 (25.1%)	149 (25.8%)
S2	Sweet pies or sweet pastries	31 (4.3%)	38 (4.9%)	69 (4.6%)	8 (2.3%)	15 (4.5%)	23 (3.4%)	25 (5.3%)	28 (5.8%)	53 (5.5%)	17 (6.0%)	26 (8.8%)	43 (7.4%)
S3	Other puddings or desserts (not ice cream)	60 (8.4%)	78 (10.1%)	138 (9.3%)	22 (6.4%)	19 (5.8%)	41 (6.1%)	39 (8.2%)	37 (7.7%)	76 (8.0%)	19 (6.7%)	13 (4.4%)	32 (5.5%)
S4	Plain sweet biscuits	161 (22.5%)	155 (20.0%)	316 (21.2%)	92 (26.7%)	84 (25.5%)	176 (26.1%)	93 (19.6%)	88 (18.3%)	181 (19.0%)	70 (24.7%)	68 (23.1%)	138 (23.9%)
S5	Cream or chocolate biscuits	75 (10.5%)	95 (12.2%)	170 (11.4%)	30 (8.7%)	29 (8.8%)	59 (8.7%)	65 (13.7%)	71 (14.8%)	136 (14.2%)	29 (10.2%)	35 (11.9%)	64 (11.1%)
S6	Dry or savoury biscuits, crispbread, crackers	269 (37.6%)	268 (34.5%)	537 (36.0%)	116 (33.6%)	97 (29.4%)	212 (31.4%)	149 (31.4%)	139 (28.9%)	288 (30.2%)	82 (29.0%)	83 (28.1%)	165 (28.5%)
S7	Savoury combination snacks	21 (2.9%)	22 (2.8%)	43 (2.9%)	5 (1.4%)	12 (3.6%)	17 (2.5%)	25 (5.3%)	27 (5.6%)	52 (5.4%)	16 (5.7%)	15 (5.1%)	31 (5.4%)
S8	Sweet combination snacks	-	-	-	-	-	-	-	-	-	-	-	-

Table S6a: Percentage of Australian children aged 2–4 years and 5–8 years consuming selected ACAES food categories, in the 2007 ANCNPAS and the 2011–12 NNPAS

		2 – 4-year-old participants						5 – 8-year-old participants					
ACAES FFQ Item		ANCNPAS (2007)			NNPAS (2011–12)			ANCNPAS (2007)			NNPAS (2011–12)		
		Female *n*=715	Male *n*=776	Total *n*=1491	Female *n*=345	Male *n*=330	Total *n*=675	Female *n*=474	Male *n*=481	Total *n*=955	Female *n*=283	Male *n*=295	Total *n*=578
S9	Snack noodles	29 (4.1%)	25 (3.2%)	54 (3.6%)	14 (4.1%)	13 (3.9%)	27 (4.0%)	33 (7%)	31 (6.4%)	64 (6.7%)	13 (4.6%)	13 (4.4%)	26 (4.5%)
S10	Fruit bars	0 (0%)	0 (0%)	0 (0%)	4 (1.2%)	2 (0.6%)	6 (0.9%)	0 (0%)	0 (0%)	0 (0%)	3 (1.1%)	2 (0.7%)	5 (0.9%)
S11	Snack bars	42 (5.9%)	46 (5.9%)	88 (5.9%)	0 (0%)	2 (0.6%)	2 (0.3%)	27 (5.7%)	39 (8.1%)	66 (6.9%)	3 (1.1%)	2 (0.7%)	5 (0.9%)
S12	Muesli bars	56 (7.8%)	59 (7.6%)	115 (7.7%)	34 (10.0%)	33 (10.0%)	67 (10.0%)	54 (11.4%)	630 (13.1%)	117 (12.3%)	37 (13.1%)	53 (18.0%)	90 (15.6%)
**Main Meals:**													
M1	Mince dish	121 (16.9%)	120 (15.5%)	241 (16.2%)	39 (11.3%)	45 (13.6%)	84 (12.4%)	53 (11.2%)	79 (16.4%)	132 (13.8%)	26 (9.2%)	26 (8.8%)	52 (9.0%)
M2-3, 5 M11–12,14	Mixed meat dish	12 (1.7%)	20 (2.5%)	32 (2.2%)	15 (4.3%)	15 (4.5%)	30 (4.4%)	6 (1.3%)	6 (1.2%)	12 (1.2%)	5 (1.8%)	21 (7.1%)	26 (4.5%)
M4	Plain meat (beef or lamb) WITHOUT vegetables or salad	116 (16.2%)	123 (15.9%)	239 (16.0%)	30 (8.7%)	29 (8.8%)	59 (8.7%)	65 (13.7%)	79 (16.4%)	144 (15.1%)	24 (8.5%)	24 (8.1%)	48 (8.3%)
M6-7, M10	Mixed chicken dish	29 (4.0%)	26 (3.4%)	55 (3.7%)	12 (3.5%)	14 (4.2%)	26 (3.9%)	17 (3.6%)	16 (3.3%)	33 (3.5%)	7 (2.5%)	18 (6.1%)	25 (4.3%)
M8	Chicken crumbed	69 (9.7%)	49 (6.3%)	118 (7.9%)	39 (11.3%)	40 (12.1%)	79 (11.7%)	57 (12.0%)	51 (10.6%)	108 (11.3%)	39 (13.8%)	27 (9.2%)	66 (11.4%)
M9	Plain chicken WITHOUT vegetables	115 (16.1%)	131 (16.9%)	246 (16.5%)	53 (15.4%)	43 (13.0%)	96 (14.2%)	78 (16.5%)	81 (16.8%)	159 (16.6%)	34 (12.0%)	25 (8.5%)	59 (10.2%)

Table S6a: Percentage of Australian children aged 2–4 years and 5–8 years consuming selected ACAES food categories, in the 2007 ANCNPAS and the 2011–12 NNPAS

ACAES FFQ Item		2 – 4-year-old participants						5 – 8-year-old participants					
		ANCNPAS (2007)			NNPAS (2011–12)			ANCNPAS (2007)			NNPAS (2011–12)		
		Female *n*=715	Male *n*=776	Total *n*=1491	Female *n*=345	Male *n*=330	Total *n*=675	Female *n*=474	Male *n*=481	Total *n*=955	Female *n*=283	Male *n*=295	Total *n*=578
M13	Plain pork WITHOUT vegetables	15 (2.1%)	21 (2.7%)	36 (2.4%)	5 (1.4%)	7 (2.1%)	12 (1.8%)	12 (2.5%)	14 (2.9%)	26 (2.7%)	7 (2.5%)	9 (3.1%)	16 (2.8%)
M15	Liver – beef, calf, chicken	0 (0%)	0 (0%)	0 (0%)	0 (0%)	0 (0%)	0 (0%)	2 (0.4%)	1 (0.2%)	3 (0.3%)	0 (0%)	1 (0.3%)	1 (0.2%)
M16-17	Fish	61 (8.5%)	60 (7.7%)	121 (8.1%)	25 (7.2%)	27 (8.2%)	52 (7.7%)	41 (8.6%)	29 (6.0%)	70 (7.3%)	23 (8.1%)	19 (6.4%)	42 (7.3%)
M18	Canned tuna, salmon, sardines including patties	25 (3.5%)	29 (3.7%)	54 (3.6%)	9 (2.6%)	4 (1.2%)	13 (1.9%)	19 (4.0%)	17 (3.5%)	36 (3.8%)	6 (2.1%)	3 (1.0%)	9 (1.6%)
M19	Other seafood	9 (1.3%)	14 (1.8%)	23 (1.5%)	2 (0.6%)	5 (1.5%)	7 (1.0%)	10 (2.1%)	14 (2.9%)	24 (2.5%)	1 (0.4%)	5 (1.7%)	6 (1.0%)
M20-21	Creamy or Clear soup	49 (6.9%)	55 (7.1%)	104 (7.0%)	34 (9.9%)	18 (5.5%)	52 (7.7%)	34 (7.2%)	29 (6.0%)	63 (6.6%)	15 (5.3%)	18 (6.1%)	33 (5.7%)
M22	Tacos, burritos, enchiladas	7 (1.0%)	7 (0.9%)	14 (0.9%)	5 (1.4%)	0 (0%)	5 (0.7%)	10 (2.1%)	12 (2.5%)	22 (2.3%)	10 (3.5%)	9 (3.1%)	19 (3.3%)
M23	Sausages, frankfurts, Pluto Pup	89 (12.4%)	104 (13.4%)	193 (12.9%)	39 (11.3%)	31 (9.4%)	70 (10.4%)	59 (12.4%)	69 (14.3%)	128 (13.4%)	28 (10.0%)	38 (12.9%)	66 (11.4%)
M24	Hamburger - all types	7 (1%)	11 (1.4%)	18 (1.2%)	13 (3.8%)	10 (3.0%)	23 (3.4%)	15 (3.2%)	23 (4.8%)	38 (4.0%)	14 (4.9%)	14 (4.7%)	28 (4.8%)
M25	Pizza	27 (3.8%)	33 (4.3%)	60 (4.0%)	22 (6.4%)	22 (6.7%)	44 (6.5%)	34 (7.2%)	29 (6.0%)	63 (6.6%)	20 (7.1%)	23 (7.8%)	43 (7.4%)
M26	Pie, sausage roll, chiko roll	28 (3.9%)	53 (6.8%)	81 (5.4%)	20 (5.8%)	18 (5.5%)	38 (5.6%)	54 (11.4%)	42 (8.7%)	96 (10.1%)	18 (6.4%)	21 (7.1%)	39 (6.7%)

Table S6a: Percentage of Australian children aged 2–4 years and 5–8 years consuming selected ACAES food categories, in the 2007 ANCNPAS and the 2011–12 NNPAS

ACAES FFQ Item		2 – 4-year-old participants						5 – 8-year-old participants					
		ANCNPAS (2007)			NNPAS (2011–12)			ANCNPAS (2007)			NNPAS (2011–12)		
		Female *n*=715	Male *n*=776	Total *n*=1491	Female *n*=345	Male *n*=330	Total *n*=675	Female *n*=474	Male *n*=481	Total *n*=955	Female *n*=283	Male *n*=295	Total *n*=578
M27	Hot dog	-	-	-	3 (0.9%)	0 (0%)	3 (0.4%)	-	-	-	3 (1.1%)	13 (4.4%)	16 (2.8%)
M28	Savoury pastries	13 (1.8%)	24 (3.1%)	37 (2.5%)	5 (1.4%)	7 (2.1%)	12 (1.8%)	12 (2.5%)	13 (2.7%)	25 (2.6%)	3 (1.1%)	3 (1.0%)	6 (1.0%)
M29	Hash browns, potato scallops	7 (1.0%)	11 (1.4%)	18 (1.2%)	7 (2.0%)	5 (1.5%)	12 (1.8%)	9 (1.9%)	14 (2.9%)	23 (2.4%)	2 (0.7%)	3 (1.0%)	5 (0.9%)
**Other Foods:**													
O1	Chips (not potato)	61 (8.5%)	75 (9.7%)	136 (9.1%)	35 (10.1%)	27 (8.2%)	62 (9.2%)	47 (9.9%)	65 (13.5%)	112 (11.7%)	39 (13.8%)	46 (15.6%)	85 (14.7%)
O2	Potato chips or crisps	69 (9.7%)	70 (9.0%)	139 (9.3%)	34 (9.9%)	34 (10.3%)	68 (10.1%)	85 (17.9%)	97 (20.2%)	182 (19.1%)	50 (17.7%)	45 (15.3%)	95 (16.4%)
O3	Ice block – creamy	21 (2.9%)	26 (3.4%)	47 (3.2%)	9 (2.6%)	11 (3.3%)	20 (3.0%)	23 (4.9%)	23 (4.8%)	46 (4.8%)	18 (6.4%)	24 (8.1%)	42 (7.3%)
O4	Ice block – water	28 (3.9%)	36 (4.6%)	65 (4.4%)	25 (7.2%)	20 (6.1%)	45 (6.7%)	40 (8.4%)	35 (7.3%)	75 (7.9%)	36 (12.7%)	31 (10.5%)	67 (11.6%)
O5	Chocolate	142 (19.9%)	145 (18.7%)	287 (19.2%)	63 (18.3%)	40 (12.1%)	103 (15.3%)	130 (27.4%)	128 (26.6%)	258 (27.0%)	57 (20.1%)	52 (17.6%)	109 (18.9%)
O6	Lollies without chocolate	114 (15.9%)	104 (13.4%)	218 (14.6%)	44 (12.8%)	47 (14.2%)	91 (13.5%)	111 (23.4%)	103 (21.4%)	214 (22.4%)	52 (18.4%)	53 (18.0%)	105 (18.2%)
O7	Low fat salad dressing or mayonnaise	11 (1.5%)	10 (1.3%)	21 (1.4%)	1 (0.3%)	0 (0%)	1 (0.1%)	5 (1.1%)	11 (2.3%)	16 (1.7%)	1 (0.4%)	1 (0.3%)	2 (0.3%)
O8	Salad dressing or mayonnaise - not low fat	34 (4.8%)	24 (3.1%)	58 (3.9%)	12 (3.5%)	12 (3.6%)	24 (3.6%)	21 (4.4%)	24 (5.0%)	45 (4.7%)	11 (3.9%)	20 (6.8%)	31 (5.4%)
O9	Nuts	34 (4.8%)	24 (3.1%)	58 (3.9%)	15 (4.3%)	19 (5.8%)	34 (5.0%)	18 (3.8%)	17 (3.5%)	35 (3.7%)	4 (1.4%)	12 (4.1%)	16 (2.8%)

Table S6a: Percentage of Australian children aged 2–4 years and 5–8 years consuming selected ACAES food categories, in the 2007 ANCNPAS and the 2011–12 NNPAS

ACAES FFQ Item		2 – 4-year-old participants						5 – 8-year-old participants					
		ANCNPAS (2007)			NNPAS (2011–12)			ANCNPAS (2007)			NNPAS (2011–12)		
		**Female** *n*=715	**Male** *n*=776	**Total** *n*=1491	**Female** *n*=345	**Male** *n*=330	**Total** *n*=675	**Female** *n*=474	**Male** *n*=481	**Total** *n*=955	**Female** *n*=283	**Male** *n*=295	**Total** *n*=578
O10	Jam, honey, golden syrup, marmalade	180 (25.2%)	211 (27.2%)	391 (26.2%)	81 (23.5%)	75 (22.7%)	156 (23.1%)	104 (21.9%)	102 (21.2%)	206 (21.6%)	62 (21.9%)	66 (22.4%)	128 (22.1%)
O11	Peanut butter, Nutella	97 (13.6%)	105 (13.5%)	202 (13.5%)	44 (12.8%)	54 (16.4%)	98 (14.5%)	76 (16.0%)	78 (16.2%)	154 (16.1%)	34 (12.0%)	35 (11.9%)	69 (11.9%)
O12	Vegemite, Mighty Mite, Promite, Marmite	214 (30.0%)	278 (35.8%)	492 (33.0%)	81 (23.5%)	88 (26.7%)	169 (25.0%)	124 (26.2%)	112 (23.3%)	236 (24.7%)	60 (21.2%)	58 (19.7%)	118 (20.4%)
O13	Tomato sauce, barbecue sauce	134 (18.7%)	158 (20.4%)	292 (19.6%)	37 (10.7%)	46 (13.9%)	83 (12.3%)	126 (26.6%)	121 (25.2%)	247 (25.8%)	32 (11.3%)	49 (16.6%)	81 (14.0%)
O14	Devon, salami	48 (6. 7%)	57 (7.3%)	105 (7.0%)	10 (2.9%)	11 (3.3%)	21 (3.1%)	55 (11.6%)	37 (7.7%)	92 (9.6%)	13 (4.6%)	15 (5.1%)	28 (4.8%)
O15	Bacon, ham	132 (18.5%)	148 (19.1%)	280 (18.8%)	52 (15.1%)	46 (13.9%)	98 (14.5%)	94 (19.8%)	112 (23.3%)	206 (21.5%)	55 (19.4%)	55 (18.6%)	110 (19.0%)
O16	Eggs	103 (14.4%)	100 (12.9%)	203 (13.6%)	47 (13.6%)	35 (10.6%)	82 (12.1%)	59 (12.4%)	65 (13.5%)	124 (13.0%)	46 (16.3%)	31 (10.5%)	77 (13.3%)
O17	Jelly	17 (2.4%)	30 (3.9%)	47 (3.2%)	6 (1.7%)	8 (2.4%)	14 (2.1%)	20 (4.2%)	11 (2.3%)	31 (3.2%)	7 (2.5%)	9 (3.1%)	16 (2.8%)
**Fruit and Vegetables**													
F1-2	Hot chips bought from a shop or cooked at home	112 (15.7%)	113 (14.6%)	225 (15.1%)	61 (17.7%)	45 (13.6%)	106 (15.7%)	101 (21.3%)	96 (20.0%)	197 (20.6%)	50 (17.7%)	46 (15.6%)	96 (16.6%)
F3	Potato boiled, mashed, baked	184 (25.7%)	203 (26.2%)	387 (30.0%)	59 (17.1%)	48 (14.5%)	107 (15.9%)	108 (22.8%)	102 (21.2%)	210 (22.0%)	47 (16.6%)	44 (14.9%)	91 (15.7%)
F4	Pumpkin	64 (9.0%)	59 (7.6%)	123 (8.2%)	12 (3.5%)	9 (2.7%)	26 (3.9%)	25 (5.3%)	27 (5.6%)	52 (5.4%)	5 (1.8%)	2 (0.7%)	7 (1.2%)

Table S6a: Percentage of Australian children aged 2–4 years and 5–8 years consuming selected ACAES food categories, in the 2007 ANCNPAS and the 2011–12 NNPAS

		**2 – 4-year-old participants**						**5 – 8-year-old participants**					
**ACAES FFQ Item**		**ANCNPAS (2007)**			**NNPAS (2011–12)**			**ANCNPAS (2007)**			**NNPAS (2011–12)**		
		**Female** ***n*=715**	**Male** ***n*=776**	**Total** ***n*=1491**	**Female** ***n*=345**	**Male** ***n*=330**	**Total** ***n*=675**	**Female** ***n*=474**	**Male** ***n*=481**	**Total** ***n*=955**	**Female** ***n*=283**	**Male** ***n*=295**	**Total** ***n*=578**
F5	Sweet potato	37 (5.2%)	30 (3.9%)	67 (4.5%)	10 (2.9%)	13 (3.9%)	23 (3.4%)	8 (1.7%)	12 (2.5%)	20 (2.1%)	6 (2.1%)	13 (4.4%)	19 (3.3%)
F6	Cauliflower	50 (7.0%)	41 (5.3%)	91 (6.1%)	5 (1.4%)	10 (3.0%)	15 (2.2%)	24 (5.1%)	27 (5.6%)	51 (5.3%)	3 (1.1%)	10 (3.4%)	13 (2.2%)
F7	Green beans	51 (7.1%)	59 (7.6%)	110 (7.3%)	12 (3.5%)	9 (2.7%)	21 (3.1%)	36 (7.6%)	27 (5.6%)	63 (6.6%)	9 (3.2%)	9 (3.1%)	18 (3.1%)
F8	Spinach	22 (3.1%)	13 (1.7%)	35 (2.3%)	3 (0.9%)	7 (2.1%)	10 (1.5%)	10 (2.1%)	9 (1.9%)	19 (2.0%)	4 (1.4%)	0 (0%)	4 (0.7%)
F9	Cabbage or brussel sprouts	15 (2.1%)	21 (2.7%)	36 (2.4%)	2 (0.6%)	2 (0.6%)	4 (0.6%)	12 (2.5%)	10 (2.1%)	22 (2.3%)	3 (1.1%)	4 (1.4%)	7 (1.2%)
F10	Peas	107 (15.0%)	139 (17.9%)	246 (16.5%)	27 (7.8%)	19 (4.8%)	46 (6.8%)	58 (12.2%)	53 (11.0%)	111 (11.6%)	14 (4.9%)	15 (5.1%)	29 (5.0%)
F11	Broccoli	114 (15.9%)	105 (13.5%)	219 (14.7%)	16 (4.6%)	23 (7.0%)	39 (5.8%)	60 (12.7%)	66 (13.7%)	126 (13.2%)	15 (5.3%)	21 (7.1%)	36 (6.2%)
F12	Carrots	228 (31.9%)	268 (34.5%)	496 (33.3%)	57 (16.5%)	52 (15.8%)	109 (16.1%)	153 (32.3%)	132 (27.4%)	285 (29.8%)	52 (18.4%)	46 (15.6%)	98 (17.0%)
F13	Zucchini, eggplant, squash	38 (5.3%)	49 (6.3%)	87 (5.8%)	3 (0.9%)	7 (2.1%)	10 (1.5%)	16 (3.4%)	17 (3.5%)	33 (3.5%)	4 (1.4%)	7 (2.4%)	11 (1.9%)
F14	Capsicum	39 (5.5%)	49 (6.3%)	88 (5.9%)	4 (1.2%)	3 (0.9%)	7 (1.0%)	35 (7.4%)	22 (4.6%)	57 (6.0%)	7 (2.5%)	2 (0.7%)	9 (1.6%)
F15	Corn, sweetcorn, corn on the cob	104 (14.5%)	117 (15.1%)	221 (14.8%)	27 (7.8%)	23 (7.0%)	50 (7.4%)	57 (12.0%)	45 (9.4%)	102 (10.7%)	11 (3.9%)	22 (7.5%)	33 (5.7%)
F16	Mushrooms	38 (5.3%)	45 (5.8%)	83 (5.6%)	5 (1.4%)	1 (0.3%)	6 (0.9%)	17 (3.6%)	15 (3.1%)	32 (3.4%)	3 (1.1%)	3 (1.0%)	6 (1.0%)
F17	Tomatoes	111 (15.5%)	102 (13.1%)	213 (14.3%)	37 (10.7%)	24 (7.3%)	61 (9.0%)	64 (13.5%)	66 (13.7%)	130 (13.6%)	23 (8.1%)	25 (8.5%)	48 (8.3%)

Table S6a: Percentage of Australian children aged 2–4 years and 5–8 years consuming selected ACAES food categories, in the 2007 ANCNPAS and the 2011–12 NNPAS

		2 – 4-year-old participants						5 – 8-year-old participants					
ACAES FFQ Item		ANCNPAS (2007)			NNPAS (2011–12)			ANCNPAS (2007)			NNPAS (2011–12)		
		Female *n*=715	Male *n*=776	Total *n*=1491	Female *n*=345	Male *n*=330	Total *n*=675	Female *n*=474	Male *n*=481	Total *n*=955	Female *n*=283	Male *n*=295	Total *n*=578
F18	Lettuce	47 (6.6%)	31 (4.0%)	78 (5.2%)	9 (2.6%)	11 (3.3%)	20 (3.0%)	52 (11.0%)	65 (13.5%)	117 (12.3%)	14 (4.9%)	20 (6.8%)	34 (5.9%)
F19	Celery, cucumber	81 (11.3%)	74 (9.5%)	155 (10.4%)	21 (6.1%)	21 (6.4%)	42 (6.2%)	61 (12.9%)	57 (11.9%)	118 (12.4%)	21 (7.4%)	15 (5.1%)	36 (6.2%)
F20	Avocado	33 (4.6%)	24 (3.1%)	57 (3.8%)	8 (2.3%)	11 (3.3%)	19 (2.8%)	12 (2.5%)	10 (2.1%)	22 (2.3%)	5 (1.8%)	3 (1.0%)	8 (1.4%)
F21	Onion, spring onion, leek	98 (13.7%)	124 (16.0%)	222 (14.9%)	5 (1.4%)	12 (3.6%)	17 (2.5%)	61 (12.9%)	60 (12.5%)	121 (12.8%)	7 (2.5%)	5 (1.7%)	12 (2.1%)
F22	Soybeans, tofu	5 (0.7%)	5 (0.6%)	10 (0.7%)	1 (0.3%)	3 (0.9%)	4 (0.6%)	1 (0.2%)	1 (0.2%)	2 (0.2%)	0 (0%)	1 (0.3%)	1 (0.2%)
F23	Baked beans	30 (4.2%)	30 (3.9%)	60 (4.0%)	6 (1.7%)	7 (2.1%)	13 (1.9%)	14 (3%)	14 (2.9%)	28 (2.9%)	2 (0.7%)	6 (2.0%)	8 (1.4%)
F24	Other beans, lentils	18 (2.5%)	10 (1.3%)	28 (1.9%)	4 (1.2%)	4 (1.2%)	8 (1.2%)	7 (1.5%)	9 (1.9%)	16 (1.7%)	2 (0.7%)	1 (0.3%)	3 (0.5%)
F25	Canned fruit	49 (6.9%)	52 (6.7%)	101 (6.8%)	15 (4.3%)	14 (4.2%)	29 (4.3%)	25 (5.3%)	28 (5.8%)	53 (5.5%)	9 (3.2%)	11 (3.7%)	20 (3.5%)
F26	Fruit salad	22 (3.1%)	34 (4.4%)	56 (3.8%)	17 (4.9%)	12 (3.6%)	29 (4.3%)	9 (1.9%)	6 (1.2%)	15 (1.6%)	4 (1.4%)	8 (2.7%)	12 (2.1%)
F27	Dried fruit	99 (13.8%)	133 (17.1%)	232 (15.6%)	32 (9.3%)	40 (12.1%)	72 (10.7%)	28 (5.9%)	32 (6.7%)	60 (6.3%)	11 (3.9%)	15 (5.1%)	26 (4.5%)
F28	Apple or pear	347 (48.5%)	398 (51.3%)	745 (50.0%)	144 (41.7%)	132 (40.0%)	276 (40.9%)	184 (38.8%)	179 (37.2%)	363 (38.0%)	124 (43.8%)	132 (44.7%)	256 (44.3%)
F29	Orange, mandarin, grapefruit	159 (22.2%)	171 (22.0%)	330 (22.1%)	49 (14.2%)	49 (14.8%)	98 (14.5%)	95 (20.0%)	83 (17.3%)	178 (18.6%)	65 (23.0%)	39 (13.2%)	104 (18.0%)
F30	Banana	280 (39.2%)	253 (32.6%)	533 (35.7%)	88 (25.5%)	109 (33.0%)	197 (29.2%)	108 (22.8%)	118 (24.5%)	226 (23.7%)	53 (18.7%)	65 (22.0%)	118 (20.4%)
**Seasonal Fruits:**													

Table S6a: Percentage of Australian children aged 2–4 years and 5–8 years consuming selected ACAES food categories, in the 2007 ANCNPAS and the 2011–12 NNPAS

ACAES FFQ Item		2 – 4-year-old participants						5 – 8-year-old participants					
		ANCNPAS (2007)			NNPAS (2011–12)			ANCNPAS (2007)			NNPAS (2011–12)		
		Female *n*=715	Male *n*=776	Total *n*=1491	Female *n*=345	Male *n*=330	Total *n*=675	Female *n*=474	Male *n*=481	Total *n*=955	Female *n*=283	Male *n*=295	Total *n*=578
SF1	Peach, nectarine, plum or apricot	9 (1.3%)	10 (1.3%)	19 (1.3%)	15 (4.3%)	16 (4.8%)	31 (4.6%)	16 (3.4%)	10 (2.1%)	26 (2.7%)	13 (4.6%)	11 (3.7%)	24 (4.2%)
SF2	Mango or paw-paw	5 (0.7%)	2 (0.3%)	7 (0.5%)	8 (2.3%)	6 (1.8%)	14 (2.1%)	2 (0.4%)	0 (0%)	2 (0.2%)	3 (1.1%)	6 (2.0%)	9 (1.6%)
SF3	Pineapple	13 (1.8%)	6 (0.8%)	19 (1.3%)	3 (0.9%)	4 (1.2%)	7 (1.0%)	2 (0.4%)	5 (1.0%)	7 (0.7%)	2 (0.7%)	3 (1.0%)	5 (0.9%)
SF4	Grapes, strawberries, blueberries	153 (21.4%)	144 (18.6%)	297 (19.9%)	88 (25.5%)	88 (26.7%)	176 (26.1%)	61 (12.9%)	49 (10.2%)	110 (11.5%)	55 (19.4%)	59 (20.0%)	114 (19.7%)
SF5	Melon	64 (9.0%)	92 (11.9%)	156 (10.5%)	30 (8.7%)	33 (10.0%)	63 (9.3%)	45 (9.5%)	40 (8.3%)	85 (8.9%)	26 (9.2%)	24 (8.1%)	50 (8.7%)

Table S6b: Percentage of Australian children aged 9-12 years and 13-16 years consuming selected ACAES food categories, in the 2007 ANCNPAS and the 2011-12 NNPAS

ACAES FFQ Item		9 – 12 year old participants						13 – 16 year old participants					
		ANCNPAS (2007)			NNPAS (2011–12)			ANCNPAS (2007)			NNPAS (2011–12)		
		Female *n*=509	Male *n*=457	Persons *n*=966	Female *n*=308	Male *n*=314	Total *n*=622	Female *n*=694	Male *n*=695	Persons *n*=1389	Female *n*=336	Male *n*=337	Total *n*=673
**Drinks**													
D1	DIET soft drink	28 (5.5%)	24 (5.3%)	52 (5.4%)	17 (5.5%)	20 (6.4%)	37 (5.9%)	53 (7.6%)	69 (9.9%)	122 (8.8%)	22 (6.5%)	22 (6.5%)	44 (6.5%)
D2	Soft drink (NOT DIET) including flavoured mineral water	145 (28.5%)	148 (32.4%)	293 (30.3%)	74 (24.0%)	86 (27.4%)	160 (25.7%)	217 (31.3%)	280 (40.3%)	497 (35.8%)	111 (33.0%)	141 (41.8%)	252 (37.4%)
D3	Water - including bottled water. unflavoured mineral water, tap water	488 (95.9%)	439 (96.1%)	927 (96.0%)	285 (92.5%)	290 (92.4%)	575 (92.4%)	630 (90.8%)	623 (89.6%)	1253 (90.2%)	300 (89.3%)	298 (88.4%)	598 (88.9%)
D4	Fruit juice-based drinks	230 (45.2%)	203 (44.4%)	433 (44.8%)	124 (40.3%)	119 (37.9%)	243 (39.1%)	317 (45.7%)	289 (41.6%)	606 (43.6%)	119 (35.4%)	120 (35.6%)	239 (35.5%)
D5	Cordial or 'make up'	105 (20.6%)	104 (22.8%)	209 (21.6%)	31 (10.1%)	44 (14.0%)	75 (12.1%)	124 (17.9%)	154 (22.2%)	278 (20.0%)	30 (8.9%)	42 (12.5%)	72 (10.7%)
D6	Tea or Coffee	41 (8.1%)	27 (5.9%)	68 (7.0%)	21 (6.8%)	16 (5.1%)	37 (5.9%)	133 (19.2%)	118 (17%)	251 (18.1%)	75 (22.3%)	46 (13.6%)	121 (18.0%)
D7	Beer	0 (0%)	0 (0%)	0 (0%)	0 (0%)	0 (0%)	0 (0%)	0 (0%)	1 (0.1%)	1 (0.1%)	0 (0%)	2 (0.6%)	2 (0.3%)
D8	Wine or wine coolers	4 (0.8%)	2 (0.4%)	6 (0.6%)	0 (0%)	0 (0%)	0 (0%)	7 (1%)	7 (1%)	14 (1%)	1 (0.3%)	2 (0.6%)	3 (0.4%)
D9	Spirits	1 (0.2%)	0 (0%)	1 (0.1%)	0 (0%)	0 (0%)	0 (0%)	7 (1%)	2 (0.3%)	9 (0.6%)	0 (0%)	1 (0.3%)	1 (0.1%)
**Dairy foods:**													
DF1 + DF3	Soy Milk	6 (1.2%)	7 (1.5%)	13 (1.3%)	2 (0.6%)	1 (0.3%)	3 (0.5%)	12 (1.7%)	7 (1.0%)	19 (1.4%)	3 (0.9%)	3 (0.9%)	6 (0.9%)
DF1 + DF3	Whole Milk	209 (41.2%)	221 (48.4%)	430 (44.5%)	117 (33.9%)	145 (46.2%)	262 (42.1%)	241 (34.7%)	337 (48.5%)	578 (41.6%)	103 (30.7%)	146 (43.3%)	249 (37.0%)

Table S6b: Percentage of Australian children aged 9-12 years and 13-16 years consuming selected ACAES food categories, in the 2007 ANCNPAS and the 2011-12 NNPAS

		9 – 12 year old participants						13 – 16 year old participants					
ACAES FFQ Item		ANCNPAS (2007)			NNPAS (2011–12)			ANCNPAS (2007)			NNPAS (2011–12)		
		Female *n*=509	Male *n*=457	Persons *n*=966	Female *n*=308	Male *n*=314	Total *n*=622	Female *n*=694	Male *n*=695	Persons *n*=1389	Female *n*=336	Male *n*=337	Total *n*=673
DF1 + DF3	Reduced Fat Milk	120 (23.6%)	117 (48.4%)	237 (24.5%)	57 (18.5%)	60 (19.1%)	117 (18.8%)	133 (19.2%)	145 (20.9%)	278 (20.0%)	66 (19.6%)	53 (15.7%)	119 (17.7%)
DF1 + DF3	Skim Milk	32 (6.3%)	17 (3.7%)	49 (5.1%)	17 (5.5%)	9 (2.9%)	26 (4.2%)	36 (5.2%)	23 (3.3%)	59 (4.2%)	23 (6.8%)	9 (2.7%)	32 (4.8%)
DF2	Flavoured milk	83 (16.3%)	75 (16.4%)	158 (16.4%)	30 (9.7%)	35 (11.1%)	65 (10.5%)	126 (18.2%)	118 (17.0%)	254 (18.3%)	44 (13.1%)	46 (13.6%)	90 (13.4%)
DF4	Cream or sour cream	26 (5.1%)	28 (6.1%)	54 (5.6%)	7 (2.3%)	4 (1.3%)	11 (1.8%)	46 (6.6%)	32 (4.6%)	78 (5.6%)	7 (2.1%)	12 (3.7%)	19 (2.8%)
DF5	Ice cream	116 (22.8%)	97 (21.2%)	213 (22.0%)	56 (18.2%)	51 (16.2%)	107 (17.2%)	100 (14.4%)	127 (18.3%)	227 (16.3%)	47 (14.0%)	54 (16.0%)	101 (15.0%)
DF6	Frozen yoghurt	4 (0.8%)	5 (1.1%)	9 (0.9%)	4 (1.3%)	4 (1.3%)	8 (1.3%)	6 (0.9%)	4 (0.6%)	10 (0.7%)	1 (0.3%)	2 (0.6%)	3 (0.4%)
DF7	Yoghurt (not frozen) plain or flavoured	73 (14.3%)	65 (14.2%)	138 (14.3%)	45 (14.6%)	39 (12.4%)	84 (13.5%)	94 (13.5%)	74 (10.6%)	168 (12.1%)	39 (11.6%)	28 (8.3%)	67 (10.0%)
DF8	Cottage cheese or ricotta	2 (0.4%)	1 (0.2%)	3 (0.3%)	1 (0.3%)	3 (1.0%)	4 (0.6%)	3 (0.4%)	1 (0.1%)	4 (0.3%)	2 (0.6%)	0 (0%)	2 (0.3%)
DF9	Cheese including cheese on sandwiches, biscuits or on toast	208 (40.9%)	166 (36.3%)	374 (38.7%)	66 (21.4%)	77 (24.5%)	143 (23.0%)	256 (36.9%)	245 35.3%)	501 (36.1%)	48 (14.3%)	79 (23.4%)	127 (18.9%)
DF10	Cheese spread, cream cheese	15 (2.9%)	10 (2.2%)	25 (2.6%)	6 (1.9%)	7 (2.2%)	13 (2.1%)	16 (2.3%)	20 (2.9%)	36 (2.6%)	11 (3.3%)	4 (1.2%)	15 (2.2%)
**Breads and Cereal:**													
B1	Muesli	11 (2.2%)	3 (0.7%)	14 (1.4%)	3 (0.9%)	1 (0.3%)	4 (0.6%)	8 (1.2%)	9 (1.3%)	17 (1.2%)	8 (2.4%)	4 (1.2%)	12 (1.8%)
B2	Cooked porridge	23 (4.5%)	26 (5.7%)	49 (5.1%)	21 (6.8%)	5 (1.6%)	26 (4.2%)	30 (4.3%)	20 (2.9%)	50 (3.6%)	14 (4.2%)	12 (3.6%)	26 (3.9%)

Table S6b: Percentage of Australian children aged 9-12 years and 13-16 years consuming selected ACAES food categories, in the 2007 ANCNPAS and the 2011-12 NNPAS

ACAES FFQ Item		9 – 12 year old participants						13 – 16 year old participants					
		ANCNPAS (2007)			NNPAS (2011–12)			ANCNPAS (2007)			NNPAS (2011–12)		
		Female *n*=509	Male *n*=457	Persons *n*=966	Female *n*=308	Male *n*=314	Total *n*=622	Female *n*=694	Male *n*=695	Persons *n*=1389	Female *n*=336	Male *n*=337	Total *n*=673
B3	Breakfast cereal	191 (37.5%)	247 (54.0%)	438 (45.3%)	109 (35.4%)	139 (44.3%)	248 (39.9%)	205 (29.5%)	357 (51.4%)	562 (40.5%)	89 (26.5%)	141 (41.8%)	230 (34.2%)
B5	Bread, pita bread, roll – White	284 (55.8%)	266 (58.2%)	550 (57.0%)	167 (54.2%)	178 (56.7%)	345 (55.5%)	342 (49.3%)	391 (56.3%)	733 (52.8%)	141 (42.0%)	185 (54.9%)	326 (48.5%)
B5	Bread, pita bread, roll – Mixed Grain	51 (10.0%)	32 (7.0%)	83 (8.6%)	21 (6.8%)	21 (6.7%)	42 (6.8%)	80 (11.5%)	61 (8.8%)	141 (10.2%)	31 (9.2%)	20 (5.9%)	51 (7.6%)
B5	Bread, pita bread, roll - Wholemeal	73 (14.3%)	72 (15.8%)	145 (15.0%)	36 (10.4%)	42 (13.4%)	78 (12.5%)	106 (15.3%)	91 (13.1%)	197 (14.2%)	49 (14.6%)	31 (9.2%)	80 (11.9%)
B6	English muffin, bagel or crumpet	25 (4.9%)	21 (4.6%)	46 (4.8%)	8 (2.6%)	11 (3.5%)	19 (3.1%)	35 (5.0%)	42 (6.0%)	77 (5.5%)	14 (4.2%)	7 (2.1%)	21 (3.1%)
B7	Rice	80 (15.7%)	65 (14.2%)	145 (15.0%)	60 (19.5%)	56 (17.8%)	116 (18.6%)	108 (15.6%)	121 (17.4%)	229 (16.5%)	50 (14.9%)	55 (16.3%)	105 (15.6%)
B8	Other grains eg. cous cous, burghul	3 (0.6%)	2 (0.4%)	5 (0.5%)	5 (1.6%)	4 (1.3%)	9 (1.4%)	4 (0.6%)	3 (0.4%)	7 (0.5%)	4 (1.2%)	1 (0.3%)	5 (0.7%)
B9	Noodles	11 (2.2%)	9 (2.0%)	20 (2.1%)	10 (3.2%)	7 (2.2%)	17 (2.7%)	14 (2.0%)	11 (1.6%)	25 (1.8%)	8 (2.4%)	8 (2.4%)	16 (2.4%)
B10	Pasta	106 (20.8%)	98 (21.4%)	204 (21.1%)	34 (11.0%)	42 (13.4%)	76 (12.2%)	142 (20.5%)	151 (21.7%)	293 (21.1%)	38 (11.3%)	40 (11.9%)	78 (11.6%)
**Sweets and Snacks:**													
S1	Cakes, sweet muffins, scones, pikelets, pancakes, hot cakes	145 (28.5%)	113 (24.7%)	258 (26.7%)	71 (23.1%)	69 (22.0%)	140 (22.5%)	180 (25.9%)	167 (24.0%)	347 (25.0%)	74 (22.0%)	60 (17.8%)	134 (19.9%)
S2	Sweet pies or sweet pastries	46 (9.0%)	39 (8.5%)	85 (8.8%)	21 (6.8%)	23 (7.3%)	44 (7.1%)	42 (6.1%)	51 (7.3%)	93 (6.7%)	23 (6.8%)	30 (8.9%)	53 (7.9%)

Table S6b: Percentage of Australian children aged 9-12 years and 13-16 years consuming selected ACAES food categories, in the 2007 ANCNPAS and the 2011-12 NNPAS

		9 – 12 year old participants						13 – 16 year old participants					
ACAES FFQ Item		ANCNPAS (2007)			NNPAS (2011–12)			ANCNPAS (2007)			NNPAS (2011–12)		
		**Female** *n*=509	**Male** *n*=457	**Persons** *n*=966	**Female** *n*=308	**Male** *n*=314	**Total** *n*=622	**Female** *n*=694	**Male** *n*=695	**Persons** *n*=1389	**Female** *n*=336	**Male** *n*=337	**Total** *n*=673
S3	Other puddings or desserts (not ice cream)	43 (8.4%)	32 (7.0%)	75 (7.8%)	24 (7.8%)	15 (4.8%)	39 (6.3%)	43 (6.2%)	35 (5.0%)	78 (5.6%)	18 (5.4%)	18 (5.3%)	36 (5.3%)
S4	Plain sweet biscuits	81 (15.9%)	69 (15.1%)	150 (15.5%)	61 (19.8%)	61 (19.4%)	122 (19.6%)	92 (13.3%)	92 (13.2%)	184 (13.2%)	47 (14.0%)	44 (13.1%)	91 (13.5%)
S5	Cream or chocolate biscuits	64 (12.6%)	75 (16.4%)	139 (14.4%)	36 (11.7%)	38 (12.1%)	74 (11.9%)	78 (11.2%)	100 (14.4%)	178 (12.8%)	32 (9.5%)	33 (9.8%)	65 (9.7%)
S6	Dry or savoury biscuits, crispbread, crackers	134 (26.3%)	106 (23.2%)	240 (24.8%)	68 (22.1%)	71 (22.6%)	139 (22.3%)	124 (17.9%)	109 (15.7%)	233 (16.8%)	69 (20.5%)	50 (14.8%)	119 (17.7%)
S7	Savoury combination snacks	15 (2.9%)	12 (2.6%)	27 (2.8%)	6 (1.9%)	10 (3.2%)	16 (2.6%)	11 (1.6%)	13 (1.9%)	24 (1.7%)	8 (2.4%)	3 (0.9%)	11 (1.6%)
S8	Sweet combination snacks	-	-	-	-	-	-	-	-	-	-	-	-
S9	Snack noodles	33 (6.5%)	38 (8.3%)	71 (7.3%)	17 (5.5%)	17 (5.4%)	34 (5.5%)	52 (7.5%)	48 (6.9%)	100 (7.2%)	16 (4.8%)	23 (6.8%)	39 (5.8%)
S10	Fruit bars	0 (0%)	0 (0%)	0 (0%)	4 (1.3%)	2 (0.6%)	6 (1.0%)	0 (0%)	0 (0%)	0 (0%)	4 (1.2%)	1 (0.3%)	5 (0.7%)
S11	Snack bars	19 (3.7%)	18 (3.9%)	37 (3.8%)	1 (0.3%)	4 (1.3%)	5 (0.8%)	26 (3.7%)	32 (4.6%)	58 (4.2%)	0 (0%)	3 (0.9%)	3 (0.4%)
S12	Muesli bars	63 (12.4%)	60 (13.1%)	123 (12.7%)	47 (15.3%)	53 (16.9%)	100 (16.1%)	97 (14.0%)	114 (16.4%)	211 (15.2%)	48 (14.3%)	59 (17.5%)	107 (15.9%)
**Main Meals:**													
M1	Mince dish	63 (12.4%)	69 (15.1%)	132 (13.7%)	39 (12.7%)	41 (13.1%)	80 (12.9%)	84 (12.1%)	99 (14.2%)	183 (13.2%)	49 (14.6%)	44 (13.1%)	93 (13.8%)

Table S6b: Percentage of Australian children aged 9-12 years and 13-16 years consuming selected ACAES food categories, in the 2007 ANCNPAS and the 2011-12 NNPAS

ACAES FFQ Item		9 – 12 year old participants						13 – 16 year old participants					
		ANCNPAS (2007)			NNPAS (2011–12)			ANCNPAS (2007)			NNPAS (2011–12)		
		Female *n*=509	Male *n*=457	Persons *n*=966	Female *n*=308	Male *n*=314	Total *n*=622	Female *n*=694	Male *n*=695	Persons *n*=1389	Female *n*=336	Male *n*=337	Total *n*=673
M2-3, M5, M11–12, 14	Mixed meat dish	12 (2.4%)	8 (1.8%)	20 (2.0%)	18 (5.8%)	21 (6.7%)	39 (6.3%)	22 (3.2%)	18 (2.6%)	40 (2.9%)	20 (6.0%)	19 (5.6%)	39 (5.8%)
M4	Plain meat (beef or lamb) WITHOUT vegetables or salad	84 (16.5%)	91 (19.9%)	175 (18.1%)	32 (10.4%)	33 (10.5%)	65 (10.5%)	129 (18.6%)	148 (21.3%)	277 (19.9%)	51 (15.2%)	52 (15.4%)	103 (15.3%)
M6-7, M10	Mixed chicken dish	30 (5.9%)	20 (4.4%)	50 (5.2%)	23 (7.5%)	23 (7.3%)	46 (7.4%)	39 (5.6%)	58 (8.3%)	97 (6.9%)	14 (4.2%)	18 (5.3%)	32 (4.8%)
M8	Chicken crumbed	56 (11.0%)	41 (9.0%)	97 (10.0%)	34 (11.0%)	31 (10.0%)	65 (10.5%)	59 (8.5%)	69 (9.9%)	128 (9.2%)	29 (8.6%)	36 (10.7%)	65 (9.7%)
M9	Plain chicken WITHOUT vegetables	85 (16.7%)	89 (19.5%)	174 (18.0%)	34 (11.0%)	19 (6.1%)	53 (8.5%)	119 (17.1%)	137 (19.7%)	256 (18.4%)	43 (12.8%)	39 (11.6%)	82 (12.2%)
M13	Plain pork WITHOUT vegetables	16 (3.1%)	18 (3.9%)	34 (3.5%)	10 (3.2%)	11 (3.5%)	21 (3.4%)	29 (4.2%)	21 (3.0%)	50 (3.6%)	16 (4.8%)	8 (2.4%)	24 (3.6%)
M15	Liver – beef, calf, chicken	3 (0.6%)	0 (0%)	3 (0.3%)	0 (0%)	0 (0%)	0 (0%)	1 (0.1%)	1 (0.1%)	2 (0.1%)	1 (0.3%)	0 (0%)	1 (0.1%)
M16-17	Fish	28 (5.5%)	27 (5.9%)	55 (5.7%)	21 (6.8%)	15 (4.8%)	36 (5.8%)	31 (4.5%)	13 (1.9%)	44 (3.2%)	9 (2.7%)	18 (5.3%)	27 (4.0%)
M18	Canned tuna, salmon, sardines including patties	25 (4.9%)	16 (3.5%)	41 (4.2%)	8 (2.6%)	12 (3.8%)	20 (3.2%)	26 (3.7%)	20 (2.9%)	46 (3.3%)	9 (2.7%)	9 (2.7%)	18 (2.7%)
M19	Other seafood	9 (1.8%)	9 (2.0%)	18 (1.9%)	3 (1.0%)	4 (1.3%)	7 (1.1%)	12 (1.7%)	9 (1.3%)	21 (1.5%)	4 (1.2%)	5 (1.5%)	9 (1.3%)
M20-21	Creamy or Clear soup	44 (8.6%)	29 (6.3%)	73 (7.6%)	21 (6.8%)	15 (4.8%)	36 (5.8%)	71 (10.2%)	55 (7.9%)	126 (9.1%)	15 (4.5%)	13 (3.9%)	28 (4.2%)
M22	Tacos, burritos, enchiladas	15 (2.9%)	13 (2.8%)	28 (2.9%)	8 (2.6%)	6 (1.9%)	14 (2.3%)	8 (1.2%)	8 (1.2%)	16 (1.2%)	3 (0.9%)	9 (2.8%)	12 (1.8%)

Table S6b: Percentage of Australian children aged 9-12 years and 13-16 years consuming selected ACAES food categories, in the 2007 ANCNPAS and the 2011-12 NNPAS

ACAES FFQ Item		9 – 12 year old participants						13 – 16 year old participants					
		ANCNPAS (2007)			NNPAS (2011–12)			ANCNPAS (2007)			NNPAS (2011–12)		
		Female *n*=509	Male *n*=457	Persons *n*=966	Female *n*=308	Male *n*=314	Total *n*=622	Female *n*=694	Male *n*=695	Persons *n*=1389	Female *n*=336	Male *n*=337	Total *n*=673
M23	Sausages, frankfurts, Pluto Pup	62 (12.2%)	68 (14.9%)	130 (13.5%)	24 (7.8%)	32 (10.2%)	56 (9.0%)	59 (8.5%)	93 (13.4%)	152 (10.9%)	24 (7.1%)	29 (8.6%)	53 (7.9%)
M24	Hamburger - all types	21 (4.1%)	31 (6.8%)	52 (5.4%)	19 (6.2%)	21 (6.7%)	40 (6.4%)	45 (6.5%)	41 (5.9%)	86 (6.2%)	37 (11.0%)	57 (16.9%)	94 (14.0%)
M25	Pizza	40 (7.9%)	35 (7.7%)	75 (7.8%)	18 (5.8%)	23 (7.3%)	41 (6.6%)	54 (7.8%)	73 (10.5%)	127 (9.1%)	28 (8.3%)	36 (10.7%)	64 (9.5%)
M26	Pie, sausage roll, chiko roll	49 (9.6%)	57 (12.5%)	106 (11.0%)	18 (5.8%)	30 (9.6%)	48 (7.7%)	68 (9.8%)	89 (12.8%)	157 (11.3%)	26 (7.7%)	42 (12.5%)	68 (10.1%)
M27	Hot dog	-	-	-	7 (2.3%)	12 (3.8%)	19 (3.1%)	-	-	-	4 (1.2%)	11 (3.3%)	15 (2.2%)
M28	Savoury pastries	28 (5.5%)	16 (3.5%)	44 (4.6%)	3 (1.0%)	3 (1.0%)	6 (1.0%)	35 (5.0%)	18 (2.6%)	53 (3.8%)	13 (3.9%)	7 (2.1%)	20 (3.0%)
M29	Hash browns, potato scallops	16 (3.1%)	7 (1.5%)	23 (2.4%)	4 (1.3%)	7 (2.2%)	11 (1.8%)	11 (1.6%)	19 (2.7%)	30 (2.2%)	7 (2.1%)	12 (3.6%)	19 (2.8%)
**Other Foods:**													
O1	Chips (not potato)	59 (11.6%)	54 (11.8%)	113 (11.7%)	46 (14.9%)	42 (13.4%)	88 (14.1%)	66 (9.5%)	52 (7.5%)	118 (8.5%)	33 (9.8%)	31 (9.2%)	64 (9.5%)
O2	Potato chips or crisps	95 (18.7%)	98 (21.4%)	193 (20%)	57 (18.5%)	64 (20.4%)	121 (19.5%)	125 (18.0%)	134 (19.2%)	259 (18.6%)	42 (12.5%)	55 (16.3%)	97 (14.4%)
O3	Ice block – creamy	28 (5.5%)	29 (6.3%)	57 (5.9%)	18 (5.8%)	21 (6.7%)	39 (6.3%)	36 (5.2%)	31 (4.5%)	67 (4.8%)	15 (4.5%)	11 (3.3%)	26 (3.9%)
O4	Ice block – water	29 (5.7%)	29 (6.3%)	58 (6.0%)	32 (10.4%)	29 (9.2%)	61 (9.8%)	25 (3.6%)	24 (3.5%)	49 (3.5%)	15 (4.5%)	10 (3.0%)	25 (3.7%)
O5	Chocolate	148 (29.1%)	129 (28.2%)	277 (28.7%)	67 (21.8%)	60 (19.1%)	127 (20.4%)	186 (26.8%)	145 (20.9%)	331 (23.8%)	72 (21.4%)	59 (17.5%)	131 (19.5%)

Table S6b: Percentage of Australian children aged 9-12 years and 13-16 years consuming selected ACAES food categories, in the 2007 ANCNPAS and the 2011-12 NNPAS

		9 – 12 year old participants						13 – 16 year old participants					
ACAES FFQ Item		ANCNPAS (2007)			NNPAS (2011–12)			ANCNPAS (2007)			NNPAS (2011–12)		
		Female *n*=509	Male *n*=457	Persons *n*=966	Female *n*=308	Male *n*=314	Total *n*=622	Female *n*=694	Male *n*=695	Persons *n*=1389	Female *n*=336	Male *n*=337	Total *n*=673
O6	Lollies without chocolate	107 (21.0%)	102 (22.3%)	209 (21.6%)	54 (17.5%)	52 (16.6%)	106 (17.0%)	141 (20.3%)	121 (17.4%)	262 (18.9%)	54 (16.1%)	33 (9.8%)	87 (12.9%)
O7	Low fat salad dressing or mayonnaise	13 (2.6%)	5 (1.1%)	18 (1.9%)	4 (1.3%)	3 (1.0%)	7 (1.1%)	20 (2.9%)	28 (4.0%)	48 (3.5%)	5 (1.5%)	5 (1.5%)	10 (1.5%)
O8	Salad dressing or mayonnaise - not low fat	39 (7.7%)	39 (8.5%)	78 (8.1%)	27 (8.8%)	26 (8.3%)	53 (8.5%)	68 (9.8%)	74 (10.6%)	142 (10.2%)	26 (7.7%)	37 (11.0%)	63 (9.4%)
O9	Nuts	24 (4.7%)	16 (3.5%)	40 (4.1%)	7 (2.3%)	7 (2.2%)	14 (2.3%)	36 (5.2%)	25 (3.6%)	61 (4.4%)	15 (4.5%)	9 (2.7%)	24 (3.6%)
O10	Jam, honey, golden syrup, marmalade	90 (17.7%)	73 (16.0%)	163 (16.9%)	35 (11.4%)	38 (12.1%)	73 (11.7%)	123 (17.7%)	111 (16.0%)	234 (16.8%)	40 (11.9%)	44 (13.1%)	84 (12.5%)
O11	Peanut butter, Nutella	67 (13.2%)	76 (16.6%)	143 (14.8%)	30 (9.7%)	36 (11.5%)	66 (10.6%)	80 (11.5%)	102 (14.7%)	182 (13.1%)	34 (10.1%)	41 (12.2%)	75 (11.1%)
O12	Vegemite, Mighty Mite, Promite, Marmite	111 (21.8%)	76 (16.6%)	187 (19.4%)	54 (17.5%)	72 (22.9%)	126 (20.3%)	141 (20.3%)	97 (14.0%)	238 (17.1%)	42 (12.5%)	42 (12.5%)	84 (12.5%)
O13	Tomato sauce, barbecue sauce	131 (25.7%)	139 (30.4%)	270 (30.0%)	44 (14.3%)	49 (15.6%)	93 (15.0%)	159 (22.9%)	204 (29.4%)	363 (26.1%)	51 (15.2%)	68 (20.2%)	119 (17.7%)
O14	Devon, salami	30 (5.9%)	33 (7.2%)	63 (6.5%)	15 (4.9%)	18 (5.1%)	33 (5.3%)	37 (5.3%)	50 (7.2%)	87 (6.2%)	5 (1.5%)	9 (2.7%)	14 (2.1%)
O15	Bacon, ham	93 (18.3%)	90 (19.7%)	183 (18.9%)	64 (20.8%)	61 (19.4%)	125 (20.1%)	145 (20.9%)	156 (22.4%)	301 (21.7%)	67 (19.9%)	71 (21.1%)	138 (20.5%)
O16	Eggs	58 (11.4%)	60 (13.1%)	118 (12.2%)	38 (12.3%)	28 (8.9%)	66 (10.6%)	101 (14.6%)	86 (12.4%)	187 (13.5%)	42 (12.5%)	40 (11.9%)	82 (12.2%)
O17	Jelly	23 (4.5%)	9 (2.0%)	32 (3.3%)	11 (3.6%)	6 (1.9%)	17 (2.7%)	10 (1.4%)	6 (0.9%)	16 (1.2%)	6 (1.8%)	4 (1.2%)	10 (1.5%)
**Fruit and Vegetables**													

Table S6b: Percentage of Australian children aged 9-12 years and 13-16 years consuming selected ACAES food categories, in the 2007 ANCNPAS and the 2011-12 NNPAS

		9 – 12 year old participants						13 – 16 year old participants					
ACAES FFQ Item		ANCNPAS (2007)			NNPAS (2011–12)			ANCNPAS (2007)			NNPAS (2011–12)		
		Female *n*=509	Male *n*=457	Persons *n*=966	Female *n*=308	Male *n*=314	Total *n*=622	Female *n*=694	Male *n*=695	Persons *n*=1389	Female *n*=336	Male *n*=337	Total *n*=673
F1-2	Hot chips bought from a shop or cooked at home	104 (20.4%)	94 (20.6%)	198 (20.5%)	58 (18.8%)	54 (17.2%)	112 (18.0%)	167 (24.1%)	154 (22.2%)	321 (23.1%)	60 (17.9%)	58 (17.2%)	118 (17.5%)
F3	Potato boiled, mashed, baked	125 (24.6%)	115 (25.2%)	240 (24.8%)	45 (14.6%)	52 (16.6%)	97 (15.6%)	158 (22.8%)	158 (22.7%)	316 (22.8%)	63 (18.8%)	46 (13.6%)	109 (16.2%)
F4	Pumpkin	38 (7.5%)	25 (5.5%)	63 (6.5%)	5 (1.6%)	12 (3.8%)	17 (2.7%)	50 (7.2%)	27 (3.9%)	77 (5.5%)	10 (3.0%)	7 (2.1%)	17 (2.5%)
F5	Sweet potato	11 (2.2%)	16 (3.5%)	27 (2.8%)	7 (2.3%)	6 (1.9%)	13 (2.1%)	22 (3.2%)	20 (2.9%)	42 (3.0%)	2 (0.6%)	6 (1.8%)	8 (1.2%)
F6	Cauliflower	28 (5.5%)	27 (5.9%)	55 (5.7%)	5 (1.6%)	8 (2.5%)	13 (2.1%)	37 (5.3%)	30 (4.3%)	67 (4.8%)	6 (1.8%)	7 (2.1%)	13 (1.9%)
F7	Green beans	51 (10.0%)	32 (7.0%)	83 (8.6%)	11 (3.6%)	6 (1.9%)	17 (2.7%)	61 (8.8%)	59 (8.5%)	120 (8.6%)	11 (3.3%)	14 (4.2%)	25 (3.7%)
F8	Spinach	13 (2.6%)	8 (1.8%)	21 (2.2%)	10 (3.2%)	4 (1.3%)	14 (2.3%)	21 (3.0%)	25 (3.6%)	46 (3.3%)	10 (3.0%)	2 (0.6%)	12 (1.8%)
F9	Cabbage or brussel sprouts	12 (2.4%)	15 (3.3%)	27 (2.8%)	1 (0.3%)	0 (0%)	1 (0.2%)	23 (3.3%)	26 (3.7%)	49 (3.5%)	2 (0.6%)	4 (1.2%)	6 (0.9%)
F10	Peas	69 (13.6%)	56 (12.3%)	125 (12.9%)	13 (4.2%)	15 (4.8%)	28 (4.5%)	80 (11.5%)	78 (11.2%)	168 (12.1%)	14 (4.2%)	17 (5.0%)	31 (4.6%)
F11	Broccoli	72 (14.1%)	57 (12.5%)	129 (13.4%)	14 (4.5%)	17 (5.4%)	31 (5.0%)	87 (12.5%)	81 (11.7%)	168 (12.1%)	17 (5.1%)	14 (4.2%)	31 (4.6%)
F12	Carrots	170 (33.4%)	136 (30.0%)	306 (31.7%)	44 (14.3%)	42 (13.4%)	86 (13.8%)	203 (29.3%)	181 (26.0%)	384 (27.6%)	40 (11.9%)	44 (13.1%)	84 (12.5%)
F13	Zucchini, eggplant, squash	21 (4.1%)	16 (3.5%)	37 (3.8%)	3 (1.0%)	3 (1.0%)	6 (1.0%)	24 (3.5%)	18 (2.6%)	42 (3.0%)	1 (0.3%)	4 (1.2%)	5 (0.7%)
F14	Capsicum	41 (8.1%)	35 (7.7%)	76 (7.9%)	6 (1.9%)	4 (1.3%)	10 (1.6%)	55 (7.9%)	49 (7.1%)	104 (7.5%)	7 (2.1%)	4 (1.2%)	11 (1.6%)
F15	Corn, sweetcorn, corn on the cob	53 (10.4%)	51 (11.2%)	104 (10.8%)	10 (3.2%)	15 (4.8%)	25 (4.0%)	58 (8.4%)	54 (7.8%)	112 (8.1%)	15 (4.5%)	15 (4.5%)	30 (4.5%)

Table S6b: Percentage of Australian children aged 9-12 years and 13-16 years consuming selected ACAES food categories, in the 2007 ANCNPAS and the 2011-12 NNPAS

		9 – 12 year old participants						13 – 16 year old participants					
ACAES FFQ Item		ANCNPAS (2007)			NNPAS (2011–12)			ANCNPAS (2007)			NNPAS (2011–12)		
		Female *n*=509	Male *n*=457	Persons *n*=966	Female *n*=308	Male *n*=314	Total *n*=622	Female *n*=694	Male *n*=695	Persons *n*=1389	Female *n*=336	Male *n*=337	Total *n*=673
F16	Mushrooms	22 (4.3%)	16 (3.5%)	38 (3.9%)	2 (0.6%)	5 (1.6%)	7 (1.1%)	41 (5.9%)	25 (3.6%)	66 (4.8%)	3 (0.9%)	5 (1.5%)	8 (1.2%)
F17	Tomatoes	111 (21.8%)	79 (17.3%)	190 (19.7%)	37 (12.0%)	30 (9.6%)	67 (10.8%)	135 (19.5%)	142 (20.4%)	277 (19.9%)	39 (11.6%)	32 (9.5%)	71 (10.5%)
F18	Lettuce	92 (18.1%)	67 (14.7%)	159 (16.5%)	34 (11.0%)	28 (8.9%)	62 (10.0%)	122 17.6%)	134 (19.3%)	156 (11.2%)	31 (9.2%)	48 (14.2%)	79 (11.7%)
F19	Celery, cucumber	68 (13.4%)	47 (10.3%)	115 (11.9%)	29 (9.4%)	15 (4.8%)	44 (7.1%)	94 (13.5%)	76 (10.9%)	170 (12.2%)	20 (6.0%)	23 (6.8%)	43 (6.4%)
F20	Avocado	17 (3.3%)	12 (2.6%)	29 (3.0%)	8 (2.6%)	4 (1.3%)	12 (1.9%)	33 (4.8%)	19 (2.7%)	52 (3.7%)	8 (2.4%)	2 (0.6%)	10 (1.5%)
F21	Onion, spring onion, leek	78 (15.3%)	72 (15.8%)	150 (15.5%)	12 (3.9%)	12 (3.8%)	24 (3.9%)	138 (19.9%)	165 (23.7%)	303 (21.8%)	21 (6.3%)	13 (3.9%)	34 (5.1%)
F22	Soybeans, tofu	2 (0.4%)	3 (0.7%)	5 (0.5%)	1 (0.3%)	1 (0.3%)	2 (0.3%)	5 (0.7%)	7 (1.0%)	12 (0.9%)	3 (0.9%)	0 (0%)	3 (0.4%)
F23	Baked beans	6 (1.2%)	10 (2.2%)	16 (1.7%)	5 (1.6%)	6 (1.9%)	11 (1.8%)	12 (1.7%)	12 (1.7%)	24 (1.7%)	3 (0.9%)	7 (2.1%)	10 (1.5%)
F24	Other beans, lentils	7 (1.4%)	11 (2.4%)	18 (1.9%)	2 (0.6%)	0 (0%)	2 (0.3%)	19 (2.7%)	12 (1.7%)	31 (2.2%)	6 (1.8%)	2 (0.6%)	8 (1.2%)
F25	Canned fruit	24 (4.7%)	18 (3.9%)	42 (4.3%)	9 (2.9%)	3 (1.0%)	12 (1.9%)	22 (3.2%)	19 (2.7%)	41 (3.0%)	6 (1.8%)	5 (1.5%)	11 (1.6%)
F26	Fruit salad	9 (1.8%)	8 (1.8%)	17 (1.8%)	10 (3.2%)	2 (0.6%)	12 (1.9%)	11 (1.6%)	11 (1.6%)	22 (1.6%)	11 (3.3%)	2 (0.6%)	13 (1.9%)
F27	Dried fruit	19 (3.7%)	18 (3.9%)	37 (3.8%)	8 (2.6%)	6 (1.9%)	14 (2.3%)	36 (5.2%)	28 (4.0%)	64 (4.6%)	9 (2.7%)	3 (0.9%)	12 (1.8%)
F28	Apple or pear	198 (38.9%)	152 (33.3%)	350 (36.2%)	97 (31.5%)	122 (38.9%)	219 (35.2%)	198 (28.5%)	173 (24.9%)	371 (26.7%)	77 (22.9%)	89 (26.4%)	166 (24.7%)
F29	Orange, mandarin, grapefruit	84 (16.5%)	66 (14.4%)	150 (15.5%)	55 (17.9%)	45 (14.3%)	100 (16.1%)	125 (18.0%)	101 (14.5%)	226 (16.3%)	41 (12.2%)	36 (10.7%)	77 (11.4%)

Table S6b: Percentage of Australian children aged 9-12 years and 13-16 years consuming selected ACAES food categories, in the 2007 ANCNPAS and the 2011-12 NNPAS

ACAES FFQ Item		9 – 12 year old participants						13 – 16 year old participants					
		ANCNPAS (2007)			NNPAS (2011–12)			ANCNPAS (2007)			NNPAS (2011–12)		
		Female *n*=509	Male *n*=457	Persons *n*=966	Female *n*=308	Male *n*=314	Total *n*=622	Female *n*=694	Male *n*=695	Persons *n*=1389	Female *n*=336	Male *n*=337	Total *n*=673
F30	Banana	95 (18.7%)	77 (16.8%)	172 (17.8%)	46 (14.9%)	39 (12.4%)	85 (13.7%)	87 (12.5%)	97 (14.0%)	184 (13.2%)	33 (9.8%)	31 (9.2%)	64 (9.5%)
**Seasonal Fruits:**													
SF1	Peach, nectarine, plum or apricot	14 (2.8%)	14 (3.1%)	28 (2.9%)	26 (8.4%)	13 (4.1%)	39 (6.3%)	21 (3.0%)	9 (1.3%)	30 (2.2%)	23 (6.8%)	16 (4.7%)	39 (5.8%)
SF2	Mango or Paw-paw	0 (0%)	7 (1.5%)	7 (0.7%)	8 (2.6%)	7 (2.2%)	15 (2.4%)	0 (0%)	2 (0.3%)	2 (0.1%)	7 (2.1%)	1 (0.3%)	8 (1.2%)
SF3	Pineapple	9 (1.8%)	6 (1.3%)	15 (1.6%)	5 (1.6%)	0 (0%)	5 (0.8%)	11 (1.6%)	8 (1.2%)	19 (1.4%)	2 (0.6%)	2 (0.6%)	4 (0.6%)
SF4	Grapes, strawberries, blueberries	46 (9.0%)	38 (8.3%)	84 (8.7%)	42 (13.6%)	36 (11.5%)	78 (12.5%)	57 (8.2%)	40 (5.8%)	97 (7.0%)	35 (10.4%)	22 (6.5%)	57 (8.5%)
SF5	Melon	29 (5.7%)	23 (5.0%)	52 (5.4%)	23 (7.5%)	12 (3.8%)	35 (5.6%)	25 (3.6%)	20 (2.9%)	45 (3.2%)	18 (5.4%)	8 (2.4%)	26 (3.9%)
