# Supplementary material for: Trends in Food and Beverage Portion Sizes in Australian Children; a Time-Series Analysis Comparing 2007 and 2011–2012 National Data
Source: Children (Basel). 2017 Aug 4;4(8):69. doi: 10.3390/children4080069 (PMC5575591; doi:10.3390/children4080069)
Supplement: Supplementary file 1 [file children-04-00069-s001.zip › Table S1_adj.pdf]

Table S1: Categorization of NNS 1995, ANCNPAS 2007 and NNPAS 2011-12 foods

Question	FFQ Item	NNS (1995) Foods	ANCNPAS (2007) Foods	NNPAS (2011-12) Foods
D1	DIET soft drink eg. Diet coke	Soft drinks, artificially sweetened	Soft drink, intense sweetened	Soft drink, intense sweetened or diet<br>Soft drink, energy drink, intense sweetened
D2	Soft drink (NOT DIET) including flavoured mineral water eg. lemonade, coke, fanta, flavoured mineral water	Total non-diet soft drink/flavoured mineral water	Water, mineral, with 5%.<br>Water, mineral, fruit flavours<br>Soft drink	Soft drink, regular<br>Soft drink, energy drink<br>Mineral water, (non-)citrus flavoured, regular
D3	Water - including bottled water. unflavoured mineral water, tap water	Water, …	Water, tap<br>Water, sports type<br>Water, rainwater<br>Water, mineral, natural<br>Water, carbonated or soda<br>Water, bottled..<br>Water, bore	Mineral water, natural, unflavoured<br>Water, bore<br>Water, bottled, carbonated<br>Water, bottled, still<br>Water, filtered<br>Water, rainwater or tank water<br>Water, tap
D4	Fruit juice-based drinks, eg. orange juice or Popper	Single fruit juices	Juice concentrate,<br>Juice,<br>Vegetable blend –Juice Plus	Fruit drink, commercial<br>Juice, commercial<br>Juice, fruit blend<br>Juice, fruit & vegetable blend
D5	Cordial or 'make up' eg. Cottee's crush, raspberry	Fruit-based or flavoured cordials and drinks	Cordial,<br>Drink, fruit flavoured,<br>Fruit drink<br>Fruit puree drink,	Cordial base, regular<br>Cordial base, intense sweetened or diet<br>Cordial, recommended dilution
D6	Tea or Coffee	Tea, coffee or coffee substitutes	Coffee mix,<br>Coffee,<br>Tea,	Tea, without milk<br>Coffee,<br>Coffee mix
D7	Beer	Beers	Beer, lager<br>Beer, reduced alcohol or light style	Beer, lager or ale style,<br>Beer, flavoured,

Question	FFQ Item	NNS (1995) Foods	ANCNPAS (2007) Foods	NNPAS (2011-12) Foods
				Beer, home-brewed,<br>Beer, stout
D8	Wine or wine coolers eg. West Coast cooler	Wine, ….	Wine	Wine cooler,<br>Wine,<br>Port<br>Sherry,<br>Mirin
D9	Spirits eg. vodka, bourbon	Brandy, …<br>Whiskey, …<br>Gin, …<br>Rum, …<br>Vodka, …<br>Spirit, …<br>Liqueur, …<br>Mixed Drink, ..	Alcoholic Soda<br>Liqueur<br>Rum, dark & light coloured<br>Whisky<br>Vodka	Ouzo<br>Tequila<br>Vodka<br>Whisky or scorch<br>Spirit,<br>Liqueur,<br>Cointreau<br>Mixed drink<br>Gin<br>Brandy<br>Bitters<br>Rum, dark & light coloured
DF1 + DF3	Soy Milk	Milk - soy (unflavoured)	Soy beverage, unflavoured	Soy beverage
DF1 + DF3	Whole Milk	Milk - fat increased/whole milk	Milk, cow, fluid, extra creamy<br>Milk, cow, fluid, regular fat	Milk, cow, fluid, regular fat<br>Milk, cow, fluid, lactose free<br>Toddler milk, regular<br>Milk, cow, fluid, unflavoured
DF1 + DF3	Reduced Fat Milk	Milk - fat reduced	Milk, cow, fluid reduced fat -1-2%-<br>Milk, cow, fluid, reduced fat	Milk, cow, fluid, reduced fat<br>Milk, cow, fluid, lactose free
DF1 + DF3	Skim Milk	Milk, fluid, skim, non-fat	Milk, cow, fluid, skim	Milk, cow, fluid, skim
DF2	Flavoured milk eg. Moove, Oak, hot chocolate, milkshake, thickshake, smoothie	Flavoured milks	Beverage, banana or similar flavour<br>Beverage, caramel, honeycomb or strawberry flavour<br>Beverage, chocolate flavour<br>Beverage, drinking chocolate	Milk, cow, fluid, flavoured<br>Milkshake<br>Smoothie, cow's milk, all flavours<br>Thickshake

Question	FFQ Item	NNS (1995) Foods	ANCNPAS (2007) Foods	NNPAS (2011-12) Foods
			Beverage, from unfortified base<br>Beverage, malt flavour<br>Milk, cow, fluid, flavoured<br>Milkshake	Beverage
DF4	Cream or sour cream	Total cream	Cream, sour<br>Cream, whipped	Cream<br>Cream, dairy<br>Cream, whipped<br>Cream, sour
DF5	Ice cream - vanilla, chocolate, strawberry, sundaes, cones	ice cream -vanilla rich/regular fat<br>ice cream -other flavour regular fat/rich<br>ice cream -reduced fat<br>ice cream –other	Ice cream sundae<br>Ice cream, reduced fat<br>Ice cream, regular fat<br>Ice cream, soft serve	Ice cream, premium or rich<br>Ice cream, regular fat<br>Ice cream, low fat<br>Ice cream, fast food style<br>Sundae, vanilla ice cream
DF6	Frozen yoghurt	Frozen yoghurts, all types	Yoghurt, frozen	Yoghurt, frozen, regular fat
DF7	Yoghurt (not frozen) plain or flavoured eg. Ski, Yoplait, Vaalia	Total yoghurt	Yoghurt, (except frozen)	Yoghurt, Greek style<br>Yoghurt, natural<br>Yogurt, flavoured
DF8	Cottage cheese or ricotta	Total cottage and ricotta cheese	Cheese, cottage<br>Cheese, ricotta..	Cheese, cottage<br>Cheese, ricotta
DF9	Cheese including cheese on sandwiches, biscuits or on toast	Total other cheese	Cheese, blended<br>Cheese, cheddar<br>Cheese, Colby<br>Cheese, Feta	Cheese, blended<br>Cheese, cheddar<br>Cheese, Colby style<br>Cheese, feta<br>Cheese, mozzarella
DF10	Cheese spread, cream cheese	Total cream cheese or cheese spread	Cheese spread,<br>Cheese, cream<br>Cheese, spread	Cheese spread<br>Cheese, cream
B1	Muesli	Total muesli	Muesli, commercial,<br>Muesli, homemade	Muesli, commercial<br>Muesli, homemade
B2	Cooked porridge	Breakfast cereal, hot porridge type	Oats,<br>Porridge,	Oats, rolled<br>Porridge, rolled oats

Question	FFQ Item	NNS (1995) Foods	ANCNPAS (2007) Foods	NNPAS (2011-12) Foods
				Oat bran
B3	Breakfast cereal eg. Weet-bix, Nutri-grain, Cornflakes Sultana Bran	Breakfast cereal, low added sugar, puffed, flakes, extruded etc. single cereal — corn, rice<br>Breakfast cereal, wheat-based biscuits and shredded wheat<br>Breakfast cereal, wheat, low added sugar, puffed, flakes<br>Breakfast cereal, wheat, with added fruit or nuts<br>Breakfast cereal, grain mixtures<br>Breakfast cereal, grain and fruit/nut mixtures<br>Breakfast cereal, other sweetened	Breakfast Cereal,	Breakfast cereal
B5	Bread, pita bread, roll or toast all types – White	Bread, white<br>Bread roll, white	Bread, from white flour<br>Bread roll, from white flour<br>Bread or bread roll, from white flour	Bread, from white flour<br>Bread roll, from white flour<br>Bread or bread roll, topped<br>Bread, from wheat flour, added dried fruit
B5	Bread, pita bread, roll or toast all types – Mixed Grain	Bread, mixed grain<br>Bread roll, mixed grain<br>Bread, light rye/black/pumpernickel	Bread, mixed grain<br>Bread roll, mixed grain<br>Bread or bread roll, mixed grain	Bread, mixed grain<br>Bread roll, mixed grain
B5	Bread, pita bread, roll or toast all types - Wholemeal	Bread, wholemeal<br>Bread roll, wholemeal	Bread, from wholemeal flour<br>Bread roll, from wholemeal flour<br>Bread or bread roll, from wholemeal flour	Bread, from rye flour<br>Bread, pumpernickel<br>Bread, from spelt flour<br>Bread, from wholemeal flour<br>Bread roll, from wholemeal flour<br>Bread, from white & wholemeal flour
B6	English muffin, bagel or crumpet	English muffins/crumpets and bagels	Bagel<br>Crumpet,<br>Muffin, English-style,<br>Muffin, savoury,	Bagel<br>Crumpet<br>Muffin, English style
B7	Rice	Rice and rice products	Rice, brown<br>Rice, flavoured	Rice, white<br>Rice, brown

Question	FFQ Item	NNS (1995) Foods	ANCNPAS (2007) Foods	NNPAS (2011-12) Foods
			Rice, purchased as	Rice, flavoured
			Rice, white	Rice, purchased as
				Rice, red
				Rice, wild
				Rice, cooked
				Paella
				Risotto
B8	Other grains eg. cous cous, burghul		Couscous,	Bulgur
			Cornmeal –polenta	Cornmeal
			Millet, puffed	Couscous
				Quinoa
				Semolina
B9	Noodles eg. egg noodles (yellow), rice noodles (white)	Noodles, Asian style	Noodle,	Noodle,
B10	Pasta eg. spaghetti, lasagne, pasta bake	Pasta and egg noodles/filled pasta, ravioli	Gnocchi, potato, boiled	Noodles & pasta
			Macaroni cheese,	Pasta, gluten free
			Pasta or noodles, buckwheat flour	Pasta, maize flour
			Pasta, … filled	Pasta, white wheat flour
			Pasta, white	Pasta, wholemeal wheat flour
			Pasta, wholemeal	Pasta, unfilled
				Pasta, filled
				Gnocchi
				Lasagne (not bolognese)
				Macaroni & cheese
				Pasta dish (not bolognese)
				Tuna mornay
				Spaghetti
				Salad, pasta
S1	Cakes, sweet muffins, scones, pikelets, pancakes,	Cake,….	Brownie, chocolate	Cake or cupcake
		Bun,….	Cake,	Cake,
		Muffin,…..	Cookies,	Doughnut

Question	FFQ Item	NNS (1995) Foods	ANCNPAS (2007) Foods	NNPAS (2011-12) Foods
	hot cakes eg. apple muffin, chocolate cake, lamington	Scone,…..<br>Batter-based product,….	Doughnut<br>Hotcake,<br>Muffin, cake or American style<br>Pancake, plain<br>Pikelet or drop scone,<br>Scone,	Muffin, cake-style<br>Crepe or pancake<br>Pikelet<br>Rock cake<br>Scone<br>Slice, brownie<br>Waffle
S2	Sweet pies or sweet pastries eg. apple pie, Danish	Sweet pastry, ….<br>Tart, ….<br>Slice, ….	Baklava<br>Bun or scroll,<br>Croissant,<br>Danish style pastry,<br>Dumpling, sweet<br>Éclair, chocolate<br>Pie, apple<br>Pie, apricot<br>Pie, lemon<br>Pie, pumpkin<br>Pie, pecan<br>Pie, fruit -<br>Tart,<br>Slice,	Croissant<br>Danish<br>Éclair or profiterole<br>Baklava<br>Dumpling, sweet<br>Pie, sweet<br>Strudel<br>Tart<br>Turnover, apple<br>Slice (not brownie)<br>Bun, sweet<br>Pastry, shortcrust style
S3	Other puddings or desserts (not ice cream)<br>eg. chocolate mousse, sticky date pudding	Custards, and sweet sauces, milk-based<br>Dairy desserts, smooth or gelatin-based dairy desserts<br>Sugar-based desserts, …	Crumble,<br>Custard, dairy,<br>Custard, egg,<br>Dairy or yoghurt dessert,<br>Dessert, Bavarian cream,<br>Fudge,<br>Mousse,<br>Pudding,<br>Rum balls<br>Trifle, homemade<br>Souffle, berry	Crumble<br>Custard, dairy<br>Custard, egg<br>Custard or yoghurt dessert<br>Custard pudding<br>Dairy dessert<br>Mousse, chocolate<br>Panna cotta<br>Pudding<br>Tiramisu<br>Meringue<br>Souffle, chocolate

Question	FFQ Item	NNS (1995) Foods	ANCNPAS (2007) Foods	NNPAS (2011-12) Foods
				Souffle, fruit<br>Trifle<br>Pavlova, plain
S4	Plain sweet biscuits eg. Arrowroot, Morning Coffee, Tiny Teddies	Biscuit, sweet, plain …<br>Biscuit, sweet, fruit filled ….	Biscuits, shortbread style<br>Biscuits, sugar-glazed pastry<br>Biscuits, sweet, Anzac<br>Biscuits, sweet, chocolate chip<br>Biscuits, sweet, dried fruit filling<br>Biscuits sweet, ginger flavoured<br>Biscuits, sweet, iced<br>Biscuits, sweet, jam filled<br>Biscuits, sweet, macaroon<br>Biscuit, sweet, oatmeal<br>Biscuit, sweet, plain<br>Biscuit, sweet, sponge finger<br>Biscuit, sweet, wheatmeal<br>Biscuit, sweet, with dried fruit<br>Biscuit, sweet, with nuts	Biscuit, sweet (not cream or chocolate)
S5	Cream or chocolate biscuits eg. Tim Tams, shortbread cream	Sweet biscuits, cream-filled<br>Sweet biscuits, chocolate-coated, chocolate chip	Biscuits, sweet, chocolate coated<br>Biscuits, sweet, chocolate flavour.<br>Biscuits, sweet, chocolate topped<br>Biscuit, sweet, cream filled	Biscuit, sweet, chocolate flavoured<br>Biscuit, sweet, sandwich<br>Biscuit, sweet, wheatmeal, chocolate-coated<br>Biscuit, sweet, chocolate<br>Biscuit, sweet, cream assorted<br>Biscuit, sweet, plain, chocolate-coated
S6	Dry or savoury biscuits, crispbread, crackers eg. Vita Weats, Saos, Jatz, Shapes, rice crackers, Cruskits	Total savoury biscuits	Biscuit, savoury cake<br>Biscuit, savoury cracker<br>Biscuit, savoury crispbread<br>Biscuit, savoury<br>Biscuit, sweet, vanilla	Biscuit, savoury, corn cake<br>Biscuit, savoury, rice cake<br>Biscuit, savoury, rice cracker<br>Biscuit, savoury, corn, crispbread<br>Biscuit, savoury, from rye flour<br>Biscuit, savoury, from white wheat flour<br>Biscuit, savoury, from wholemeal wheat flour

Question	FFQ Item	NNS (1995) Foods	ANCNPAS (2007) Foods	NNPAS (2011-12) Foods
				Biscuit, savoury, rice flour
S7	Savoury combination snacks - biscuits and cheese eg. Le Snak, Snack abouts	-	Savoury snack, biscuit and cheese, …	Cracker, with cheddar cheese<br>Cracker, with cheese<br>Snack pack, savoury
S8	Sweet combination snacks eg. Dunkaroos	-	-	-
S9	Snack noodles eg. 2-minute noodles, Monster noodles	Snack, noodle-based	Noodle, wheat, instant	Noodle, wheat, instant, flavoured<br>Noodle, wheat, instant, unflavoured<br>Noodle, wheat, instant, low fat
S10	Fruit bars eg. Roll Ups	Fruit bar and fruit-based confectionery	Fruit leather	Fruit bar
S11	Snack bars eg. K-time twist bar	-	Bar, fruit filled<br>Bar, snack	Bar, snack-style<br>Bar, fruit, nut & seed<br>Muffin bar, fruit flavoured
S12	Muesli bars eg. Yoghurt Tops	Total muesli bars	Bar, breakfast and snack style, cereal<br>Bar, muesli	Bar, muesli or snack
M1	Mince dish eg. spaghetti bolognese, rissoles, shepherd's pie, lasagne		Beef, mince<br>Cannelloni,, beef<br>Lamb, mince<br>Lasagne,<br>Pasta bake, beef<br>Pasta Bolognese	Gnocchi (bolognese)<br>Pasta dish (bolognese)<br>Lasagne, beef<br>Beef, mince<br>Meatball or rissole<br>Sauce, pasta, bolognese<br>Lamb, mince<br>Pork, mince<br>Chicken, mince
M2	Beef or lamb pieces and sauce WITHOUT vegetables eg. beef stroganoff	Mixed dishes where beef or veal is the major component<br>Mixed dishes where lamb or pork, bacon, ham is the major component	Beef, curry<br>Beef, stir-fry<br>Beef, casserole or stew<br>Lamb, curry<br>Lamb, stewed or casseroled	Curry or casserole<br>Curry,<br>Stir-fry<br>Casserole

Question	FFQ Item	NNS (1995) Foods	ANCNPAS (2007) Foods	NNPAS (2011-12) Foods
M3	Beef or lamb pieces and *sauce* WITH vegetables eg. stir fry		Pork, stir-fry<br>Pork, sweet and sour	
M5	Plain meat (beef or lamb) (eg. roast, chops, steak) WITH vegetables or salad			
M11	Pork pieces and *sauce* WITHOUT vegetables eg. sweet and sour pork			
M12	Pork pieces and *sauce* WITH vegetables eg. stir fry			
M14	Plain pork (eg. roast or chops) WITH vegetables			
M4	Plain meat (beef or lamb) (eg. roast, chops, steak) WITHOUT vegetables or salad	Beef<br>Lamb<br>Veal	Beef, (except mince)<br>Lamb,<br>Veal,	Beef (no mince)<br>Lamb (no mince)<br>Veal
M6	Chicken pieces and *sauce* WITHOUT vegetables eg. satay chicken	Mixed dishes where poultry or game is the major component	Chicken, salad<br>Chicken, stew or casserole<br>Chicken, stir fry<br>Chicken, curry	Stir-fry,<br>Casserole<br>Curry

Question	FFQ Item	NNS (1995) Foods	ANCNPAS (2007) Foods	NNPAS (2011-12) Foods
M7	Chicken pieces and *sauce* WITH vegetables eg. stir fry			
M10	Plain chicken (eg. roast or BBQ) WITH vegetables			
M8	Chicken crumbed eg. chicken nuggets, KFC pieces, schnitzel	Chicken, crumbed ….	Chicken, nugget<br>Chicken, various pieces<br>Chicken,….., crumbed	Chicken, nugget<br>Chicken, schnitzel<br>Chicken,…coated<br>Chicken,…breadcrumb coating<br>Chicken, kiev
M9	Plain chicken (eg. roast or BBQ) WITHOUT vegetables	Chicken<br>Other poultry	Chicken, breast<br>Chicken, baked<br>Chicken, cooked<br>Chicken, drumstick<br>Chicken, grilled.<br>Chicken, kebab<br>Chicken, thigh<br>Chicken, whole<br>Chicken, wing	Chicken, barbecued<br>Chicken, breast<br>Chicken, drumstick<br>Chicken, fillet or kebab<br>Chicken, Maryland<br>Chicken, thigh<br>Chicken, wing<br>Chicken, whole<br>Chicken, flesh
M13	Plain pork (eg. roast or chops) WITHOUT vegetables	Pork	Pork	Pork,
M15	Liver – beef, calf, chicken (including paté)	Organ meats and offal, products and dishes	Chicken, liver<br>Pate	Beef, liver<br>Chicken, liver<br>Veal, liver<br>Pate de foie<br>Pate

Question	FFQ Item	NNS (1995) Foods	ANCNPAS (2007) Foods	NNPAS (2011-12) Foods
M16 + M17	Fish crumbed or battered and fish not crumbed or battered	Barramundi, …<br>Blackfish, …<br>Blue Grenadier, …<br>Bream, …<br>Cod, …<br>Trout, …<br>Dory, …<br>Flathead, …<br>Flounder, …<br>Kingfish, …<br>Leather jacket, …<br>Mullet, ..<br>Mackerel, …<br>Perch, …<br>Salmon, …<br>Shark, ….<br>Snapper, …<br>Tailor, …<br>Trevally, …<br>Tuna, …<br>Whiting, …<br>Fish, …	Bassa –basa-,<br>Blue grenadier – hoki -,<br>Bream<br>Fish finger, …<br>Fish,<br>Flathead,<br>Mullet, yelloweye,<br>Whiting,<br>Trout<br>Trevally, dory, ling, cod, flounder or sole<br>Snapper,<br>Shark-flake- skinless fillet,<br>Sliver perch,<br>Barramundi<br>Cod,<br>Mulloway,<br>Salmon,<br>Tuna, yellowfin steaks	Barramundi<br>Bassa<br>Blue grenadier<br>Brean<br>Blue-eye trevalla<br>Cod or hake<br>Flathead<br>Flounder<br>John dory<br>Ling<br>Gemfish<br>Grouper<br>Milkfish<br>Morwong<br>Mullet<br>Mulloway<br>Mackerel<br>Nile perch<br>Orange roughly<br>Salmon<br>Shark<br>Silver perch<br>Snapper<br>Swordfish<br>Tilapia<br>Trevally or kingfish<br>Trout<br>Tuna<br>Whiting<br>Fish,<br>Fish finger

Question	FFQ Item	NNS (1995) Foods	ANCNPAS (2007) Foods	NNPAS (2011-12) Foods
M18	Canned tuna, salmon, sardines including patties	Anchovy, canned …..<br>Herring, canned ….<br>Mackeral, canned …<br>Salmon, …, canned …<br>Sardine, canned …<br>Tuna, canned …..	Anchovy, canned<br>Salmon, canned<br>Tuna, canned,<br>Tuna, flavoured, canned<br>Sardine, canned<br>Fish cake	Fish patty or cake<br>Salmon patty or cake<br>Salmon,…canned<br>Sardine,…canned<br>Tuna, canned<br>Anchovy, canned
M19	Other seafood eg. prawns, lobster	Crab, …<br>Lobster, ..<br>Bug, …<br>Prawn, …<br>Yabbie, …<br>Abalone, …<br>Calamari, …<br>Clam, …<br>Mussel, …<br>Oyster, …<br>Scallop, …	Crab, various types,<br>Crabmeat,<br>Lobster,<br>Mussel, green,<br>Oyster, baked or grilled<br>Oyster, raw<br>Prawn,<br>Squid or calamari,<br>Scallop,	Crab<br>Lobster<br>Mussel<br>Octopus<br>Oyster<br>Prawn<br>Scallop<br>Squid or calamari
M20 + M21	Creamy and clear soup (with rice or noodles)	Soup, …	Soup, …	Soup,
M22	Tacos, burritos, enchiladas	Taco and tortilla-based dishes	Burrito,<br>Enchilada,<br>Taco,	Taco shell<br>Mexican taco<br>Mexican wrap<br>Bread, tortilla<br>Chicken wrap<br>Wrap,
M23	Sausages, frankfurts, Pluto Pup	Sausage<br>Frankfurts, and saveloys	Frankfurt or cheerios,<br>Sausage,	Sausage,<br>Frankfurt<br>Saveloy
M24	Hamburger - all types	Chickenburger<br>Fishburger<br>Hamburger	Chickenburger<br>Fishburger,<br>Hamburger,	Chicken burger<br>Chicken roll<br>Hamburger

Question	FFQ Item	NNS (1995) Foods	ANCNPAS (2007) Foods	NNPAS (2011-12) Foods
				Fish burger
M25	Pizza	Pizza	Pizza,	Pizza,
M26	Pie, sausage roll, chiko roll	Chiko roll<br>Meat pie<br>Sausage roll	Chiko roll, deep fired<br>Pie, meat.<br>Pie, steak<br>Sausage roll,	Pie, savoury<br>Sausage roll<br>Chiko roll
M27	Hot dog	Hot dog	-	Hot dog,
M28	Savoury pastries eg, spinach and cheese triangles	Other savoury pastries	Cannelloni, spinach and ricotta<br>Curry puff, beef,<br>Dim sim, meat and vegetable filling<br>Dumpling, meat filled<br>Dumpling, prawn filled<br>Pastie, vegetable<br>Pastry, cheese filling.<br>Pastry, spinach and cheese filling .<br>Pastry, spring roll<br>Pastry, vegetable filling	Cannelloni, spinach and ricotta<br>Curry puff<br>Dim sim<br>Dumpling or wonton<br>Pastry, puff<br>Pastry, shortcrust style<br>Pastry, spring roll<br>Pastry, filled with salmon<br>Pastry, filled with spinach<br>Pastry, filled with spinach & cheese<br>Pastry, filled with vegetables<br>Pastry, filled with vegetables & meat<br>Samosa
M29	Hash browns, potato scallops	Hash Brown,<br>Potato, gems<br>Potato, scallops	Potato, gems<br>Potato, hash brown<br>Potato, mashed & crumbed<br>Potato, scallop	Potato, hash brown<br>Potato scallop<br>Potato, gem
O1	Chips (not potato) eg. Twisties, corn chips, burger rings	Corn snacks and extruded snacks	Corn chips<br>Crisp or chip, soy<br>Crisp or chip, vegetable,<br>Crisp, savoury	Beetroot crisps or chips<br>Chip or crisp, soy<br>Corn chips<br>Extruded snack<br>Grain waves wheat snack<br>Noodle snack<br>Pappadam<br>Popcorn

Question	FFQ Item	NNS (1995) Foods	ANCNPAS (2007) Foods	NNPAS (2011-12) Foods
				Pretzels<br>Rice crisps or chips<br>Vegetable crisps or chips
O2	Potato chips or crisps eg. plain, salt and vinegar	Potato crisps	Crisp or chip, potato<br>Potato straws	Potato crisps or chips
O3	Ice block – creamy eg. Paddle Pop, Magnum, Cornetto	Ice cream, individual bar, stick and cone varieties	Ice cream bar<br>Ice cream, stick	Ice cream, bar<br>Ice cream, sandwich<br>Ice cream, stick<br>Ice cream cone<br>Ice cream,<br>Ice confection, stick, milk-based
O4	Ice block – water eg. Frosty Fruit, lemonade	Water ice confection, gelato	Gelato, various flavours, commercial<br>Ice confection, stick	Ice confection, stick, water-base<br>Ice confection, stick or tub, fruit juice or fruit flavoured<br>Gelato or sorbet
O5	Chocolate eg. plain chocolate, Mars Bar, Snickers, Milky Way	Chocolate and chocolate-based confectionery	Chocolate or chocolate bar<br>Chocolate<br>Rocky Road<br>Turkish delight	Bar,<br>Chocolate<br>Confectionery
O6	Lollies without chocolate eg. lollipops, snakes, Skittles, Starburst	Other confectionery	Caramels<br>Honeycomb, plain<br>Liquorice<br>Marshmallow<br>Sugar confectionary	Honeycomb<br>Liquorice<br>Lolly<br>Lollipop or chupa chup<br>Marshmallow<br>Confectionery (no chocolate)<br>Turkish delight<br>Fruit, leather
O7	Low fat salad dressing or mayonnaise	Mayonnaise and salad dressing - low fat	Dressing, reduced fat<br>Mayonnaise, low fat	Dressing,…reduced/low fat<br>Dressing, vinegar based<br>Mayonnaise,…reduced/low fat

Question	FFQ Item	NNS (1995) Foods	ANCNPAS (2007) Foods	NNPAS (2011-12) Foods
O8	Salad dressing or mayonnaise - not low fat	Mayonnaise and salad dressing - full fat	Dressing, regular<br>Mayonnaise, full fat	Dressing,…regular fat<br>Mayonnaise,…regular fat
O9	Nuts eg. peanuts, almonds	Nuts (excluding coconut)	Nut<br>Nuts, mixed	Nut<br>Mixed nuts
O10	Jam, honey, golden syrup, marmalade	Honey, jam, lemon butter	Honey<br>Jam<br>Marmalade<br>Syrup, maple.<br>Syrup, golden	Jam<br>Marmalade<br>Honey<br>Syrup, agave<br>Syrup, golden<br>Syrup, maple
O11	Peanut butter, Nutella	Peanut butter, chocolate and hazelnut spread	Peanut butter, smooth & crunchy<br>Spread, hazelnut & chocolate flavoured	Peanut butter<br>Spread, hazelnut & chocolate flavoured
O12	Vegemite, Mighty Mite, Promite, Marmite	-	Spread, yeast	Spread, yeast<br>Spread, vegetable & yeast extract
O13	Tomato sauce, barbecue sauce	Tomato and barbeque sauce	Sauce, tomato<br>Sauce, barbecue	Sauce, tomato<br>Sauce, barbecue
O14	Devon, salami	Salami, chicken roll, stassburg, luncheon meats	Kabana or cabanossi<br>Salami	Chicken, processed luncheon meat<br>Devon, processed luncheon meat<br>Salami
O15	Bacon, ham	Bacon<br>Ham	Bacon<br>Ham	Bacon<br>Ham<br>Prosciutto
O16	Eggs eg. boiled, scrambled	Total eggs, cooked	Egg	Egg, chicken<br>Omelette, chicken egg
O17	Jelly	Jelly, made up	Jelly, made up	Jelly, sugar sweetened<br>Jelly, intense sweetened<br>Jelly crystals,

Question	FFQ Item	NNS (1995) Foods	ANCNPAS (2007) Foods	NNPAS (2011-12) Foods
F1 + F2	Hot chips bought from a shop (eg. McDonald's fries) or cooked at home	Potato - hot chips	Potato, chips<br>Potato, fries<br>Potato, wedged	Potato, chips<br>Potato, fries<br>Potato, wedges
F3	Potato boiled, mashed, baked	Potato - boiled, mashed, baked	Potato, baked<br>Potato, boiled<br>Potato, mashed,<br>Potato, peeled,<br>Potato, unpeeled	Potato, Sebago<br>Potato, mashed<br>Potato, peeled<br>Potato, unpeeled<br>Potato, coliban<br>Potato, desiree<br>Potato, new<br>Potato, Pontiac<br>Potato, pale skin<br>Potato, red skin
F4	Pumpkin	Pumpkin	Pumpkin, peeled	Pumpkin, peeled<br>Pumpkin, unpeeled<br>Pumpkin, butternut<br>Pumpkin, golden nugget<br>Pumpkin, Jarrahdale<br>Pumpkin, Queensland blue
F5	Sweet potato	Sweet potato	Sweet potato,	Sweet potato, orange flesh<br>Sweet potato, white flesh
F6	Cauliflower	Cauliflower	Cauliflower,	Cauliflower, fresh or frozen
F7	Green beans	Beans - green and snake	Bean, green<br>Bean, broad<br>Beans, not further specified	Bean, broad<br>Bean, green
F8	Spinach	Spinach and silverbeet	Mixed greens<br>Spinach<br>Silverbeet	Silverbeet<br>Spinach<br>Mixed leafy greans
F9	Cabbage or brussel sprouts	Cabbage and brussel sprouts	Brussels sprout<br>Cabbage	Brussels sprout<br>Cabbage, red<br>Cabbage, white

Question	FFQ Item	NNS (1995) Foods	ANCNPAS (2007) Foods	NNPAS (2011-12) Foods
				Cabbage, boiled<br>Cabbage, chinese<br>Cabbage, mustard<br>Cabbage, savoy<br>Cabbage, baked
F10	Peas	Peas (including snow peas)	Pea, green<br>Pea, split<br>Snow pea	Pea, green<br>Snow pea<br>Pea, split
F11	Broccoli	Broccoli	Broccoli	Broccoli, fresh or frozen
F12	Carrots	Carrots	Carrot	Carrot, baby<br>Carrot, matur<br>Carrot, peeled or unpeeled
F13	Zucchini, eggplant, squash	Squash<br>Zucchini<br>Eggplant	Eggplant<br>Okra<br>Zucchini<br>Squash	Eggplant, peeled or unpeeled<br>Squash<br>Zucchini, golden<br>Zucchini, green skin
F14	Capsicum	Capsicum	Capsicum	Capsicum, green<br>Capsicum, red<br>Capsicum, fresh or frozen
F15	Corn, sweetcorn, corn on the cob	Corn	Sweetcorn	Sweetcorn, baby<br>Sweetcorn, fresh or frozen<br>Sweetcorn, kernels<br>Sweetcorn, creamed
F16	Mushrooms	Mushrooms	Mushroom	Mushroom, common<br>Mushroom, oriental<br>Mushroom, fresh or frozen<br>Mushroom, canned in brine
F17	Tomatoes	Tomatoes (includes canned and sun dried)	Tomato, common	Tomato, common<br>Tomato, raw<br>Tomato, fresh<br>Tomato, cherry or grape<br>Tomato, hydroponic

Question	FFQ Item	NNS (1995) Foods	ANCNPAS (2007) Foods	NNPAS (2011-12) Foods
				Tomato, roma<br>Tomato, sundried or semi-sundried<br>Tomato, whole
F18	Lettuce	Lettuce	Lettuce, raw	Lettuce, raw<br>Lettuce, cos<br>Lettuce, iceberg<br>Lettuce, mignonette<br>Lettuce, baked<br>Lettuce, boiled
F19	Celery, cucumber	Celery<br>Cucumber	Celery<br>Cucumber	Celeriac<br>Celery<br>Cucumber, common<br>Cucumber, peeled or unpeeled<br>Cucumber, apple crystal<br>Cucumber, Lebanese<br>Cucumber, telegraph
F20	Avocado	Avocados	Avocado, raw	Avocado, raw<br>Avocado, cooked
F21	Onion, spring onion, leek	Onions, leeks, spring onions and shallots	Onion, mature<br>Onion, spring Shallot<br>Leek	Leek<br>Onion, mature<br>Onion, spring<br>Shallot
F22	Soybeans, tofu	Soy beans<br>Tofu	Bean, soya<br>Tofu –soy bean curd	Tofu (soy bean curd)<br>Bean, soya
F23	Baked beans	Baked beans	Baked beans	Baked beans
F24	Other beans, lentils eg. chickpeas, split peas	Other mature legumes and pulses<br>Other legume and pulse products<br>Other Beans - broad, butter, kidney	Bean, black<br>Bean, butter<br>Bean, lima<br>Bean, mixed<br>Bean, red kidney<br>Beans, refried<br>Chickpea<br>Lentil	Bean, black<br>Bean, cannellini<br>Bean, lima<br>Bean, mixed<br>Bean, red kidney<br>Bean, refried<br>Chickpea<br>Lentil, dried

Question	FFQ Item	NNS (1995) Foods	ANCNPAS (2007) Foods	NNPAS (2011-12) Foods
				Lentil, green or brown<br>Lentil, red
F25	Canned fruit eg. peaches, Two fruits	Fruit - canned, mixed<br>Fruit - canned single<br>Total canned fruit	Apple, canned in syrup<br>Apricot, canned<br>Peach, canned<br>Pear, canned<br>Fruit, canned	Apple, canned or puree<br>Apricot, canned...<br>Fruit, puree or stewed<br>Mixed fruit<br>Peach, canned…<br>Pear, canned…<br>Pineapple, canned…<br>Strawberry, canned…<br>Mixed berry, puree
F26	Fruit salad	Fruit salad (not canned)	Fruit salad	Fruit salad,
F27	Dried fruit eg. sultanas, dried apricots	Dried fruit, preserved fruit	Apple, dried<br>Apricot, dried<br>Banana chip<br>Blueberry, dried<br>Berries, mixed -strawberry, raspberry, blueberry, blackberry-, dried<br>Cranberry, dried<br>Currant, dried<br>Date, dried<br>Fig, dried<br>Mango, dried<br>Mixed fruit, dried<br>Pineapple, dried<br>Prune –dried plum<br>Sultana, dried	Apple, dried<br>Apricot, dried<br>Banana chip<br>Cranberry, dried<br>Currant, dried<br>Date, dried<br>Fig, dried<br>Mixed dried fruit<br>Prune (dried plum)<br>Sultana
F28	Apple or pear	Total apple and pear	Apple, green skin<br>Apple, peeled, raw<br>Apple, red skin<br>Pear, raw	Apple, green skin<br>Apple, red skin<br>Apple, peeled<br>Apple, unpeeled<br>Apple, raw<br>Apple, fuji

Question	FFQ Item	NNS (1995) Foods	ANCNPAS (2007) Foods	NNPAS (2011-12) Foods
				Apple, golden delicious<br>Apple, granny-smith<br>Apple, Jonathon<br>Apple, pink lady<br>Apple, red delicious<br>Apple, royal gala<br>Apple, bonza<br>Pear, nashi,<br>Pear, peeled<br>Pear, unpeeled<br>Pear, raw<br>Pear, brown<br>Pear, packhams triumph<br>Pear, william bartlett
F29	Orange, mandarin, grapefruit	Total oranges, mandarin, grapefruit	Mandarin<br>Grapefruit<br>Orange	Orange, navel (all varieties)<br>Orange, Valencia<br>Orange, peeled<br>Mandarin<br>Grapefruit
F30	Banana	Bananas	Banana, cavendish, peeled, raw	Banana, Cavendish<br>Banana, lady finger or sugar
SF1	Peach, nectarine, plum or apricot	Apricot<br>Nectarine<br>Peach<br>Plum	Apricot, fresh, raw<br>Nectarine, unpeeled, raw<br>Peach, fresh, raw<br>Plum	Peach, yellow<br>Peach, white<br>Peach, peeled<br>Peach, unpeeled<br>Nectarine, yellow<br>Nectarine, white<br>Plum<br>Apricot
SF2	Mango or paw-paw	Total mango and pawpaw	Mango, peeled<br>Pawpaw	Mango<br>Pawpaw (papaya)
SF3	Pineapple	Pineapples	Pineapple, fresh	Pineapple (cayenne)

Question	FFQ Item	NNS (1995) Foods	ANCNPAS (2007) Foods	NNPAS (2011-12) Foods
SF4	Grapes, strawberries, blueberries	Total grapes, strawberries, blueberries	Berries, mixed -strawberry, raspberry, blueberry, blackberry-, fresh, raw<br>Blueberry, fresh<br>Boysenberry, fresh<br>Grape, raw<br>Mulberry, fresh, raw<br>Raspberry<br>Strawberry	Grape, black muscatel<br>Grape, black sultana<br>Grape, cornichon<br>Grape, red globe<br>Grape, Thompson seedless or sultana<br>Grape, Waltham cross<br>Grape, raw<br>Mixed berry<br>Mulberry<br>Raspberry<br>Strawberry<br>Blackberry<br>Blueberry
SF5	Melon eg. watermelon, rockmelon, honeydew melon	Total melon	Melon	Melon, honey dew<br>Melon, rockmelon (cantaloupe)<br>Melon, watermelon<br>Melon, peeled
