# Supplementary material for: Trends in Food and Beverage Portion Sizes in Australian Children; a Time-Series Analysis Comparing 2007 and 2011–2012 National Data
Source: Children (Basel). 2017 Aug 4;4(8):69. doi: 10.3390/children4080069 (PMC5575591; doi:10.3390/children4080069)
Supplement: Supplementary file 1 [file children-04-00069-s001.zip › Table S2_adj.pdf]

Table S2. Basic demographic data of participants of the 2007 ANCNPAS and the 2011-12 NNPAS

	**2007** (*n*=1491)	**2011-12** (*n*=675)	**2007** (*n*=954)	**2011-12** (*n*=578)	**2007** (*n*=966)	**2011-12** (*n*=622)	**2007** (*n*=1388)	**2011-12** (*n*=673)
**% of total *N***	**31.1**	**26.5**	**19.9**	**22.7**	**20.1**	**24.4**	**28.9**	**26.4**
**Age group (years)**	2–4	2–4	5–8	5–8	9–12	9–12	13–16	13–16
**Mean age, (SD)**	2.83 (0.74)***	2.95 (0.82)***	6.60 (1.12)*	6.48 (1.11)*	10.53 (1.10)	10.52 (1.14)	14.55 (1.01)	14.60 (1.15)
**Sex**								
Male	52	49	51	51	47	50	50	50
Female	48	51	49	49	53	50	50	50
**Weight status (%)**								
Underweight	4.4	5.8	3.7	4.2	5.6	4.0	4.1	2.4
Normal	74.6	56.1	76.2	58.1	67.0	52.4	70.4	58.4
Overweight	15.4	12.4	13.6	10.4	19.4	20.3	17.8	16.5
Obese	3.4	4.7	6.2	5.7	7.2	8.7	6.4	6.2
Unknown	2.1	20.9^{a***}	0.3	21.6^{a***}	0.8	14.6^{a***}	0.4	16.5^{a***}
**Daily energy intake (MJ)**	6.35 (1.86)	6.03 (2.02)*	7.57 (2.12)	7.20 (2.46)*	8.91 (2.80)	8.39 (2.91)**	10.02 (3.48)	9.19 (3.82)**
**Amount eaten (%)**								
Usual	97.9	84.7	97.1	82.0	72.5	77.7	72.3	70.7
Unusual	2.1	15.1^{a***}	2.9	18.0^{a***}	27.5	22.3^{a*}	27.7	29.3

[a] The distribution of the categorical variable significantly differs between 2007 and 2011-12

* *p*-value <0.05, *** *p*-value <0.001
